# Causal relationship of immune cell characteristics in hepatocellular carcinoma: A multi-omics analysis based on Mendelian randomization

**DOI:** 10.1097/MD.0000000000045942

**Published:** 2026-05-12

**Authors:** XiaoLing Tian, BaoChun Wang, XinYi Zhang, Ge Song, YuBo Liu, YuQian Gao, JinYan Wang, YunQi Hua

**Affiliations:** a Baotou Medical College, Inner Mongolia University of Science and Technology, Baotou, Inner Mongolia, China; b Department of Oncology, Baotou Cancer Hospital, Baotou, Inner Mongolia, China; c Department of Pharmacy, Binzhou Polytechnic, Binzhou, Shandong Province, China.

**Keywords:** causality, hepatocellular carcinoma, immune cells, Mendelian randomization, TCGA, tumor immune microenvironment

## Abstract

The tumor immune microenvironment of hepatocellular carcinoma (HCC) is complex, yet the causal relationship between immune cell subpopulations and HCC risk remains incompletely elucidated. This study aims to systematically evaluate the causal association between immune cell subpopulations and HCC using Mendelian randomization (MR) analysis, and to validate the biological mechanisms underlying these associations through multi-omics data. Bidirectional two-sample MR analysis was performed to examine causal relationships between 731 immune cell subpopulations and HCC. Inverse-variance weighting (IVW) served as the primary analysis method, with robustness validation through Bayesian weighted MR (BWMR) and machine learning algorithms. Therefore, for significantly associated immune subpopulations, independent analyses of gene expression, prognosis, and tumor immune microenvironment were conducted using HCC data from the Cancer Genome Atlas (TCGA) LIHC cohort. MR analysis and validation identified 21 immune cell subpopulations with significant causal associations to HCC risk. Among these, 12 were identified as risk factors, and 9 as protective factors. Validation in the TCGA cohort revealed that risk-associated immune subpopulations were predominantly enriched for markers of T cell exhaustion and immunosuppressive microenvironments, whereas protective subpopulations likely represented a distinct regulatory B cell subset whose function was associated with the anti-inflammatory factor interleukin-10. This study genetically confirms that specific immune cell functional subpopulations constitute causal risk factors for HCC. These subpopulations exert their effects by shaping distinct tumor immune microenvironments. These findings provide novel mechanisms for understanding the immunopathogenesis of HCC and identify potential targets for developing novel immune intervention strategies.

## 1. Introduction

Hepatocellular carcinoma (HCC) ranks among the most prevalent and lethal malignant tumors globally,^[1,2]^ imposing a significant burden on public health worldwide. Despite advances in therapeutic approaches, the prognosis for HCC patients remains poor, particularly for those with advanced disease.^[3]^ Given these challenges, it is of paramount importance to gain a deeper understanding of the mechanisms underlying HCC development and progression. Additionally, identifying effective prognostic biomarkers and discovering novel therapeutic targets are crucial.

The liver is not only a metabolic organ but also a crucial immune organ, rich in diverse innate and adaptive immune cells that play key roles in maintaining immune homeostasis and anti-tumor surveillance.^[4]^ The tumor immune microenvironment of HCC comprises tumor cells, immune cells, cytokines, and extracellular matrix.^[5]^ The functional state of immune cells profoundly influences tumor progression and treatment response. In recent years, immunotherapies, such as immune checkpoint inhibitors, have demonstrated potential in treating advanced HCC.^[6]^ However, their efficacy remains limited, highlighting the need for a more refined analysis of the HCC immune microenvironment.^[7,8]^ Clinical data indicate that the immune cell composition of HCC correlates with prognosis and treatment response.^[9]^ Nevertheless, significant gaps remain in understanding the underlying mechanisms of these immune networks, necessitating deeper mechanistic studies.

Traditional observational studies are susceptible to confounding factors and reverse causality, making it difficult to establish a causal relationship between immune cells and HCC. Mendelian randomization (MR) analysis utilizes genetic variants as instrumental variables to infer causal relationships between exposure and outcome, effectively overcoming these limitations.^[10,11]^ Recently, 2 studies have employed MR to explore the association between immune cells and HCC. Tang et al identified 5 immune phenotypes associated with HCC risk in European cohorts,^[12]^ while Nov et al reported 36 immune phenotypes associated with HCC risk in Japanese cohorts.^[13]^ These studies provide preliminary insights into the genetic causal link between immune cells and HCC. However, the cellular composition and functional state of the HCC immune microenvironment are highly heterogeneous, as revealed in depth by high-resolution single-cell studies.^[14]^ Most of the aforementioned MR studies remain at the level of statistical association, failing to effectively link genetic discoveries with specific immunological mechanisms within the tumor microenvironment. They also lack in-depth biological validation studies of the identified immune phenotypes in independent cohorts.

To this end, this study aims to systematically evaluate the causal relationship between 731 immune cell phenotypes and HCC risk through a large-scale dual-sample MR analysis. Compared with previous studies, the innovations of this research include: employing a more rigorous Bayesian weighted Mendelian randomization (BWMR) approach and performing cross-validation with multiple machine learning algorithms to enhance the robustness of findings; this study is the first to conduct in-depth validation of MR-identified key immune phenotypes at the transcriptomic, prognostic, and tumor immune microenvironment levels using independent public databases, such as TCGA – thereby translating statistical associations into biological insights; and furthermore, by leveraging established knowledge of the HCC immune microenvironment,^[14,15]^ the study not only focuses on risk factors but also identifies protective immune signatures, offering new perspectives for comprehensively understanding the dual role of the immune system in HCC, specifically its capacity to both promote tumor progression and mediate anti-tumor immunity.^[16]^

## 2. Methods

### 2.1. Study design

**Figure 1.**
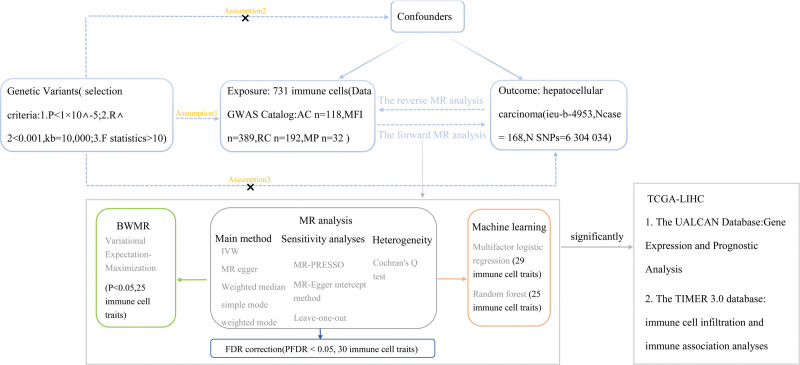
Study design. Assumption 1 denotes the correlation assumption, which states that SNPs are strongly associated with the exposure; Assumption 2 denotes the independence assumption, which states that SNPs are not influenced by confounding factors; Assumption 3 denotes the exclusion assumption, which states that SNPs affect the outcome only through the exposure. BWMR = Bayesian weighted Mendelian randomization, GWAS = genomewide association study, IVW = inverse variance weighted, MR = Mendelian randomization, SNP = single nucleotide polymorphism.

A two-sample MR method was used to evaluate the bidirectional causal relationship between 731 immune phenotypes and the risk of hepatocellular carcinoma. For MR analysis to produce valid instrumental variable estimates, the genetic variants selected as IVs must satisfy 3 assumptions: Figure 1 illustrates the specific MR study design.

### 2.2. Data source for IVs

Genome-wide association study (GWAS) statistics for the immune traits can be found in the GWAS catalogue under accession numbers GCST90001391 to GCST90002121 (https://www.ebi.ac.uk/gwas/studies/GCST90001391). The 731 immune phenotypes include median fluorescence intensity, absolute cell count (AC) and relative cell count, as well as morphological parameters (presented in Supplementary File 1, Supplemental Digital Content, https://links.lww.com/MD/Q784). This dataset was selected because it provides detailed immune cell measurement data, which is crucial for elucidating the immune environment associated with the risk of HCC. These phenotypes comprehensively cover both quantitative and morphological immune characteristics, all of which are essential for capturing the immune profile related to HCC.

We conducted an extensive GWAS involving 3757 individuals of European descent. We genotyped approximately 22 million SNPs using high-density arrays and imputed them using a reference panel from Sardinian sequences. These associations were assessed after considering covariates such as sex and age. Due to the limited scale of GWAS data on immune cells, a more lenient *P*-value of 1e−5 was used to identify the significance of each immune phenotype’s IV. SNP pruning was also performed based on linkage disequilibrium (LD within a radius of 10,000 kb with *r*^2^ < 0.001) and IVs were selected with F statistics exceeding 10 to ensure robust data.

### 2.3. Data sources for outcomes

SNPs associated with HCC were obtained from the IEU Open GWAS project (https://gwas.mrcieu.ac.uk/datasets/ieu-b-4953/). This GWAS included 372,184 individuals of European ancestry (168 cases and 372,016 controls), encompassing approximately 6,304,034 SNPs. The large sample size, together with the fact that the exposure data were derived from the same population, ensured the relevance and validity of the analysis.

### 2.4. Mendelian randomization analysis

We used inverse variance weighting (IVW) and other robust methods, including MR-Egger, weighted median, simple mode and weighted mode. IVW is the classical approach in MR analysis and calculates the weighted average using the inverse of each IV’s variance to ensure the validity of all IVs.^[17]^ We carefully examined the causal relationships between 731 immune phenotypes and HCC. Additionally, MR-PRESSO helps to identify and correct outliers of horizontal pleiotropy, thereby enhancing the integrity of our MR analysis.^[18]^ Taking into account the issue of multiple testing, we applied false discovery rate (FDR) correction to the above results, considering an FDR of <0.2 to be suggestive of a causal relationship, and an FDR of <0.05 to indicate a significant causal relationship. Only genes showing a significant MR effect on HCC according to IVW (FDR-corrected threshold *P* < .05) were included in subsequent analyses.

### 2.5. Sensitivity analysis

To further elucidate the potential pleiotropy, a sensitivity analysis was conducted, incorporating heterogeneity and horizontal multiple validity tests. A leave-one-out analysis was performed to assess the robustness of the genotype-outcome association and examine the impact of individual genetic variations on the overall results. To mitigate the possibility of false positive results, the MR-Egger intercept test was used to evaluate the pleiotropy of significant MR estimates. This test is based on the intercept term in MR-Egger regression and determines whether it significantly deviates from zero.^[19]^ However, sensitivity analysis can only be conducted for genes with multiple IVs, which may affect statistical power and increase the risk of false positives. The Cochran Q test was used to assess heterogeneity among the selected SNPs, with a value below 0.05 indicating its presence.^[20]^ Odds ratios (OR) and their 95% confidence intervals (CI) were used to measure causal relationships, and scatter plots and funnel plots were employed to verify the stability of the results, ensuring a rigorous evaluation of causal inference.

### 2.6. Identification of immune characteristics

In order to address the complexity arising from the polygenic nature and widespread pleiotropy of complex immune traits, we employed BWMR to conduct causal inference. This method explicitly accounts for uncertainty arising from weak effects caused by polygenicity, and resolves violations of IVs assumptions due to pleiotropy, via Bayesian weighted outlier detection. To improve the computational stability and efficiency of BWMR causal inference, a Variational Expectation-Maximisation algorithm was developed that is both statistically efficient and computationally stable.^[21]^ We therefore used this method to test the results of the IVW method.

In addition, to control for confounding factors, improve the reliability of the results and reflect the strength and direction of the impact of the variables on the outcomes more intuitively, multifactor logistic regression and random forest machine learning methods were employed for further identification. Multifactor logistic regression was used to establish the relationship between each immune cell phenotype and liver cancer. The basic idea is to use the output of a linear regression model mapped through a logistic function to a range between 0 and 1, thereby representing the probability of a certain event occurring.^[22]^ Random forest machine learning was used to plot the receiver operating characteristic (ROC) curve to evaluate the classification model’s performance, where a closer area under the curve (AUC) value to 1 indicates better diagnostic performance.^[23]^ By obtaining the intersection of the results from these different methods, we aimed to accurately identify immune cells associated with HCC.

### 2.7. Comprehensive validation of immunological characteristics and exploration of mechanisms

To validate MR results in an independent cohort and elucidate biological mechanisms, we conducted the following analyses:

#### 2.7.1. Selection of key immunophenotypes and core genes

Based on significance levels and biological relevance, we selected the 6 most representative phenotypes from the immune cell features identified as significant by MR analysis for in-depth validation. These included 5 risk factors and one protective factor. For each immune phenotype, we identified its core surface marker genes.

#### 2.7.2. Gene expression and prognostic analysis (based on the UALCAN database)

To validate core genes at the transcriptomic level, the UALCAN online platform (https://ualcan.path.uab.edu/)^[24]^ was utilized. This platform integrates RNA-seq and clinical data from the TCGA project, offering convenient tools for differential gene expression analysis and survival analysis. Specifically, differential expression analysis was performed for each core gene by comparing its transcripts per million levels in LIHC (liver hepatocellular carcinoma) tumor tissues (n = 371) versus normal tissues (n = 50). The platform’s built-in *t*-test was employed, with *P* < .05 indicating statistically significant differences. Additionally, survival analysis utilizes the platform’s “Survival” module to stratify patients into high- and low-expression groups based on median gene expression levels. Kaplan–Meier survival curves are plotted, and the Log-rank test is applied to compare survival differences between groups, with *P* < .05 indicating statistical significance.

#### 2.7.3. Tumor immune microenvironment analysis (based on TIMER 3.0 database)

To elucidate the association between core genes and the tumor immune microenvironment, we employed the TIMER 3.0 database (https://compbio.cn/timer3/).^[25]^ This database utilizes multiple advanced algorithms (e.g., CIBERSORT, EPIC, XCELL) to estimate immune cell infiltration abundance in TCGA samples and enables gene co-expression and immune association analyses. Immune cell infiltration analysis utilizes the “Immune Association” module to examine Spearman correlations between gene expression and infiltration levels of various immune cells (e.g., CD8^+^ T cells, regulatory T cells (Tregs), B cells, macrophages, myeloid-derived suppressor cells) calculated by multiple immune algorithms. Immune-related gene co-expression analysis, conducted under the “Gene_Corr” module, examines the expression correlations between core genes and key immune checkpoint molecules (e.g., PDCD1 (PD-1), CD274 (PD-L1), CTLA4) or other immune-related genes (e.g., interleukin-10 (IL-10), TNF).

### 2.8. Statistical analysis

Our analysis conforms to the STROBE-MR guidelines and was conducted using the R software (version 4.4.3) and packages such as “TwoSampleMR” and “ggplot2” to perform a comprehensive statistical analysis. Additionally, multi-factor regression analysis was conducted using Python libraries such as “Pandas,” “NumPy,” and “SciPy.” Gene expression validation and correlation analysis were performed using the built-in statistical methods of the UALCAN and TIMER 3.0 platforms. Statistical significance was set at a two-tailed *P* < .05.

## 3. Result

### 3.1. Investigation of the causal effect of immune cells on the risk of HCC

**Figure 2.**
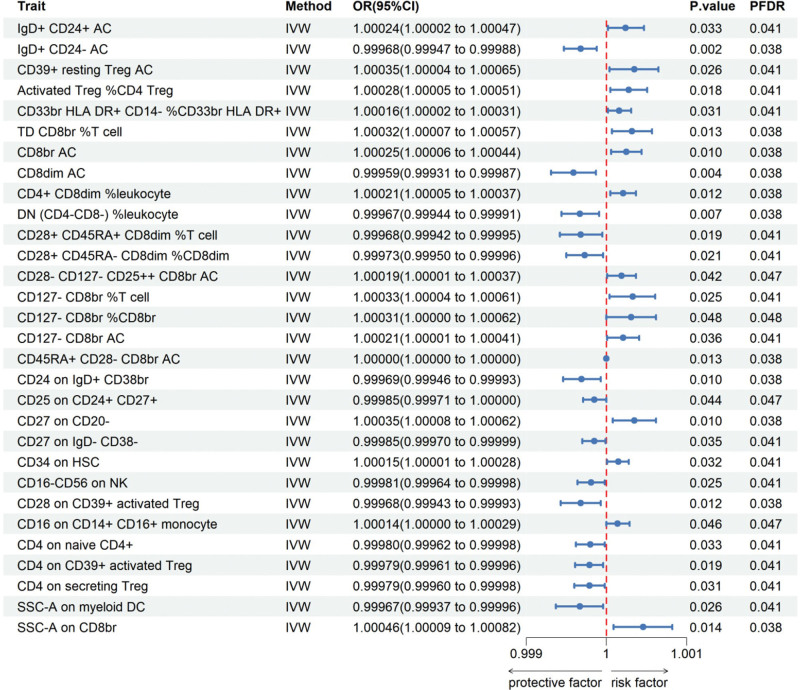
Forest plots summarizing the Mendelian randomization results of immune cells with a causal relationship to HCC. PFDR < 0.05 is the nominal significance. CI = confidence interval, FDR = false discovery rate, HCC = hepatocellular carcinoma, OR = odds ratio.

To study the causal impact of immune cells on the risk of HCC we conducted a two-sample MR analysis, using the IVW method as the primary analysis (Table 1 shows the number of SNPs screened for all positive results). After screening with the IVW method, 30 immune phenotypes were identified as significant. Following FDR testing correction (PFDR < 0.05) (Fig. 2).

**Table 1:** The number of SNPs screened for positive results.

Immune traits	ID	Number of SNPs after LD	Number of SNPs after *F* > 10	Number of final IVs
IgD^+^ CD24^+^ AC	GCST90001412	23	23	13
IgD^+^ CD24^−^ AC	GCST90001416	24	24	11
CD39^+^ resting Treg AC	GCST90001483	29	29	6
Activated Treg %CD4 Treg	GCST90001487	31	31	12
CD33br HLA DR^+^ CD14^−^ %CD33br HLA DR^+^	GCST90001519	19	19	12
TD CD8br %T cell	GCST90001559	19	19	8
CD8br AC	GCST90001592	23	23	14
CD8dim AC	GCST90001596	28	28	8
CD4^+^ CD8dim %leukocyte	GCST90001611	18	18	14
DN (CD4^−^CD8^−^) %leukocyte	GCST90001613	21	21	9
CD28^+^ CD45RA^+^ CD8dim %T cell	GCST90001664	40	40	12
CD28^+^ CD45RA^−^ CD8dim %CD8dim	GCST90001668	30	30	13
CD28^−^ CD127^−^ CD25^++^ CD8br AC	GCST90001675	30	30	17
CD127^−^ CD8br %T cell	GCST90001682	15	15	6
CD127^−^ CD8br %CD8br	GCST90001683	14	14	8
CD127^−^ CD8br AC	GCST90001684	18	18	10
CD45RA^+^ CD28^−^ CD8br AC	GCST90001698	753	753	6
CD24 on IgD^+^ CD38br	GCST90001768	25	25	12
CD25 on CD24^+^ CD27^+^	GCST90001777	28	28	18
CD27 on CD20^−^	GCST90001796	17	17	9
CD27 on IgD^−^ CD38-	GCST90001802	33	33	17
CD34 on HSC	GCST90001870	14	14	7
CD16^−^ CD56 on NK	GCST90001884	31	31	8
CD28 on CD39^+^ activated Treg	GCST90001886	20	20	6
CD16 on CD14^+^ CD16^+^ monocyte	GCST90002005	24	24	10
CD4 on naive CD4^+^	GCST90002024	20	20	12
CD4 on CD39^+^ activated Treg	GCST90002067	25	25	10
CD4 on secreting Treg	GCST90002068	28	28	12
SSC-A on myeloid DC	GCST90002071	21	21	6
SSC-A on CD8br	GCST90002082	19	19	6

IVs = instrumental variables, LD = linkage disequilibrium, SNP = single nucleotide polymorphism.

The results of the 5 MR analysis methods are presented in Table S2 (Supplemental Digital Content, https://links.lww.com/MD/Q784). Table S3 (Supplemental Digital Content, https://links.lww.com/MD/Q785) provides scatter plots for 30 data items. As the IVW method may overlook potential pleiotropic effects, we conducted sensitivity analyses using the MR-Egger and weighted median methods to assess the reliability and consistency of the results. Forward sensitivity analysis showed no heterogeneity in the 30 immune cell phenotypes used for HCC MR analysis (*Q*-test *P* > .05) and no horizontal pleiotropy (MR-Egger intercept method *P* > .05), confirming the credibility of the causal robustness results (Table 2). Both the leave-one-out method and the funnel plots indicate that the data are reliable (Table S3, Supplemental Digital Content, https://links.lww.com/MD/Q785).

**Table 2:** Pleiotropy and heterogeneity analyses for the association of immune cells with HCC.

Immune traits	Panel	Inverse variance weighted		MR-Egger	
		Q	Q_*P*-val	Intercept	*P*-val
IgD^+^ CD24^+^ AC	B cell	11.5963	.4786	−0.0001	.2061
IgD^+^ CD24^−^ AC	B cell	5.5039	.8551	1.35 × 10^−05^	.8014
CD39^+^ resting Treg AC	Treg	2.8403	.7246	4.44 × 10^−05^	.6907
Activated Treg %CD4 Treg	Treg	10.9063	.4512	8.03 × 10^−05^	.2924
CD33br HLA DR^+^ CD14^−^ %CD33br HLA DR^+^	Myeloid cell	4.8176	.9397	−2.11 × 10^−05^	.7132
TD CD8br %T cell	Maturation stages of T cell	5.5602	.5919	−6.47 × 10^−05^	.4876
CD8br AC	TBNK	7.6208	.8674	5.34 × 10^−07^	.9909
CD8dim AC	TBNK	5.8188	.5611	−9.24 × 10^−06^	.8959
CD4^+^ CD8dim %leukocyte	TBNK	12.5863	.4802	−4.96 × 10^−05^	.2060
DN (CD4^−^CD8^−^) %leukocyte	TBNK	4.2705	.8319	3.55 × 10^−06^	.9591
CD28^+^ CD45RA^+^ CD8dim %T cell	Treg	18.1126	.0790	4.45 × 10^−05^	.5116
CD28^+^ CD45RA^−^ CD8dim %CD8dim	Treg	10.6254	.5613	−6.13 × 10^−06^	.9390
CD28^−^ CD127^−^ CD25^++^ CD8br AC	Treg	8.2210	.9420	9.70 × 10^−06^	.8269
CD127^−^ CD8br %T cell	Treg	4.6216	.4638	7.81 × 10^−05^	.5932
CD127^−^ CD8br %CD8br	Treg	5.6304	.5835	9.57 × 10^−05^	.5465
CD127^−^ CD8br AC	Treg	7.6736	.5673	6.37 × 10^−05^	.2376
CD45RA^+^ CD28^−^ CD8br AC	Treg	7.1918	.2068	−2.57 × 10^−05^	.8055
CD24 on IgD^+^ CD38br	B cell	16.8279	.1131	8.57 × 10^−05^	.1324
CD25 on CD24^+^ CD27^+^	B cell	20.5735	.2459	1.61 × 10^−05^	.6121
CD27 on CD20^−^	B cell	2.6878	.9524	−0.0001	.3228
CD27 on IgD^−^ CD38-	B cell	11.0310	.8076	−3.13 × 10^−05^	.3472
CD34 on HSC	Myeloid cell	3.1345	.7918	5.24 × 10^−05^	.3069
CD16^−^ CD56 on NK	TBNK	5.7528	.5689	−2.76 × 10^−05^	.6788
CD28 on CD39^+^ activated Treg	Treg	6.4834	.2620	8.49 × 10^−05^	.2780
CD16 on CD14^+^ CD16^+^ monocyte	Monocyte	8.7347	.4621	4.69 × 10^−05^	.1642
CD4 on naive CD4^+^	Maturation stages of T cell	7.5087	.7565	−0.0001	.2047
CD4 on CD39^+^ activated Treg	Treg	6.8920	.6484	−9.59 × 10^−05^	.1989
CD4 on secreting Treg	Treg	11.7126	.3856	6.25 × 10^−05^	.3237
SSC-A on myeloid DC	cDC	3.3830	.6412	−0.0002	.3459
SSC-A on CD8br	TBNK	1.4927	.9139	0.0001	.6116

30 results, including 12 in the Treg group, 6 each in the TBNK and B-cell groups, 2 each in the T-cell and myeloid maturation groups, and 1 each in the cDC and monocyte groups.

**Figure 3.**
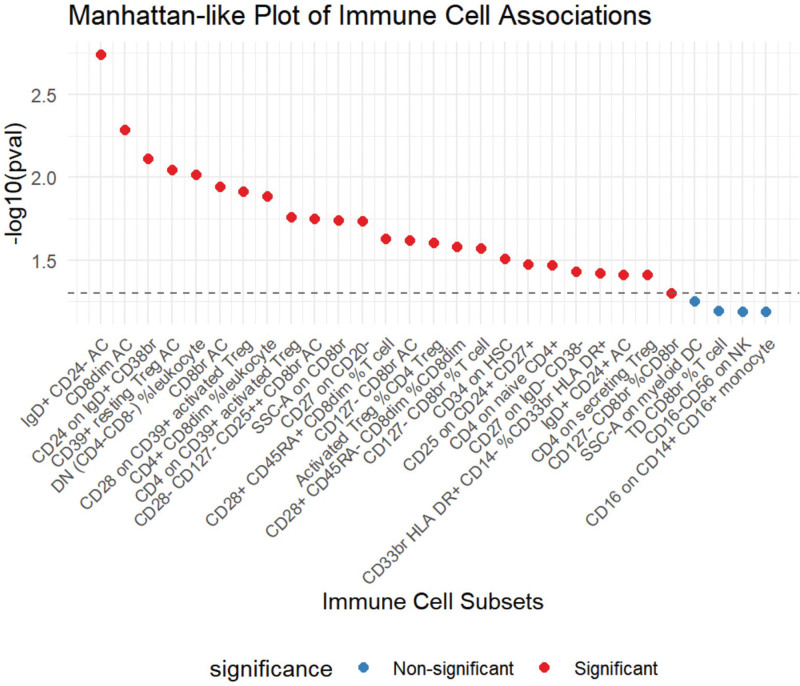
Scatterplot summarizing BWMR results for immune cells causally associated with HCC (*P* < .05 is the significance). BWMR = Bayesian weighted Mendelian randomization, HCC = hepatocellular carcinoma.

**Figure 4.**
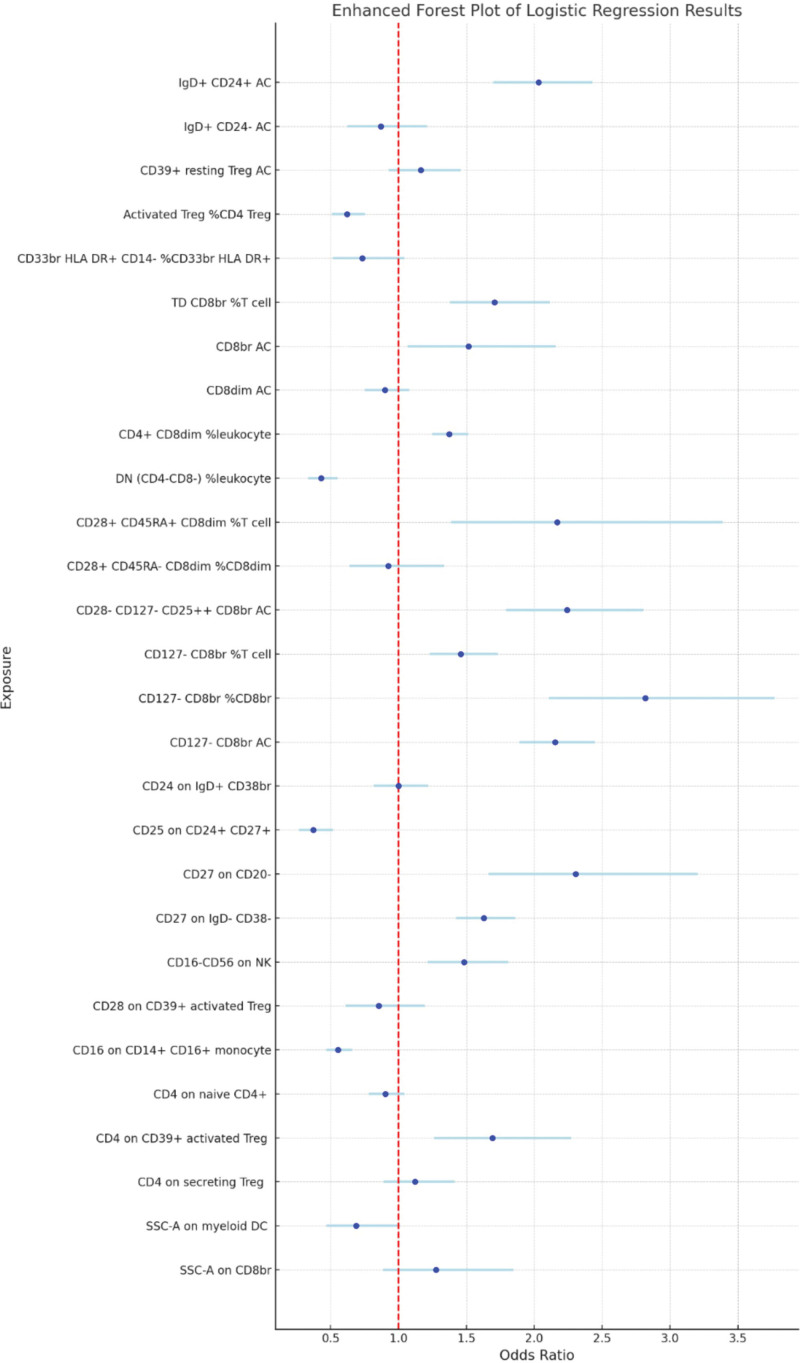
Forest plots summarizing the multifactorial logistic regression analysis results of immune cells with a causal relationship to HCC. HCC = hepatocellular carcinoma.

**Figure 5.**
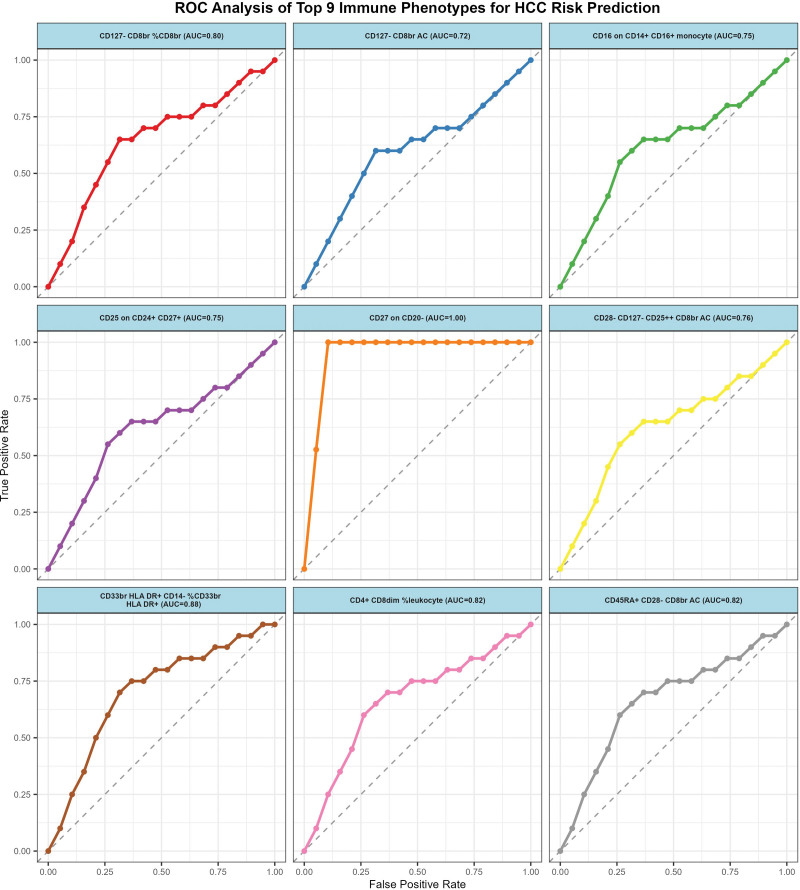
ROC curve summarizing the Random forest results of immune cells with a causal relationship to HCC. The closer the AUC value is to 1, the better the diagnostic performance of the model. High AUC values (excellent diagnostic performance); moderate AUC values (moderate diagnostic performance); low AUC values (poor diagnostic performance. AUC = area under the curve, HCC = hepatocellular carcinoma, ROC = receiver operating characteristic.

**Figure 6.**
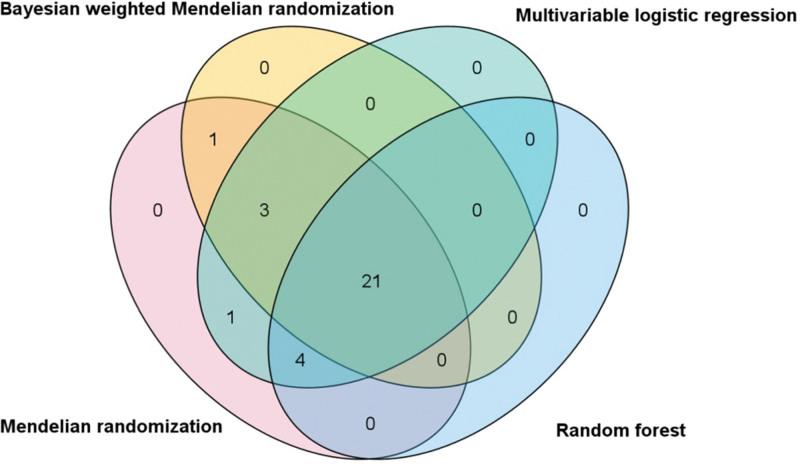
Venn diagram showing the intersection of Mendelian randomization and machine learning for data fetching.

Following the application of the BWMR test, 25 immune cell traits were identified as being associated with HCC (Fig. 3). Subsequently, a multifactorial logistic regression analysis was performed, the results of which are presented in Figure 4 and reveal 28 immune cell characteristics related to HCC. Figure 5 illustrates the evaluation of the 25 immune cells in question using the random forest machine learning method (Fig. 5 shows only the first 9; the remainder can be found in Table S4, Supplemental Digital Content, https://links.lww.com/MD/Q785). In summary, our study identified 21 immune cell characteristics associated with HCC (Fig. 6, results are presented in Table S5, Supplemental Digital Content, https://links.lww.com/MD/Q784), of which 9 are protective factors and 12 are risk factors. Some of the strongest associations were highlighted, including the odds ratio (OR) for CD27 on CD20- related to HCC risk being 1.00035 (95% CI: 1.00008–1.00062, PFDR = 0.038), and the OR for CD33br HLA DR^+^ CD14^−^ %CD33br HLA DR^+^ related to HCC risk being 1.00016 (95% CI: 1.00002–1.00031, PFDR = 0.041), the OR for CD4^+^ CD8dim %leukocyte related to HCC risk being 1.00021 (95% CI: 1.00005–1.00037, PFDR = 0.038), the odds ratio for CD127-CD8br%CD8br related to HCC risk being 1.00031 (95% CI: 1.00000–1.00062, PFDR = 0.048), and the odds ratio for CD28-CD127-CD25^++^ CD8brAC related to HCC risk being 1.00019 (95% CI: 1.00000–1.00037, PFDR = 0.047); the OR for CD25 on CD24^+^ CD27^+^ in relation to HCC risk was 0.99985 (95% CI: 0.99971–1.00000, PFDR = 0.047). Of these 6 immune cells, only one is a protective factor for HCC, while the other 5 are risk factors.

### 3.2. Reverse validation and investigation of the causal impact of HCC onset on immune phenotypes

To verify the causal effect of immune cells on the risk of HCC and further explore its impact on immune cells, we conducted a reverse MR study, using HCC as the exposure and immune cells as the outcome. Using the same instrument selection parameters as in the forward MR, no reverse causal relationship was identified.

### 3.3. Expression and prognostic validation of key immunophenotypes in independent cohorts

**Figure 7.**
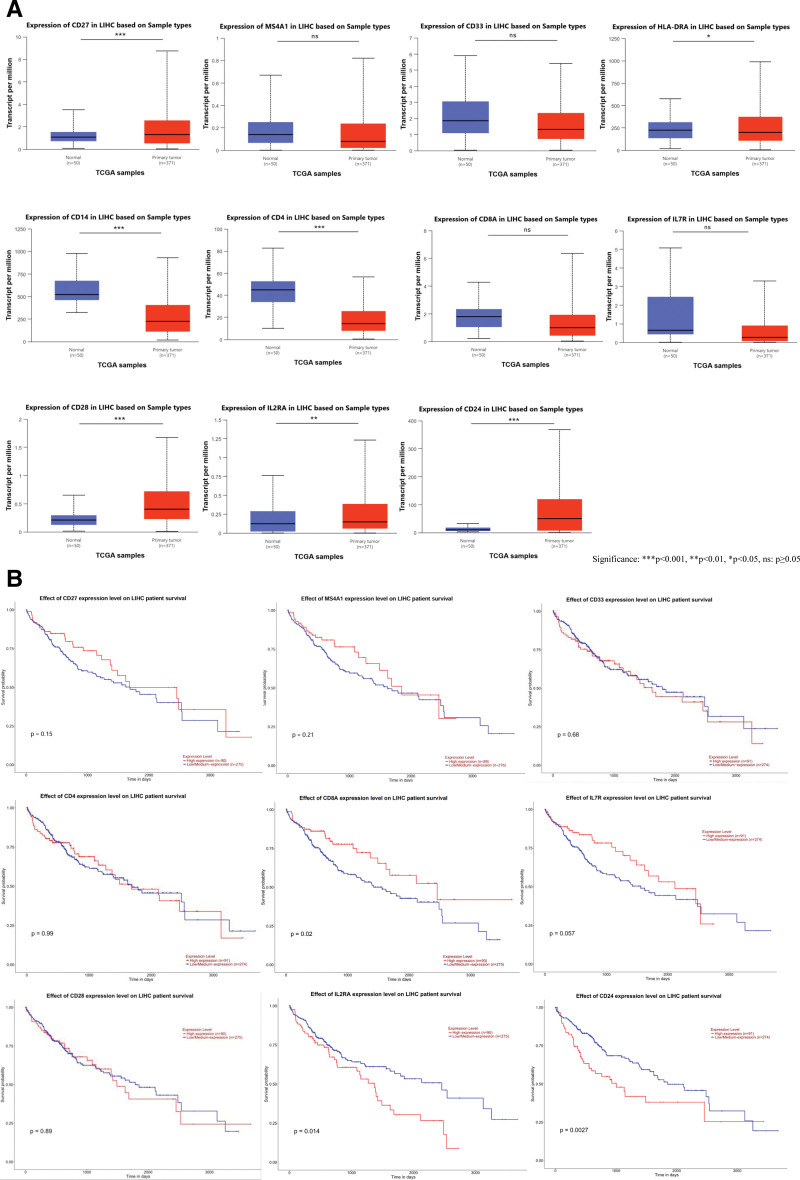
(A) Differential expression analysis of core genes. (B) Survival analysis of core genes.

To validate the MR analysis results, we performed expression analysis (Fig. 7A) and prognostic analysis (Fig. 7B) of core genes for 6 key immune phenotypes in the TCGA-LIHC cohort (Table 3).

**Table 3:** Significant immune phenotypes and their core genes for transcriptome validation.

Immune traits	Panel	Core genes
CD27 on CD20-	B cell	CD27, MS4A1 (CD20)
CD33br HLA DR^+^ CD14^−^ %CD33br HLA DR^+^	Myeloid cell	CD33, HLA-DRA, CD14
CD4^+^ CD8dim %leukocyte	TBNK	CD4, CD8A
CD127^−^ CD8br%CD8br	Treg	IL7R (CD127), CD8A
CD28^−^ CD127^−^ CD25^++^ CD8brAC	Treg	CD28, IL7R, IL2RA (CD25), CD8A
CD25 on CD24^+^ CD27^+^	B cell	IL2RA, CD24, CD27

B cell subset CD27 on CD20^−^: Its core gene CD27 showed significantly upregulated expression in tumor tissues (*P* = 5.58E−05), while MS4A1 (CD20) expression showed no significant difference (*P* = .20). Neither expression level correlated significantly with overall patient survival (*P* = .15; *P* = .21).

CD33br HLA DR^+^ CD14^−^ %CD33br HLA DR^+^ in the myeloid cell group: Core gene CD14 expression was significantly upregulated (*P* = 6.06E−13), HLA-DRA expression was downregulated (*P* = .02), and CD33 expression showed no significant difference. None of these expression levels showed a significant association with overall survival (*P* = .67, *P* = .98, *P* = .68, respectively).

TBNK cell group CD4^+^ CD8dim %leukocyte: CD4 expression was significantly upregulated (*P* = 1.15E−13), while CD8A expression showed no significant difference (*P* = .087). Survival analysis revealed that CD4 expression was not associated with survival (*P* = .99), whereas high CD8A expression was a protective factor (*P* = .02).

In the T cell group, CD127-CD8br%CD8br: IL7R (CD127) and CD8A expression showed no significant differences between tumor and normal tissues (*P* = .50; *P* = .087). High IL7R expression showed a trend toward poor prognosis (*P* = .057), whereas high CD8A expression was a protective factor (*P* = .02).

In the T cell group, CD28-CD127-CD25^++^ CD8brAC: CD28 and IL2RA (CD25) expressions were significantly upregulated (*P* = 1.93E−05; *P* = 2.64E−03), while IL7R expression showed no significant difference. High IL2RA expression correlated with poor prognosis (*P* = .014), while high IL7R expression showed a trend toward poor prognosis (*P* = .057). CD28 expression was unrelated to survival (*P* = .89). CD8A expression in this group was a protective factor (*P* = .02).

An additional protective factor was identified in the B cell subset CD25 on CD24^+^ CD27^+^. The 3 core genes defining this phenotype – CD24, IL2RA, and CD27 – were significantly upregulated in tumor tissues (*P* = 1.62E−12; *P* = 2.64E−03; *P* = 5.58E−05). However, survival analysis revealed that high expression of both CD24 and IL2RA was significantly associated with poor overall survival (*P* = .0027; *P* = .014), whereas CD27 expression showed no significant correlation with survival (*P* = .15).

### 3.4. Exploring potential mechanisms by which key immunophenotypes influence HCC risk

**Figure 8.**
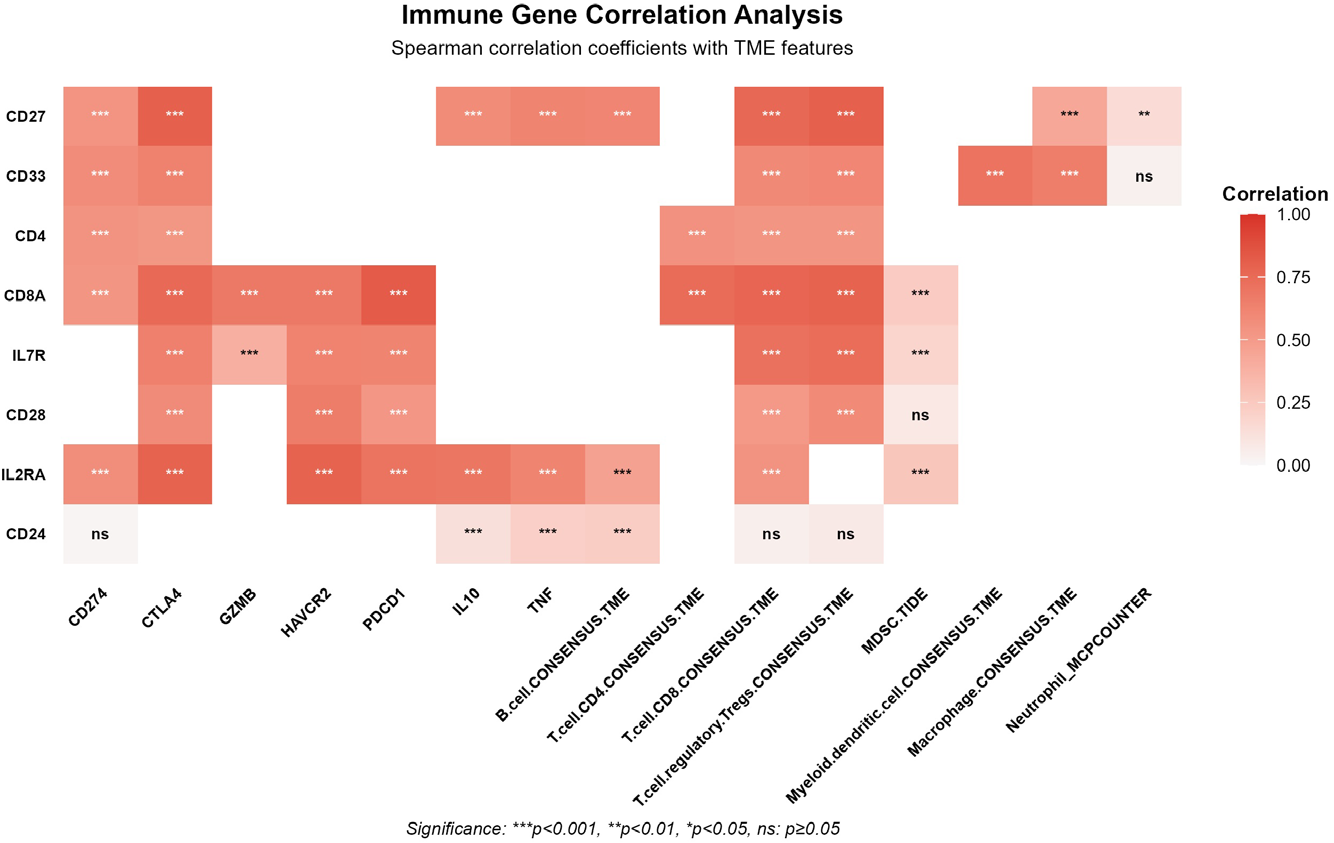
The correlations between core genes and tumor immune microenvironment characteristics.

To further elucidate the underlying immunological mechanisms by which these immune phenotypes influence HCC risk, we conducted an in-depth analysis using the TIMER 3.0 database to examine the correlations between its core genes and tumor immune microenvironment characteristics (Fig. 8).

Analysis of risk-associated immune phenotype genes reveals the following:

B cell phenotype (CD27 on CD20^−^): CD27 expression shows significant positive correlations with immune checkpoint molecules CTLA-4 (rho = 0.804) and PD-L1 (rho = 0.542), CD8^+^ T cells (rho = 0.831), Tregs (rho = 0.802), and M1 macrophage infiltration (Table S6, Supplemental Digital Content, https://links.lww.com/MD/Q786).

Myeloid cell phenotype (CD33br HLA DR^+^ CD14^−^ %CD33br HLA DR^+^): CD33 expression positively correlates with PD-L1 (rho = 0.587), CTLA-4 (rho = 0.631), M2 macrophage infiltration (rho = 0.682), and Treg infiltration. HLA-DRα expression positively correlates with PD-L1 (rho = 0.583) and CTLA-4 (rho = 0.634), whereas CD14 expression negatively correlates with CTLA-4 (rho = −0.145) (Table S6, Supplemental Digital Content, https://links.lww.com/MD/Q786).

T, B, and NK (TBNK) cell phenotype (CD4^+^ CD8dim %leukocyte): Both CD4 and CD8A expression positively correlate with T cell infiltration and immune checkpoint molecules (e.g., CTLA-4, rho = 0.761). High CD8A expression positively correlates with effector T cell subsets, while high CD4 expression negatively correlates with Th1 and Th2 cell subsets (Table S6, Supplemental Digital Content, https://links.lww.com/MD/Q786).

T cell phenotype (CD127-CD8br%CD8br): High IL7R expression strongly correlates with T cell exhaustion markers programmed cell death protein 1 (PD-1) (rho = 0.623) and TIM-3 (HAVCR2, rho = 0.624), as well as with Treg infiltration. CD8A expression positively correlates with CD8^+^ T cell infiltration (rho = 0.995), the cytotoxic molecule granzyme B (GZMB) (rho = 0.676), and PD-1 (rho = 0.823) (Table S6, Supplemental Digital Content, https://links.lww.com/MD/Q786).

T cell phenotype (CD28-CD127-CD25^++^ CD8brAC): Expression of CD28, IL7R, and CD8A all positively correlate with T cell exhaustion features and Treg infiltration. IL2RA shows the strongest correlation with Treg infiltration (rho = 0.637) and the exhaustion marker TIM-3 (rho = 0.787) (Table S6, Supplemental Digital Content, https://links.lww.com/MD/Q786).

Analysis of the protective phenotype (CD25 on CD24^+^ CD27^+^) reveals that CD24, IL2RA, and CD27 positively correlate with B cell infiltration. IL2RA and CD27 also positively correlate with CD8^+^ T cells, Tregs, and immune checkpoint molecules, whereas CD24 exhibits a negative correlation with CD8^+^ T cell infiltration. Additionally, IL2RA strongly positively correlates with the anti-inflammatory factor IL10 expression (rho = 0.696) (Table S6, Supplemental Digital Content, https://links.lww.com/MD/Q786).

## 4. Discussion

This study employs a MR framework to reveal the causal role of immune cells in HCC development. By conducting multidimensional validation of key immune phenotypes, our analysis translates genetic discoveries to provide mechanistic insights regarding the tumor immune microenvironment. Our findings indicate that HCC risk is not determined by the mere presence or absence of specific immune cells, but is profoundly regulated by their functional states and subset-specific properties. This suggests that within the liver’s unique immune microenvironment, disruption of the intricate network of cellular interactions and signaling pathways that maintain immune homeostasis may drive tumorigenesis.

### 4.1. The complex role of B cell subpopulations: from risk phenotypes to protective phenotypes

Our analysis of B cell phenotypes reveals unprecedented complexity in HCC. Specifically, analysis of the risk phenotype characterized by CD27 on CD20^−^ indicates that high CD27 expression itself does not directly cause poor prognosis. Instead, it serves as a marker of a key immunoregulatory node within a microenvironmental state characterized by highly immune-infiltrated yet strongly immunosuppressed conditions. Its strong positive correlations with CTLA-4, PD-L1, Tregs, and CD8^+^ T cells suggest that the CD27 on CD20^−^ B cell subset may drive tumor progression by promoting T cell exhaustion or directly exerting immunosuppressive functions.^[26,27]^ This challenges the traditional view of simplistically categorizing B cells into either antitumor or pro-tumor populations, emphasizing the importance of subpopulation classification based on more refined surface markers such as CD20 deficiency.^[28]^

More revealing is the discovery of the protective phenotype CD25 on CD24^+^ CD27^+^. This phenotype presents a core contradiction: each of its individual core genes (CD24, IL2RA) is independently assessed as a clear risk factor, yet their combination confers protective effects. This seemingly paradoxical phenomenon precisely reveals the high specificity of cell subset functions. Microenvironment analysis suggests that the co-expression of these 3 markers likely defines a distinct regulatory B cell (Breg) subset.^[29]^ Its protective mechanism may lie in the following: under active humoral immune responses (marked by CD27 and CD24), this subset secretes immunoregulatory factors through high expression of IL2RA, and potentially, in association with high IL-10 expression.^[30]^ This enables it to effectively coordinate and maintain immune equilibrium, enhancing antitumor efficacy while suppressing pathological inflammation or excessive T cell exhaustion.^[31]^ This opens new perspectives for exploring the positive role of B cells in cancer immune surveillance. Notably, in the liver – an organ rich in B cells that requires maintenance of immune tolerance – such regulatory B cell subsets may play a crucial “stabilizing” role in preventing excessive inflammatory responses and suppressing spontaneous tumorigenesis.^[32]^

### 4.2. Myeloid-derived suppressive microenvironment drives HCC risk

The validated phenotype of myeloid cells – CD33br HLA DR^+^ CD14^−^ %CD33br HLA DR^+^ – clearly delineates a pathway that promotes HCC risk by shaping an inhibitory microenvironment. The core features of this phenotype – high expression of CD33 (an inhibitory receptor), downregulated HLA-DRα/β (antigen presentation), and lack of CD14 expression – closely align with the characteristics of myeloid-derived suppressor cells and immunosuppressive dendritic cell subsets.^[33]^ Its risk effect stems from the suppressive microenvironment collectively shaped by this population. This environment is characterized by M2 macrophages and Treg infiltration, alongside upregulation of immune checkpoint molecules, and accompanied by impaired antigen presentation.^[34]^ The negative correlation between CD14 and CTLA-4 further indicates that this phenotype lacks a subset of functionally distinct CD14^+^ monocytes/macrophages, thereby defining a more “pure” pro-tumor myeloid population.^[35]^ Given the presence of numerous resident myeloid cells (e.g., Kupffer cells) in the liver, this pro-tumor subpopulation may interact with these resident cells to jointly shape a local environment conducive to tumor growth.

### 4.3. T cell dysfunction is central to the genetic risk of HCC

Our research provides compelling evidence that T cell dysfunction is a central component of genetic susceptibility to HCC.^[36]^ This finding is particularly significant within the context of liver immunity. To cope with persistent antigenic stimulation, the liver has evolved a microenvironment rich in immune regulatory mechanisms, such as Tregs and immune checkpoint molecules, which are designed to prevent autoimmunity.^[37]^ Our findings suggest that hepatocellular carcinoma cells likely “hijack” this innate liver tolerance, rendering infiltrating T cells more susceptible to functional exhaustion and thereby evading immune surveillance. Multiple risk phenotypes precisely point to distinct states of exhaustion in CD8^+^ T cells.^[16]^ The CD127^−^ CD8br phenotype directly identifies an IL-7 receptor-deficient CD8^+^ T cell subset, where IL7R deficiency is a key marker for T cell loss of long-term survival capacity and entry into exhaustion.^[38]^ Despite belonging to the cytotoxic T cell lineage, this population exhibits functional dysfunction. The CD28^−^ CD127^−^ CD25^++^ CD8br AC phenotype characterizes a severely dysfunctional CD8^+^ T cell or CD8^+^ Treg-like cell subset. Here, the high expression of IL2RA serves as a potent inhibitory driver signal. The absence of both CD28 (co-stimulatory signal) and CD127 (survival signal) collectively drives this subset into profound functional exhaustion and may also confer suppressive properties.^[39,40]^ Together, these factors completely negate any potential anti-tumor immunological benefits that CD8A might otherwise provide.

Of particular importance is the analysis of the CD4^+^ CD8dim %leukocyte population, which reveals a critical paradox: while CD8A itself is a protective factor, the CD4^+^ CD8dim %leukocyte population – defined by the combined expression levels of CD4 and CD8 – becomes a risk factor. This is likely because this combination characterizes a dysfunctional or ineffective T cell state. Specifically, relatively high CD4 expression (potentially representing pro-inflammatory helper subsets or inefficient tumor infiltration) together with relatively low CD8A expression (indicating impaired cytotoxic function) collectively diminishes the anti-tumor immune activity of CD8^+^ T cells.^[41,42]^ Therefore, this highlights the decisive influence of interactions within T cell subsets on overall function.

### 4.4. Comparison with previous Mendelian randomization studies and positioning of this study

Although studies by Tang et al^[12]^ studying the European population, and Nov et al^[13]^ focusing on the Japanese population, have explored the association between immune cells and HCC using MR, this study differs from theirs in key aspects. Compared with Tang’s study, we did not identify any overlapping significant immune phenotypes. This discrepancy may result from our use of more rigorous Bayesian-weighted MR and machine learning cross-validation methods. These approaches allowed us to identify novel, more reliable features. This finding further highlights the highly complex immunogenetic etiology of HCC.

The core contribution of this study lies in moving beyond mere association identification. Using multi-omics validation with TCGA data, we have, for the first time, linked genetic signals to specific tumor immune microenvironment features – such as regulatory T cell (Treg) infiltration – and patient prognosis. This provides new, experimentally testable mechanistic insights into the immunopathogenesis of HCC. At the same time, the substantial divergence compared to the findings of Nov et al^[13]^ strongly suggests ethnic heterogeneity in causal relationships. Therefore, our work not only identifies promising candidate targets but also highlights the imperative for cross-ethnic collaborative analyses and in-depth mechanistic investigations in this field.

## 5. Research significance and limitations

The significance of this study lies in translating the statistical associations identified by MR into biologically meaningful mechanistic hypotheses. These validated immune features provide novel candidate targets for HCC immune subtyping, prognostic assessment, and combined immunotherapy strategies. Nevertheless, this study also has several limitations. First, the primary genetic data, including immune cell phenotypes and HCC GWAS data, were derived from populations of European ancestry. The generalizability of our findings requires further validation in independent genetic cohorts from other ethnic groups, particularly Asian populations with high HCC incidence. Second, MR analysis inherently relies on 3 core assumptions. Despite extensive sensitivity analyses, we cannot entirely rule out potential bias due to horizontal pleiotropy. Third, the immune cell phenotype data we utilized were derived from blood samples. These may differ from the specific phenotypes and functions of immune cells within the local liver tissue. Finally, our exploration of mechanisms primarily relied on bioinformatics analyses of public databases. These valuable findings, particularly the inferences regarding specific protective B cell subsets, require experimental validation through future studies. Examples include flow cytometry sorting and in vitro functional assays.

## 6. Conclusions and outlook

In summary, this study combines causal inference with biological validation to clearly reveal the pivotal role of specific immune cell functional subpopulations in HCC development. Our findings underscore that future immune intervention strategies must transcend mere enhancement of immune cell infiltration and instead focus on precisely modulating the functional states of specific immune subpopulations, such as reversing CD8^+^ T cell exhaustion, targeting suppressive myeloid populations, or strategically harnessing B cell subpopulations with protective regulatory functions. The key immune phenotypes and their core genes identified in this study provide crucial theoretical foundations and candidate targets. These findings support the development of novel risk prediction biomarkers and precision immunotherapy targets.

## Acknowledgments

We extend our gratitude to the IEU Open GWAS database, UALCAN Database and TIMER 3.0 Database for providing the necessary data for this study, and thanks to the developers of R software and R packages for their contributions and convenience.

## Author contributions

**Conceptualization**: YunQi Hua.

**Data curation**: BaoChun Wang.

**Formal analysis**: Ge Song.

**Investigation**: YuBo Liu.

**Methodology**: XiaoLing Tian.

**Project administration**: YunQi Hua.

**Resources**: XiaoLing Tian, YunQi Hua.

**Software**: XiaoLing Tian.

**Supervision**: YuQian Gao, JinYan Wang.

**Validation**: XinYi Zhang.

**Writing – original draft**: XiaoLing Tian.

**Writing – review & editing**: XiaoLing Tian, YunQi Hua.

## Supplementary Material
