# Supplementary material for: Causal relationship of immune cell characteristics in hepatocellular carcinoma: A multi-omics analysis based on Mendelian randomization
Source: Medicine (Baltimore). 2026 May 12;104(49):e45942. doi: 10.1097/MD.0000000000045942 (PMC12689069; doi:10.1097/MD.0000000000045942)
Supplement: Supplementary file 2 [file medi-104-e45942-s002.pdf]

Supplementary table3

S3_Table_1 Results of MR leave−one−out sensitivity analysis.

S3_Table_2 Scatter plots of MR sensitivity analysis.

S3_Table_3 Funnel plots of MR sensitivity analysis.

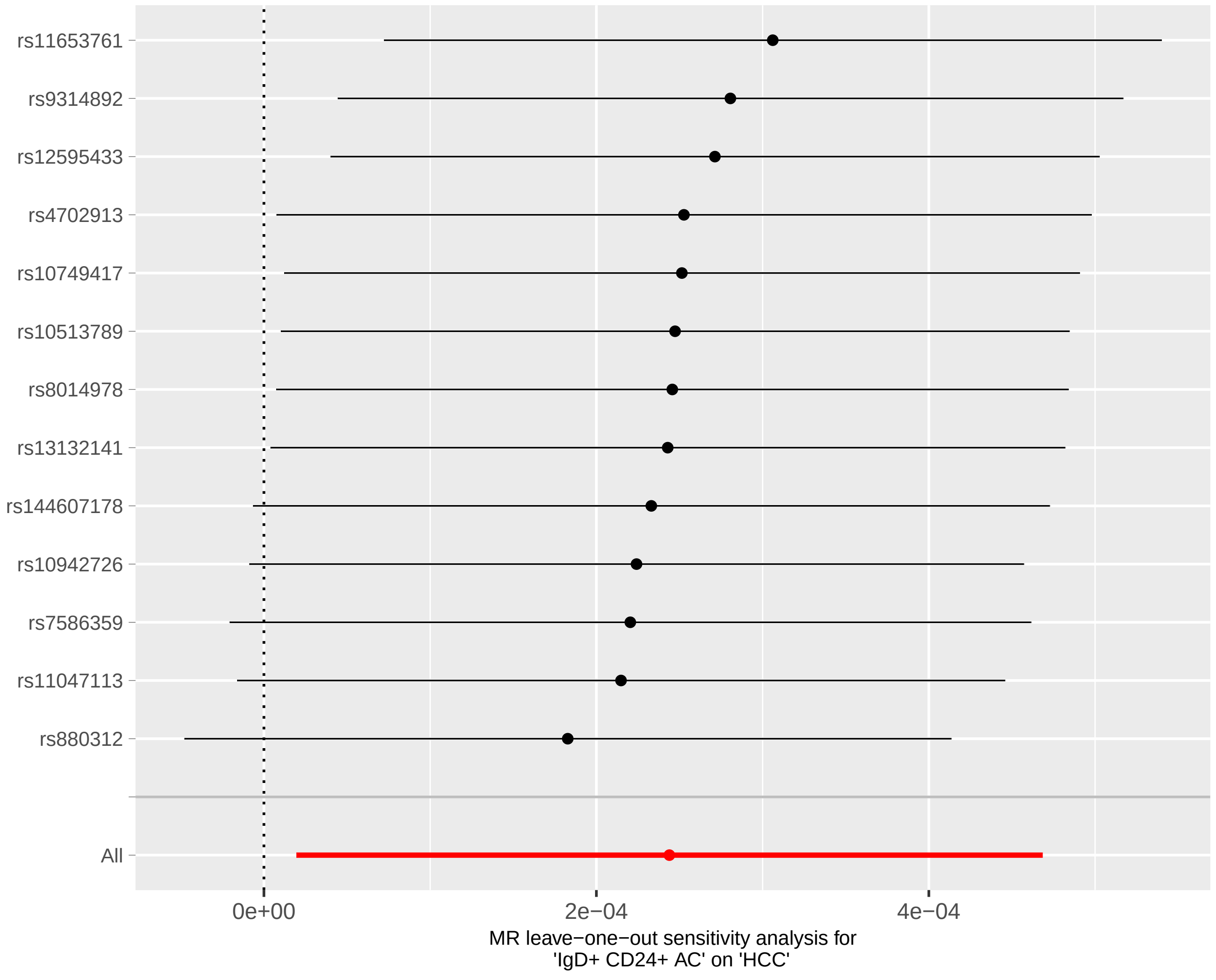

rs11653761
rs9314892
rs12595433
rs4702913
rs10749417
rs10513789
rs8014978
rs13132141
rs144607178
rs10942726
rs7586359
rs11047113
rs880312
All
0e+00
2e−04
4e−04
MR leave−one−out sensitivity analysis for
'IgD+ CD24+ AC' on 'HCC'

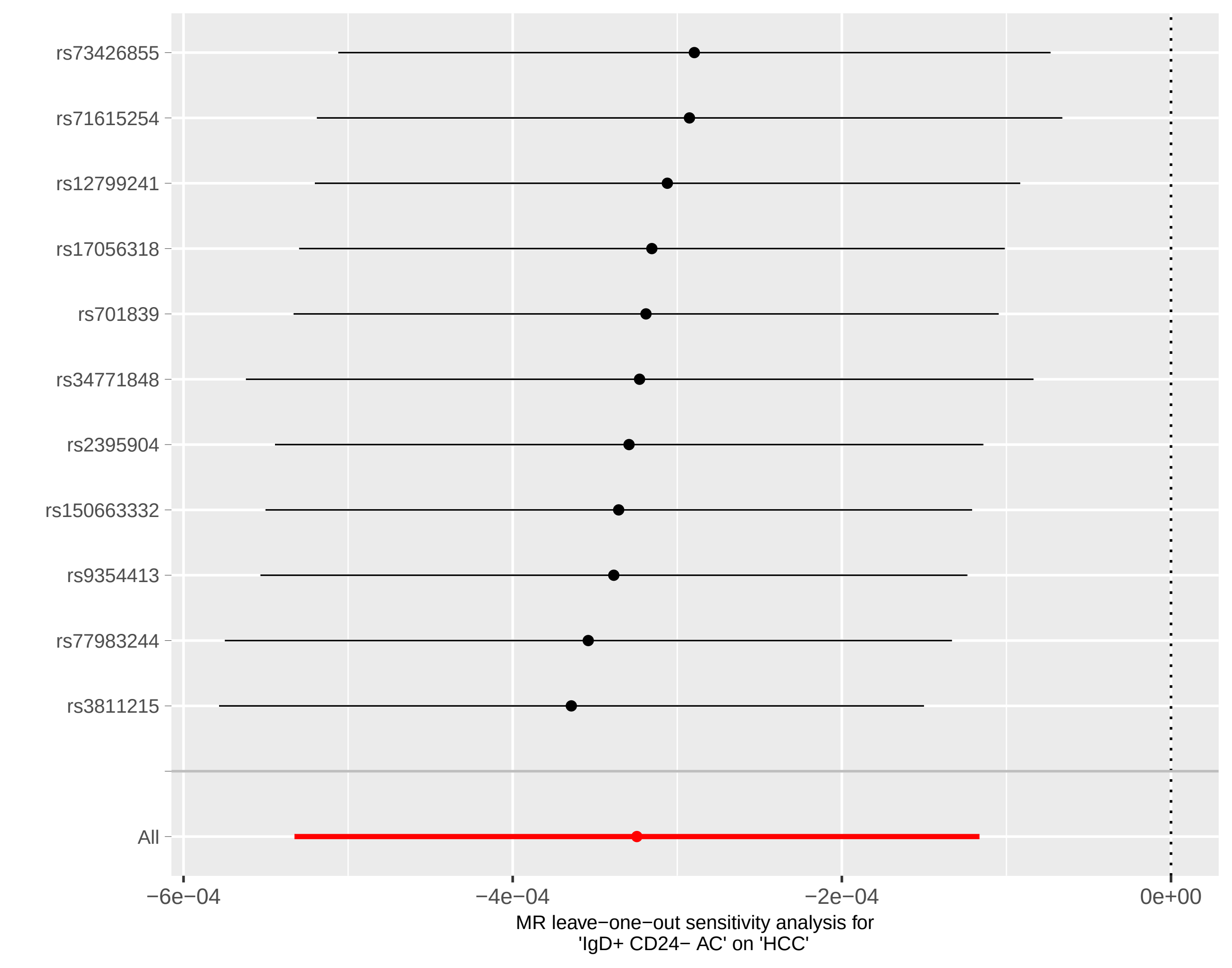
rs73426855
rs71615254
rs12799241
rs17056318
rs701839
rs34771848
rs2395904
rs150663332
rs9354413
rs77983244
rs3811215
All
−6e−04
−4e−04
−2e−04
0e+00
MR leave−one−out sensitivity analysis for
'IgD+ CD24− AC' on 'HCC'

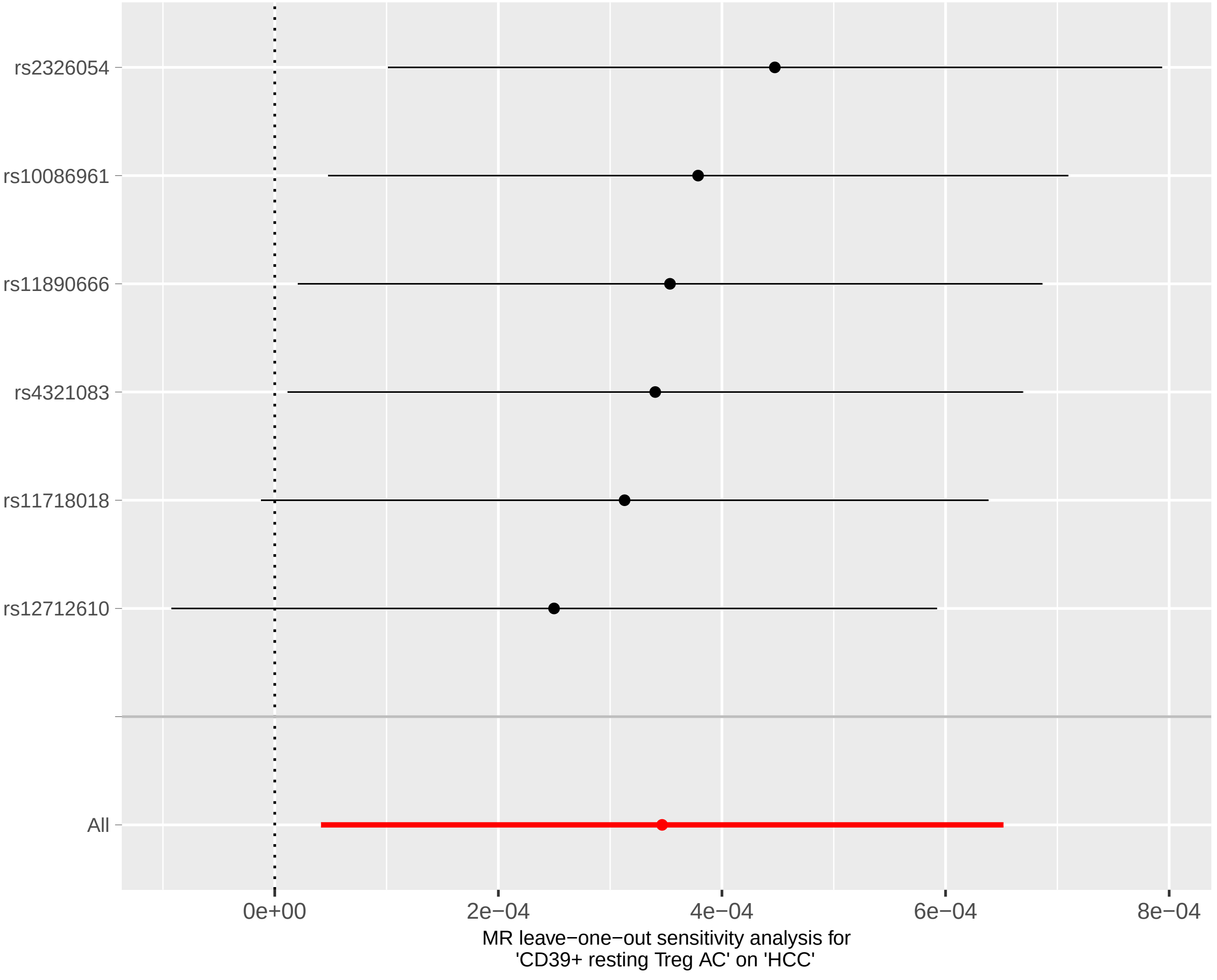
rs2326054
rs10086961
rs11890666
rs4321083
rs11718018
rs12712610
All
0e+00
2e−04
4e−04
6e−04
8e−04
MR leave−one−out sensitivity analysis for
'CD39+ resting Treg AC' on 'HCC'

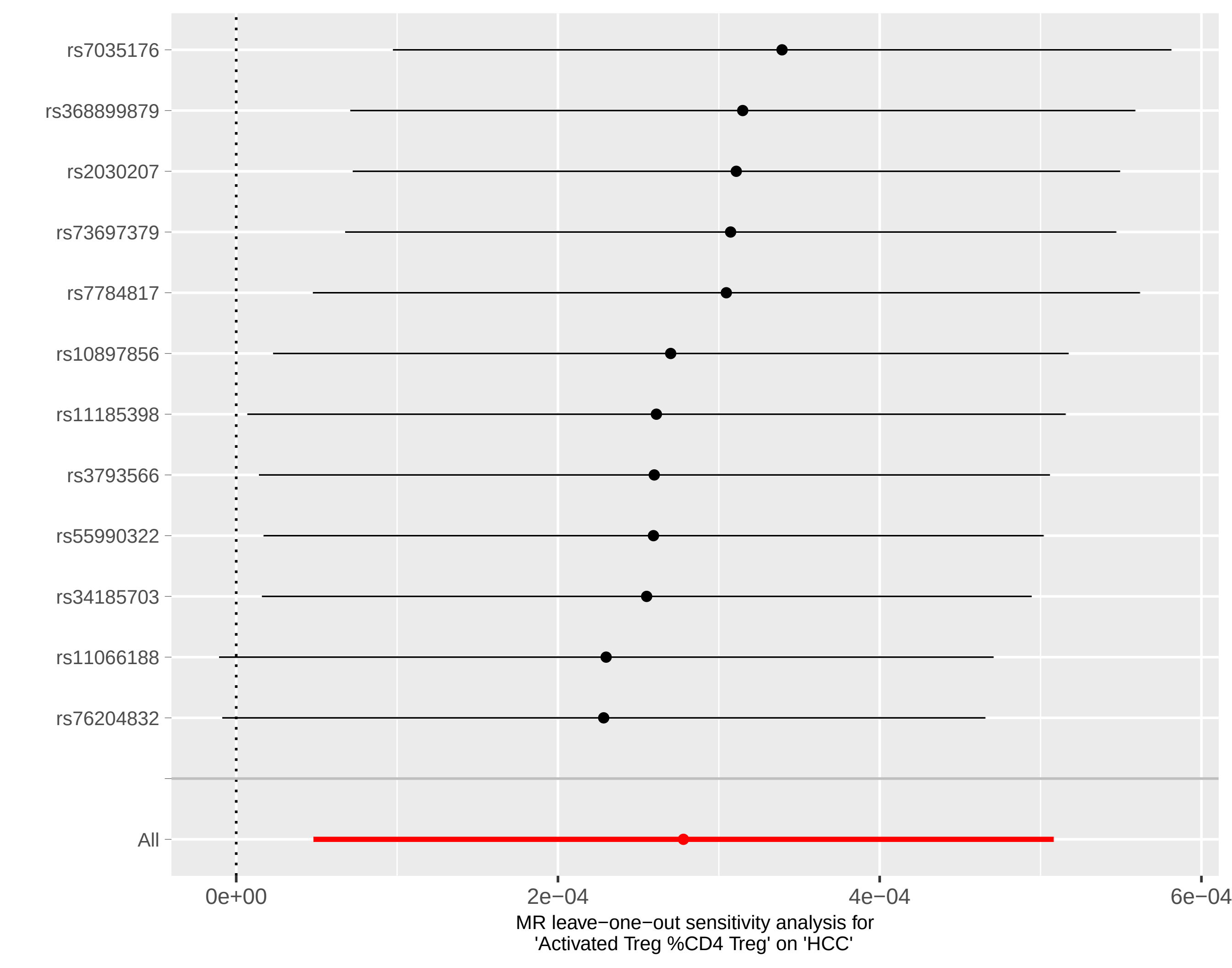
rs7035176
rs368899879
rs2030207
rs73697379
rs7784817
rs10897856
rs11185398
rs3793566
rs55990322
rs34185703
rs11066188
rs76204832
All
0e+00
2e−04
4e−04
6e−04
MR leave−one−out sensitivity analysis for
'Activated Treg %CD4 Treg' on 'HCC'

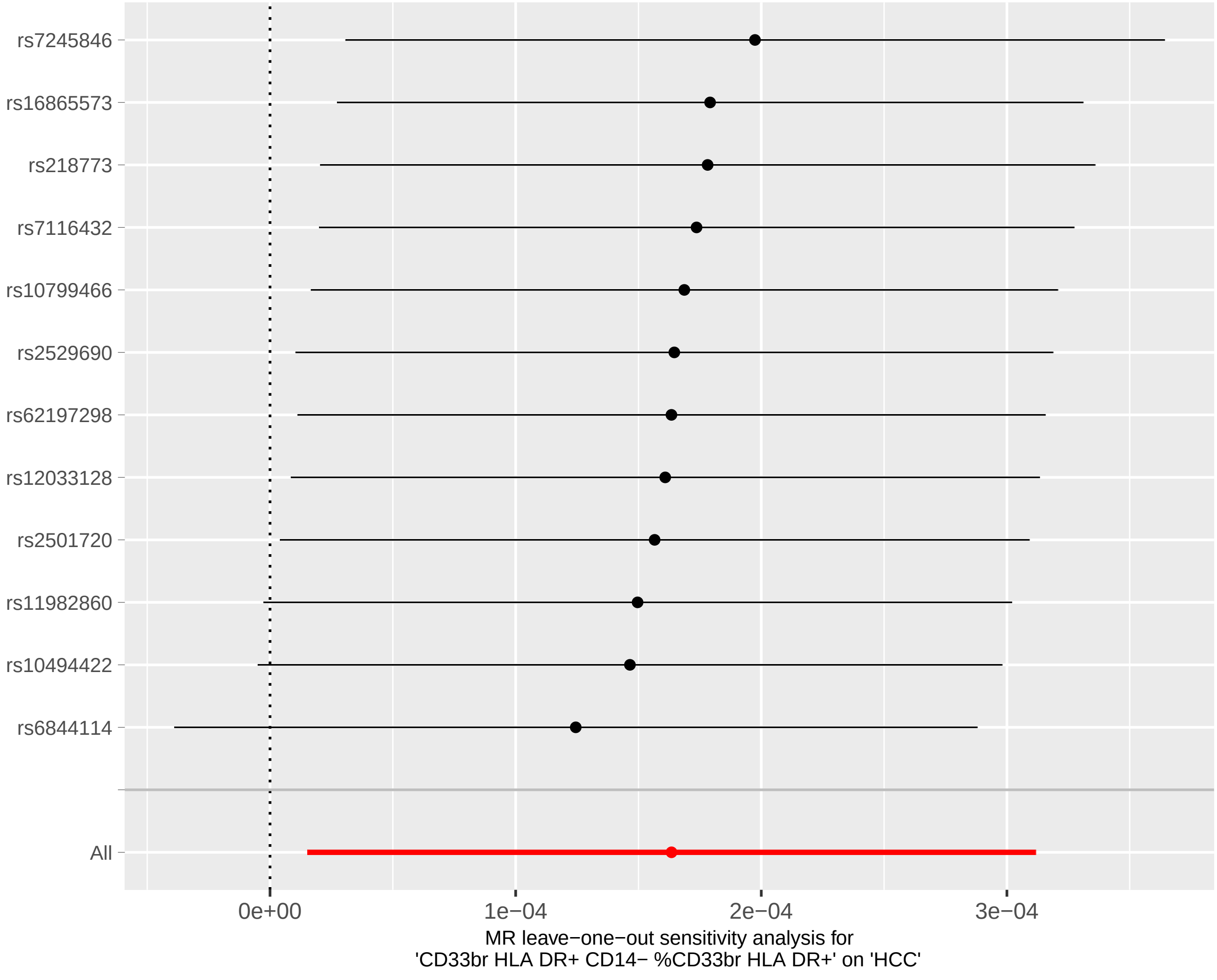
rs7245846
rs16865573
rs218773
rs7116432
rs10799466
rs2529690
rs62197298
rs12033128
rs2501720
rs11982860
rs10494422
rs6844114
All
0e+00
1e−04
2e−04
3e−04
MR leave−one−out sensitivity analysis for
'CD33br HLA DR+ CD14− %CD33br HLA DR+' on 'HCC'

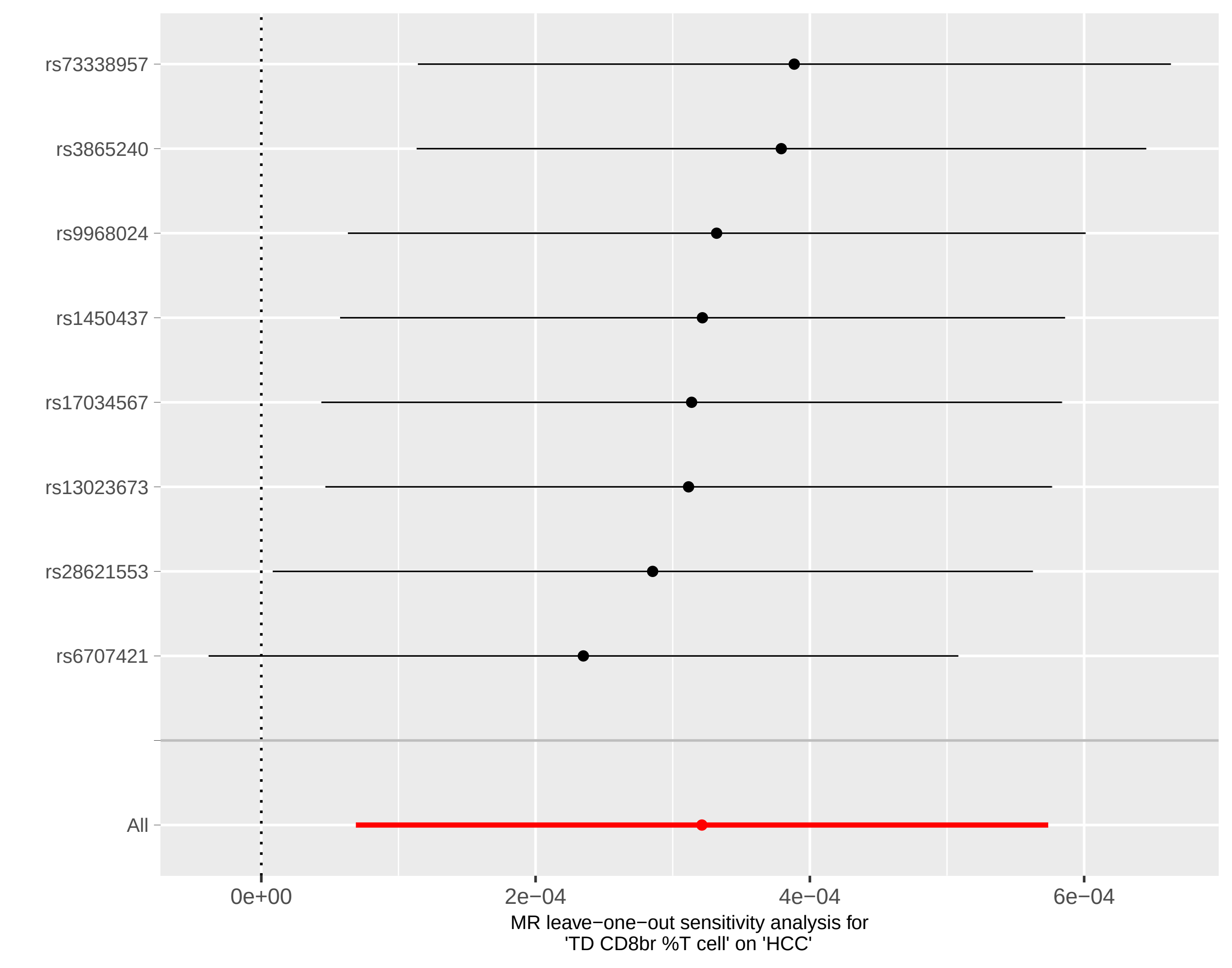
rs73338957
rs3865240
rs9968024
rs1450437
rs17034567
rs13023673
rs28621553
rs6707421
All
0e+00
2e−04
4e−04
6e−04
MR leave−one−out sensitivity analysis for
'TD CD8br %T cell' on 'HCC'

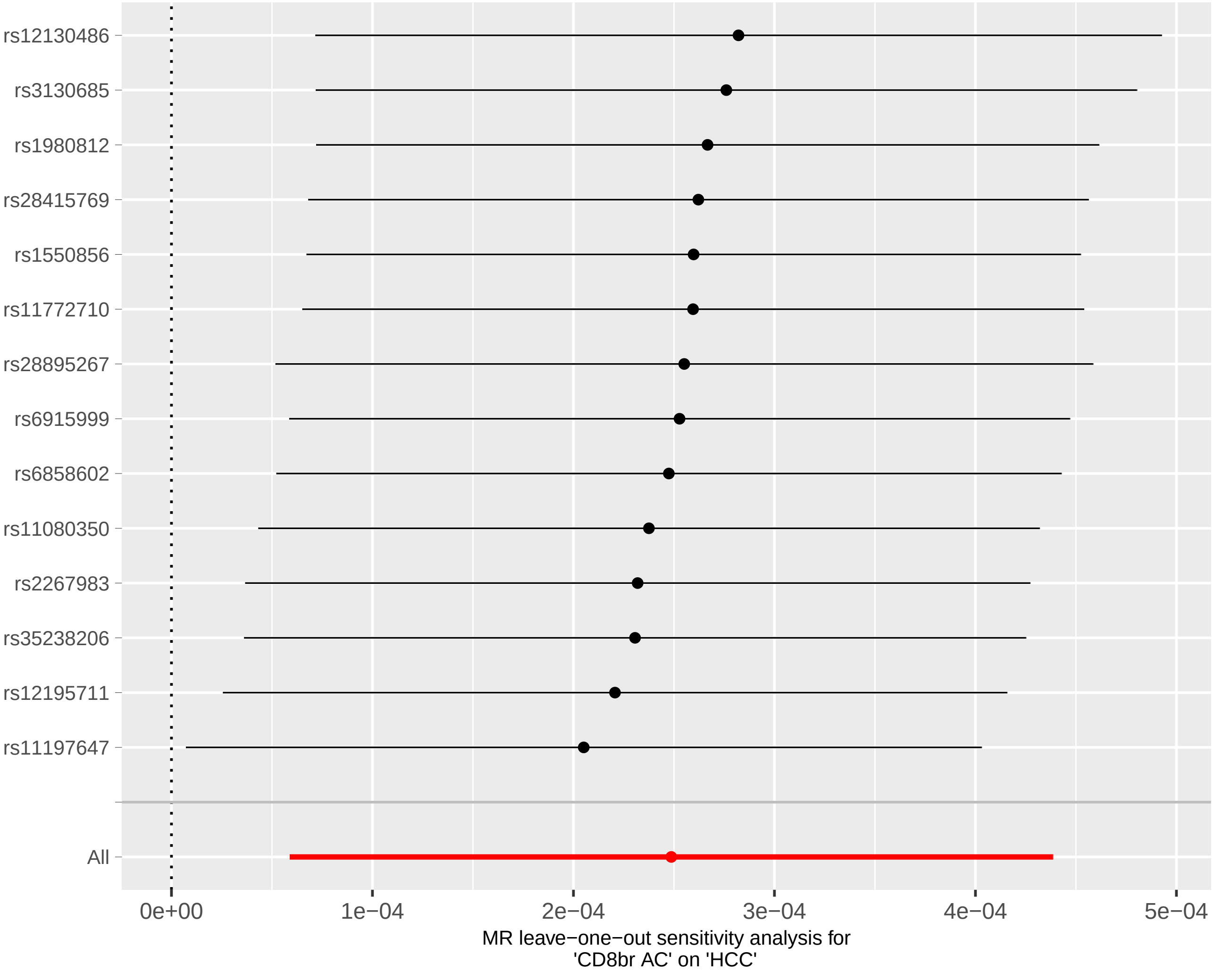
rs12130486
rs3130685
rs1980812
rs28415769
rs1550856
rs11772710
rs28895267
rs6915999
rs6858602
rs11080350
rs2267983
rs35238206
rs12195711
rs11197647
All
0e+00
1e−04
2e−04
3e−04
4e−04
5e−04
MR leave−one−out sensitivity analysis for
'CD8br AC' on 'HCC'

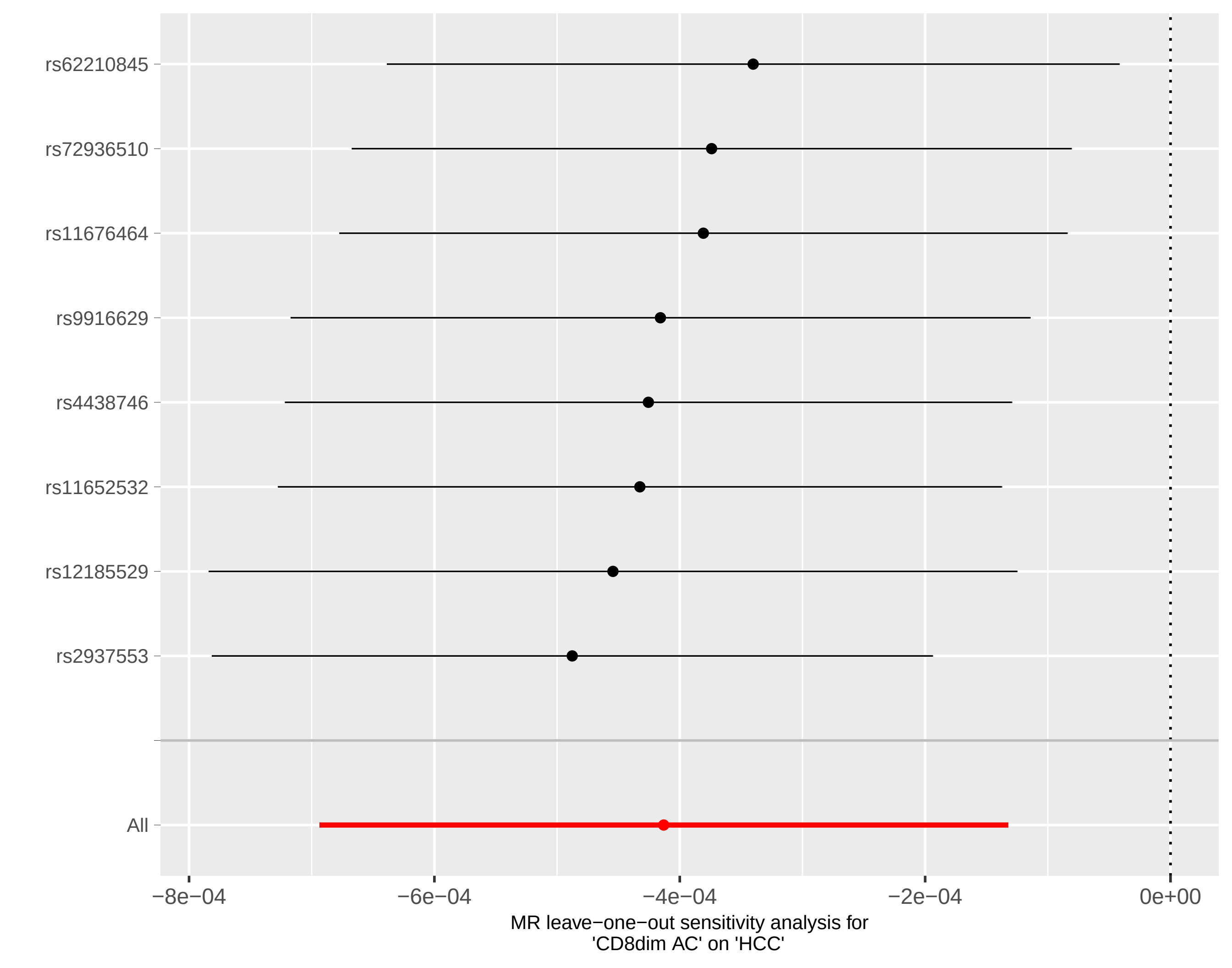

rs62210845
rs72936510
rs11676464
rs9916629
rs4438746
rs11652532
rs12185529
rs2937553
All
−8e−04
−6e−04
−4e−04
−2e−04
0e+00
MR leave−one−out sensitivity analysis for
'CD8dim AC' on 'HCC'

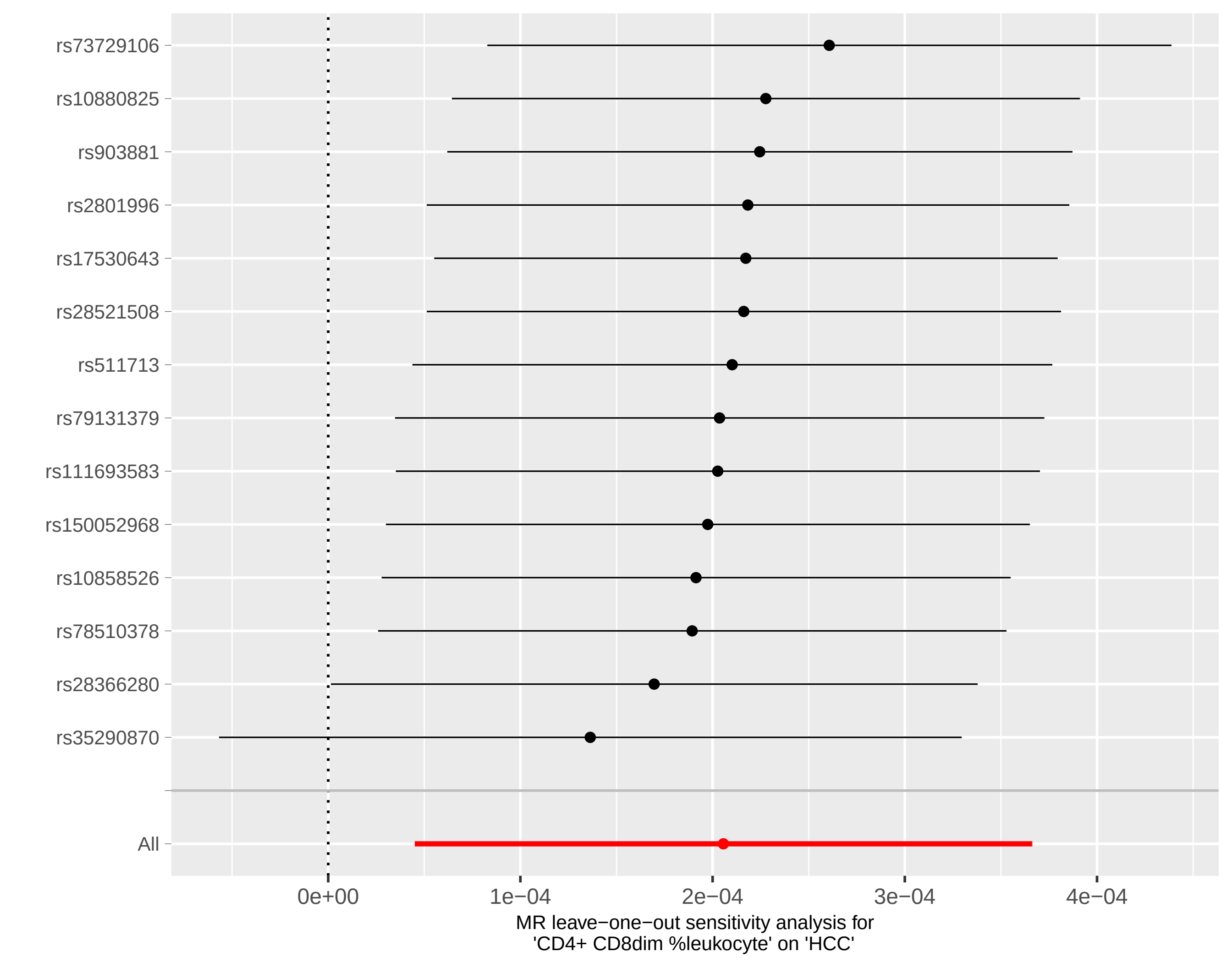
rs73729106
rs10880825
rs903881
rs2801996
rs17530643
rs28521508
rs511713
rs79131379
rs111693583
rs150052968
rs10858526
rs78510378
rs28366280
rs35290870
All
0e+00
1e−04
2e−04
3e−04
4e−04
MR leave−one−out sensitivity analysis for
'CD4+ CD8dim %leukocyte' on 'HCC'

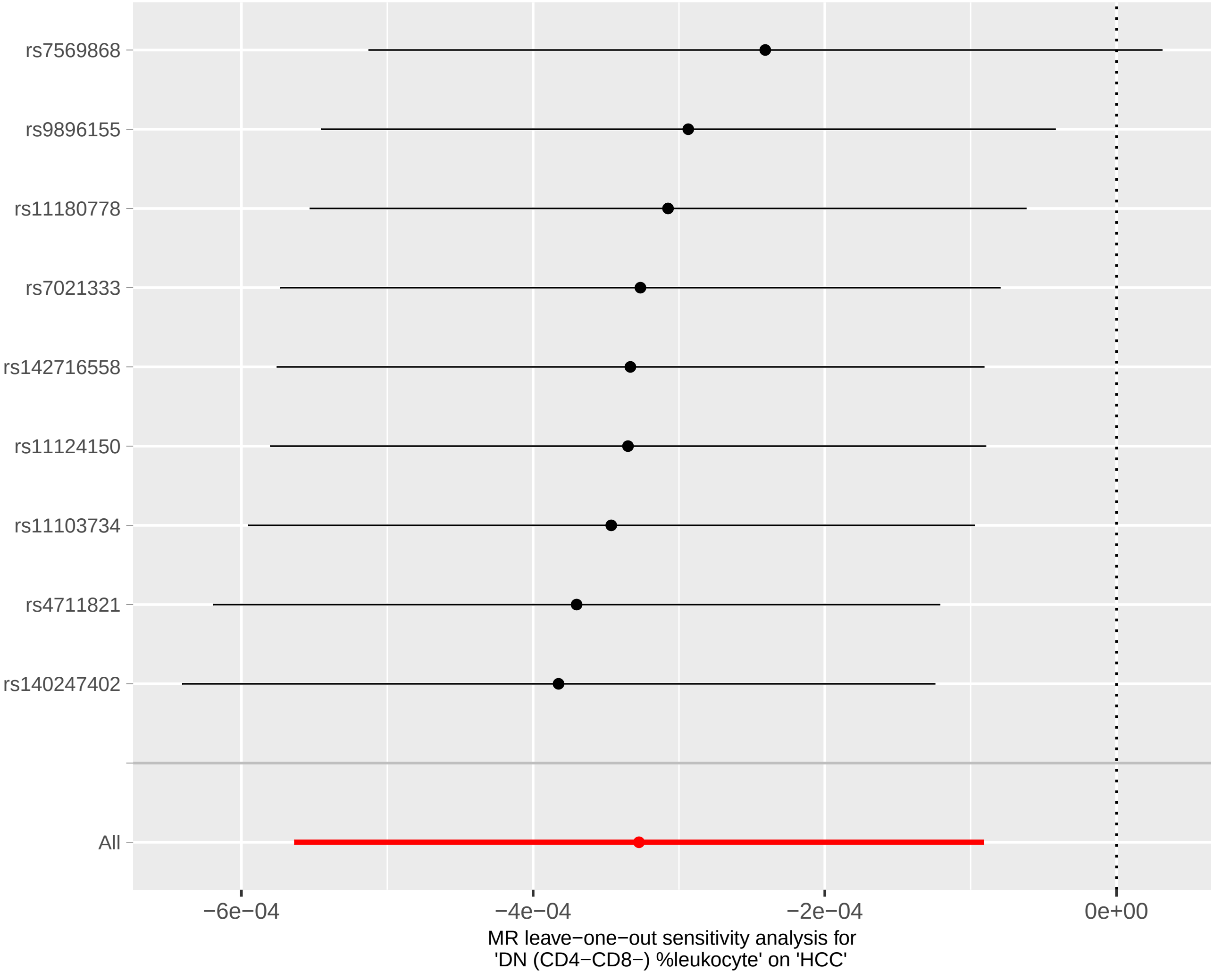

rs7569868
rs9896155
rs11180778
rs7021333
rs142716558
rs11124150
rs11103734
rs4711821
rs140247402
All
−6e−04
−4e−04
−2e−04
0e+00
MR leave−one−out sensitivity analysis for
'DN (CD4−CD8−) %leukocyte' on 'HCC'

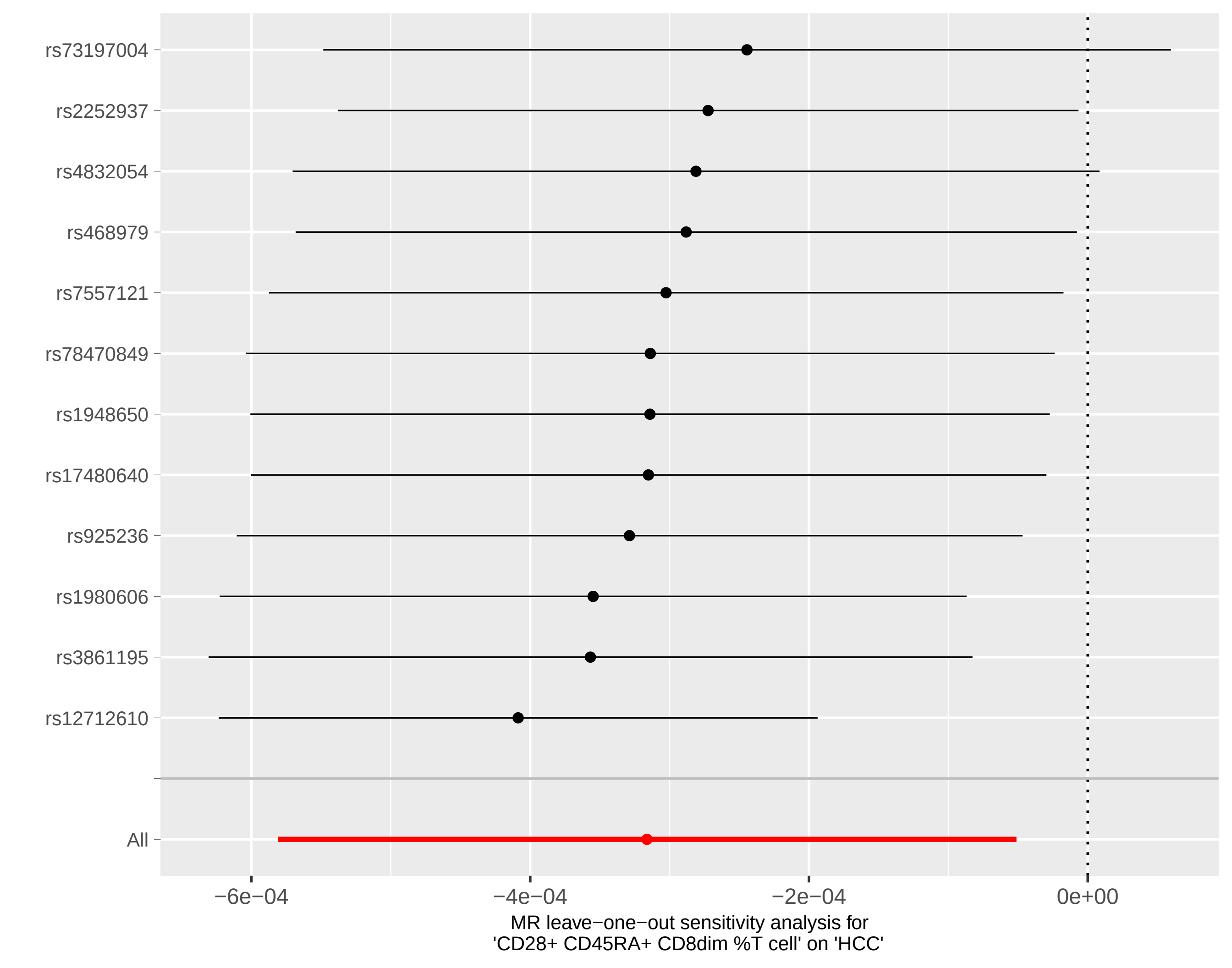
rs73197004
rs2252937
rs4832054
rs468979
rs7557121
rs78470849
rs1948650
rs17480640
rs925236
rs1980606
rs3861195
rs12712610
All
−6e−04
−4e−04
−2e−04
0e+00
MR leave−one−out sensitivity analysis for
'CD28+ CD45RA+ CD8dim %T cell' on 'HCC'

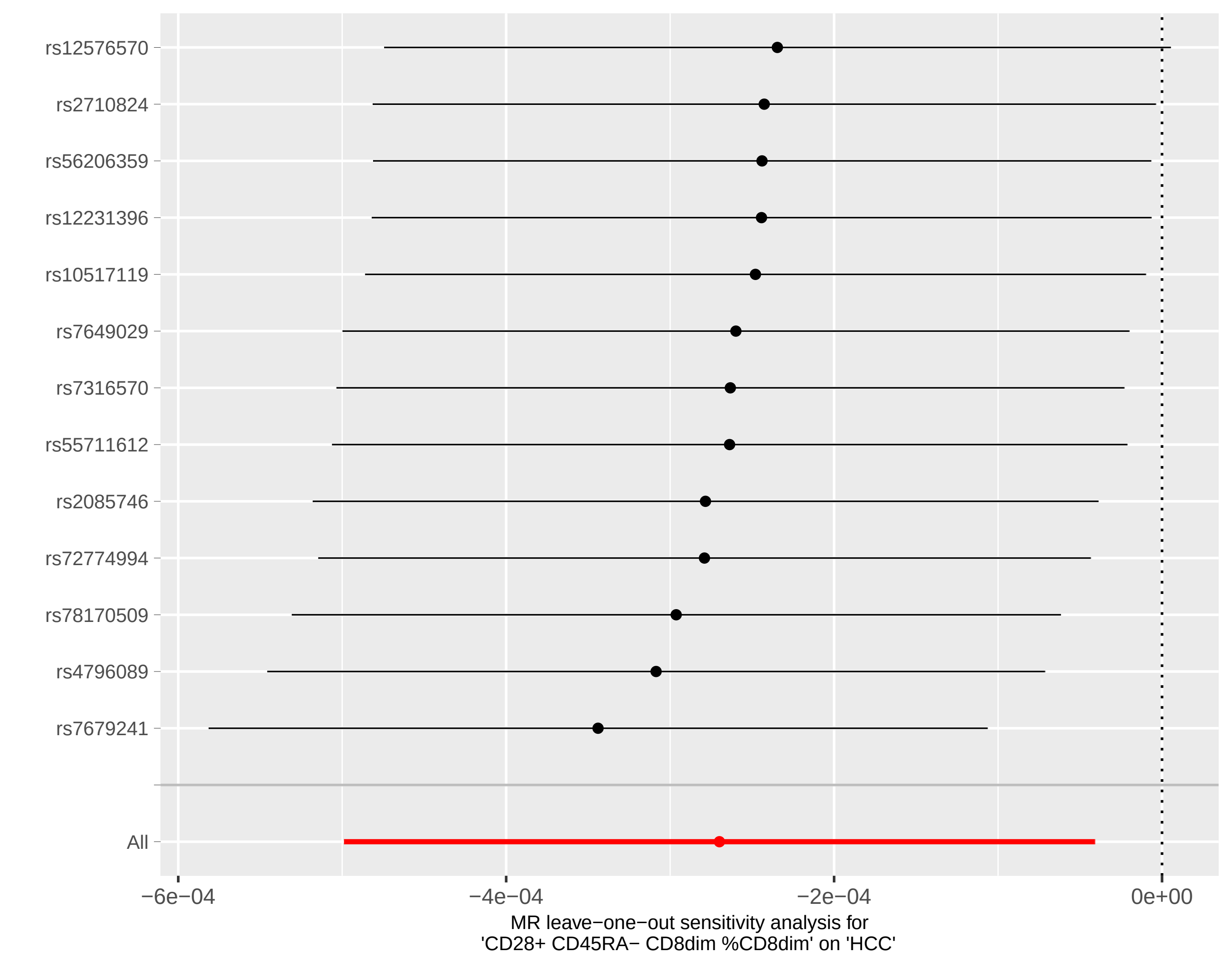
rs12576570
rs2710824
rs56206359
rs12231396
rs10517119
rs7649029
rs7316570
rs55711612
rs2085746
rs72774994
rs78170509
rs4796089
rs7679241
All
−6e−04
−4e−04
−2e−04
0e+00
MR leave−one−out sensitivity analysis for
'CD28+ CD45RA− CD8dim %CD8dim' on 'HCC'

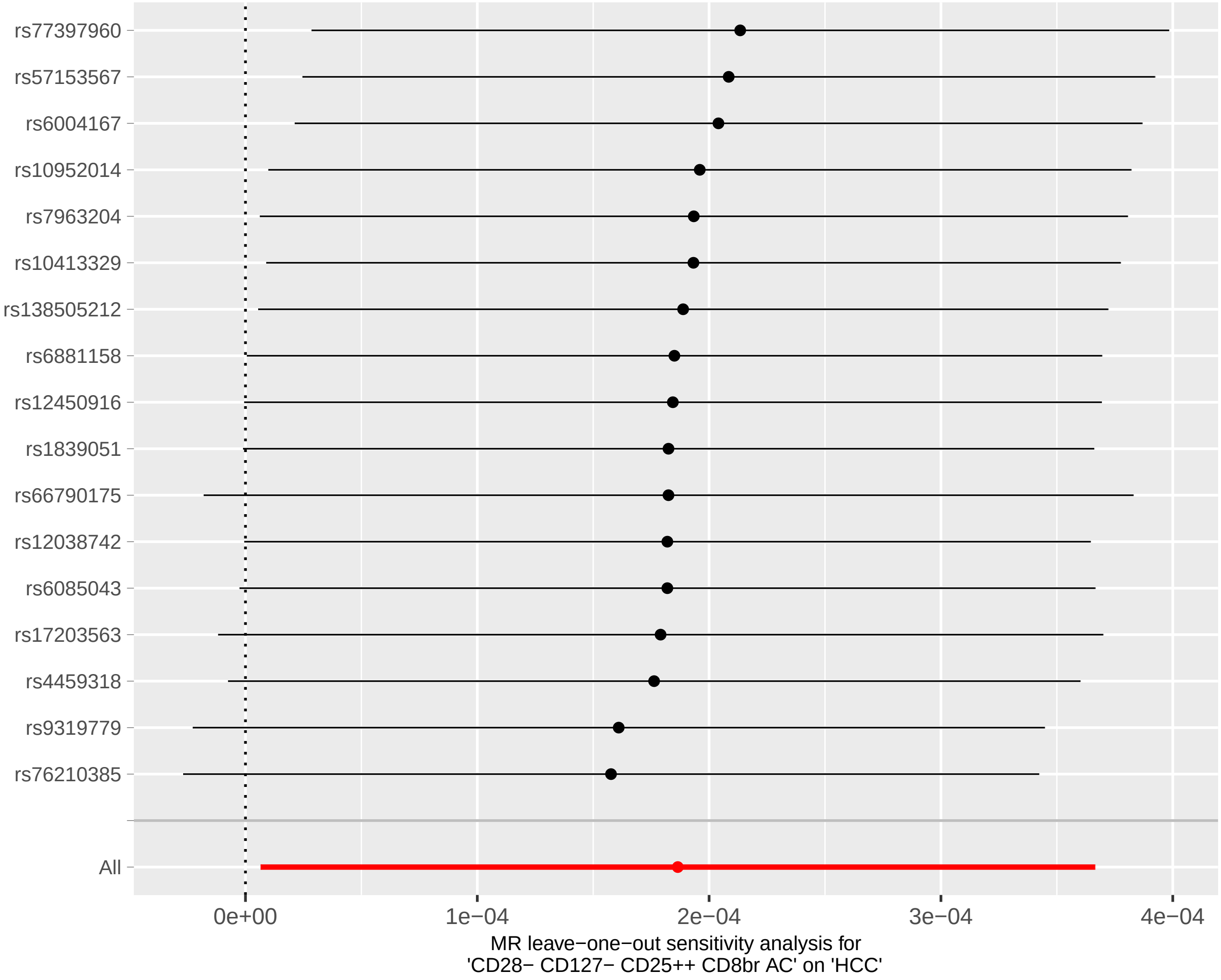
rs77397960
rs57153567
rs6004167
rs10952014
rs7963204
rs10413329
rs138505212
rs6881158
rs12450916
rs1839051
rs66790175
rs12038742
rs6085043
rs17203563
rs4459318
rs9319779
rs76210385
All
0e+00
1e−04
2e−04
3e−04
4e−04
MR leave−one−out sensitivity analysis for
'CD28− CD127− CD25++ CD8br AC' on 'HCC'

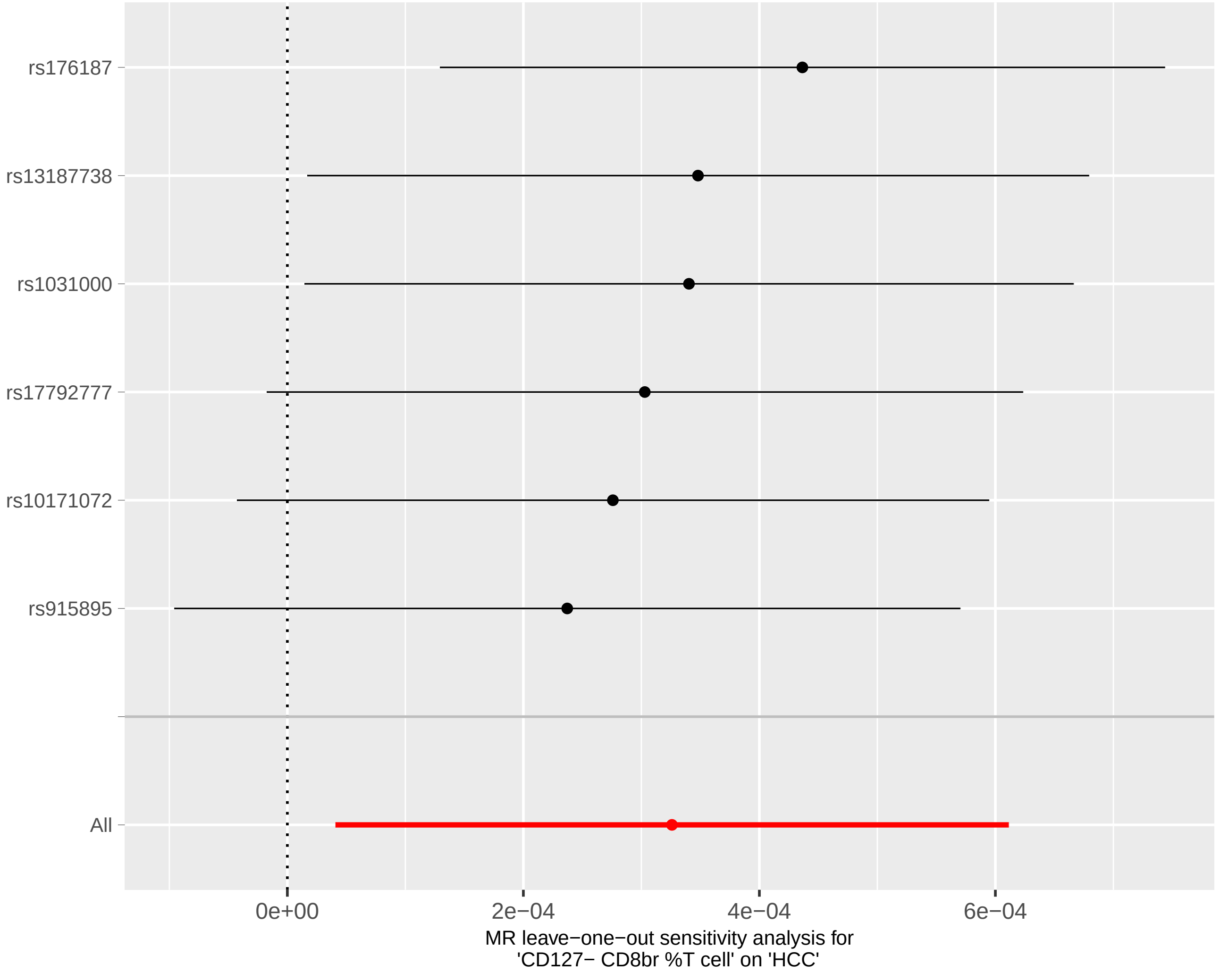
rs176187
rs13187738
rs1031000
rs17792777
rs10171072
rs915895
All
0e+00
2e−04
4e−04
6e−04
MR leave−one−out sensitivity analysis for
'CD127− CD8br %T cell' on 'HCC'

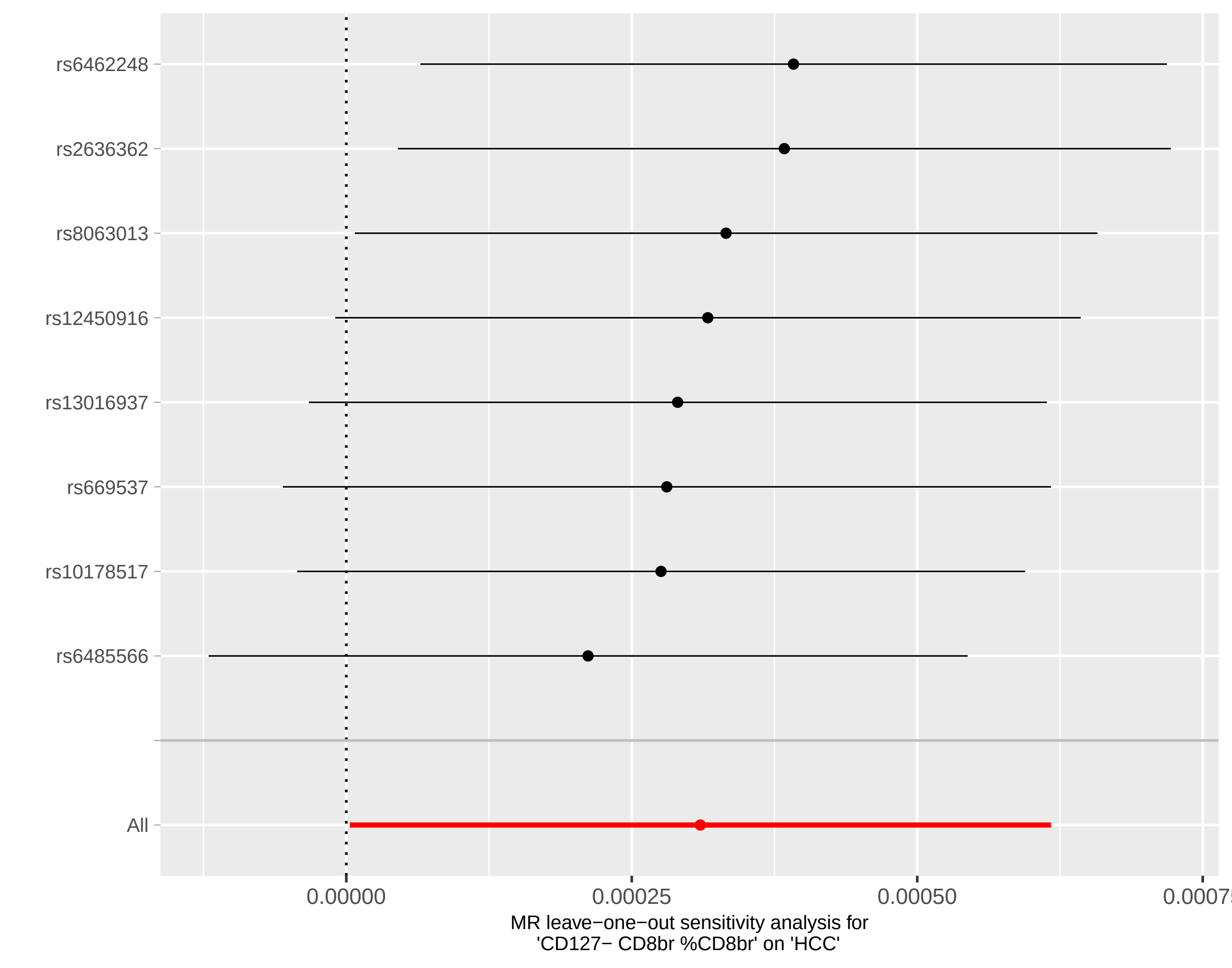
rs6462248
rs2636362
rs8063013
rs12450916
rs13016937
rs669537
rs10178517
rs6485566
All
0.00000
0.00025
0.00050
MR leave−one−out sensitivity analysis for
'CD127− CD8br %CD8br' on 'HCC'

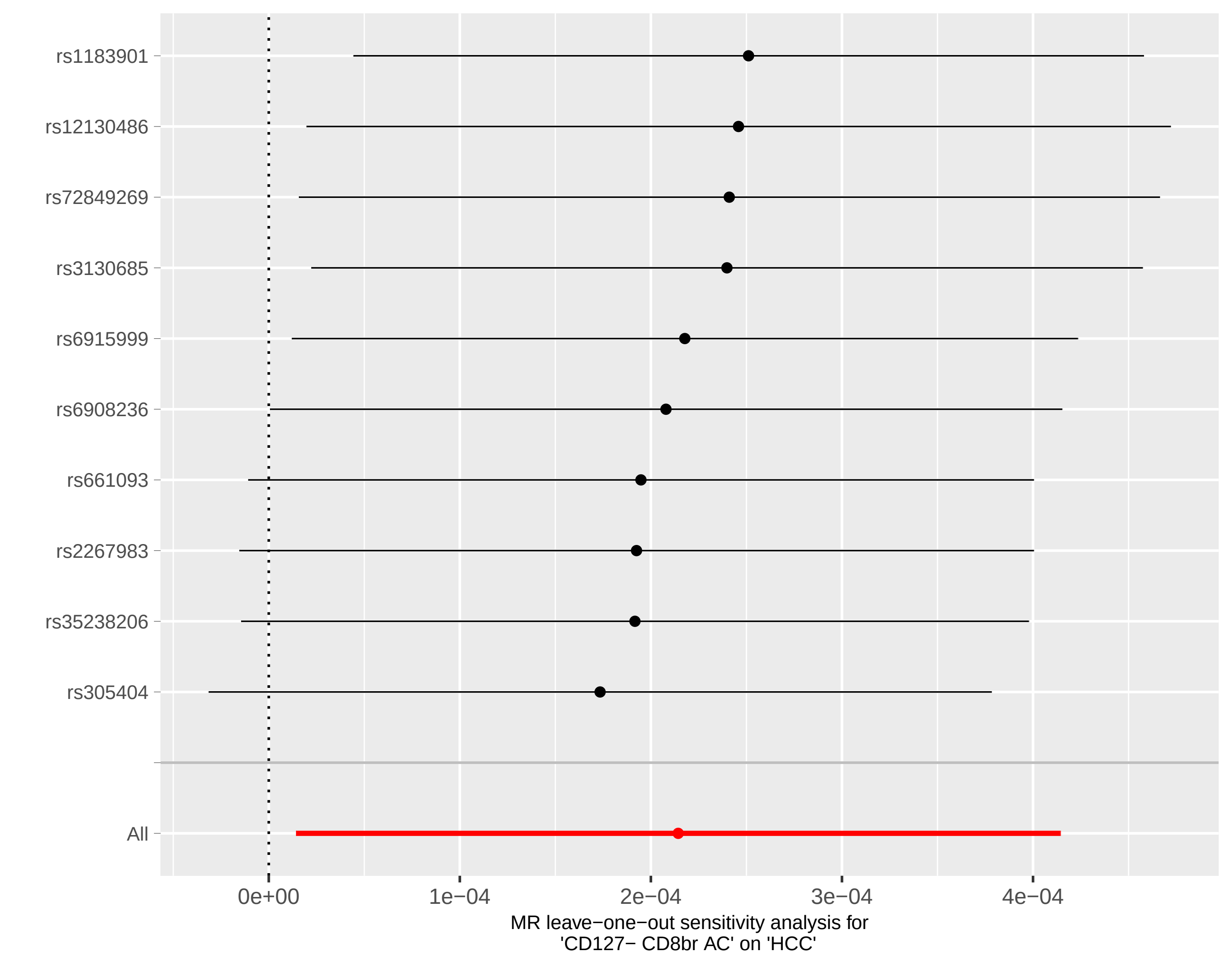
rs1183901
rs12130486
rs72849269
rs3130685
rs6915999
rs6908236
rs661093
rs2267983
rs35238206
rs305404
All
0e+00
1e−04
2e−04
3e−04
4e−04
MR leave−one−out sensitivity analysis for
'CD127− CD8br AC' on 'HCC'

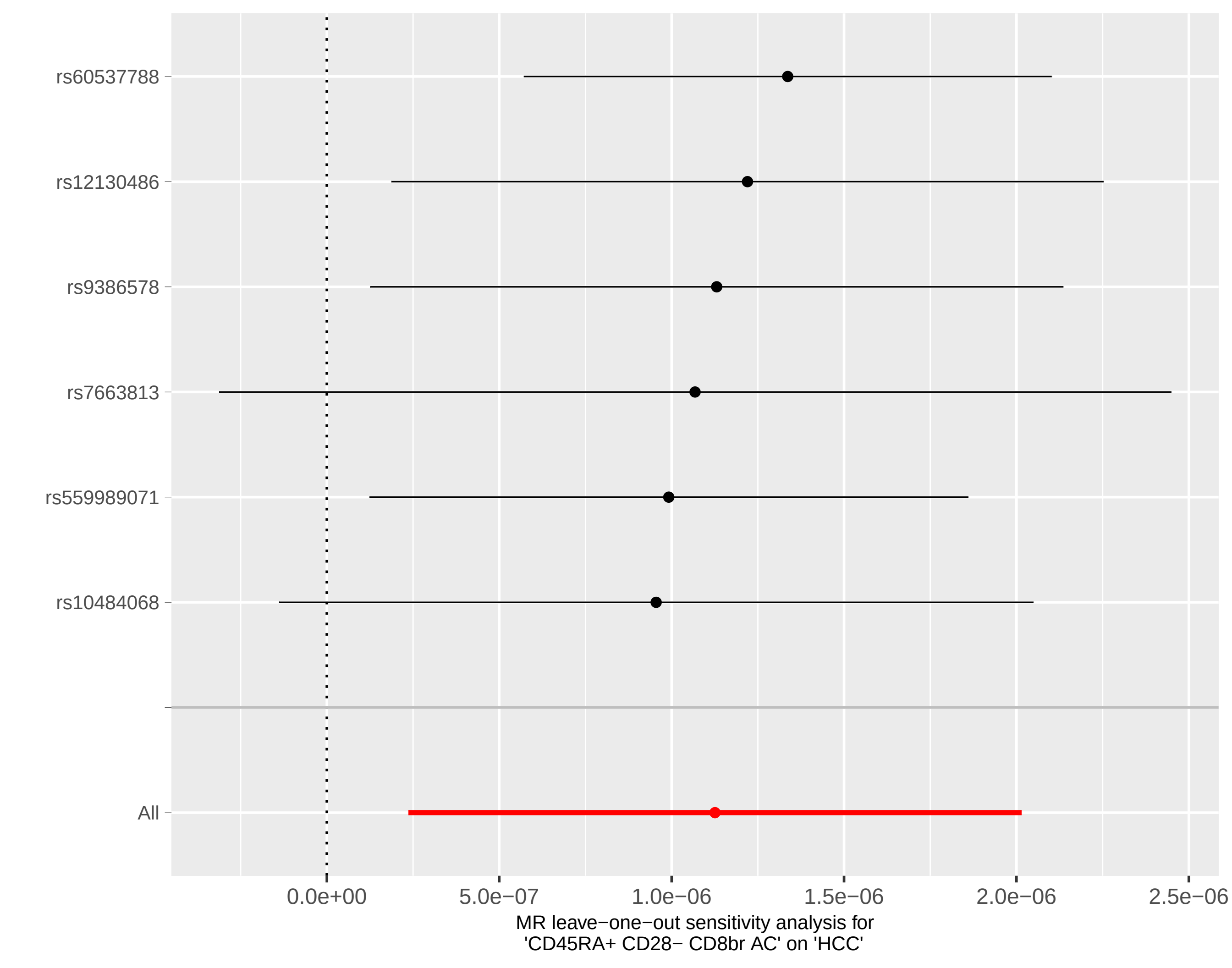
rs60537788
rs12130486
rs9386578
rs7663813
rs559989071
rs10484068
All
0.0e+00
5.0e−07
1.0e−06
1.5e−06
2.0e−06
2.5e−06
MR leave−one−out sensitivity analysis for
'CD45RA+ CD28− CD8br AC' on 'HCC'

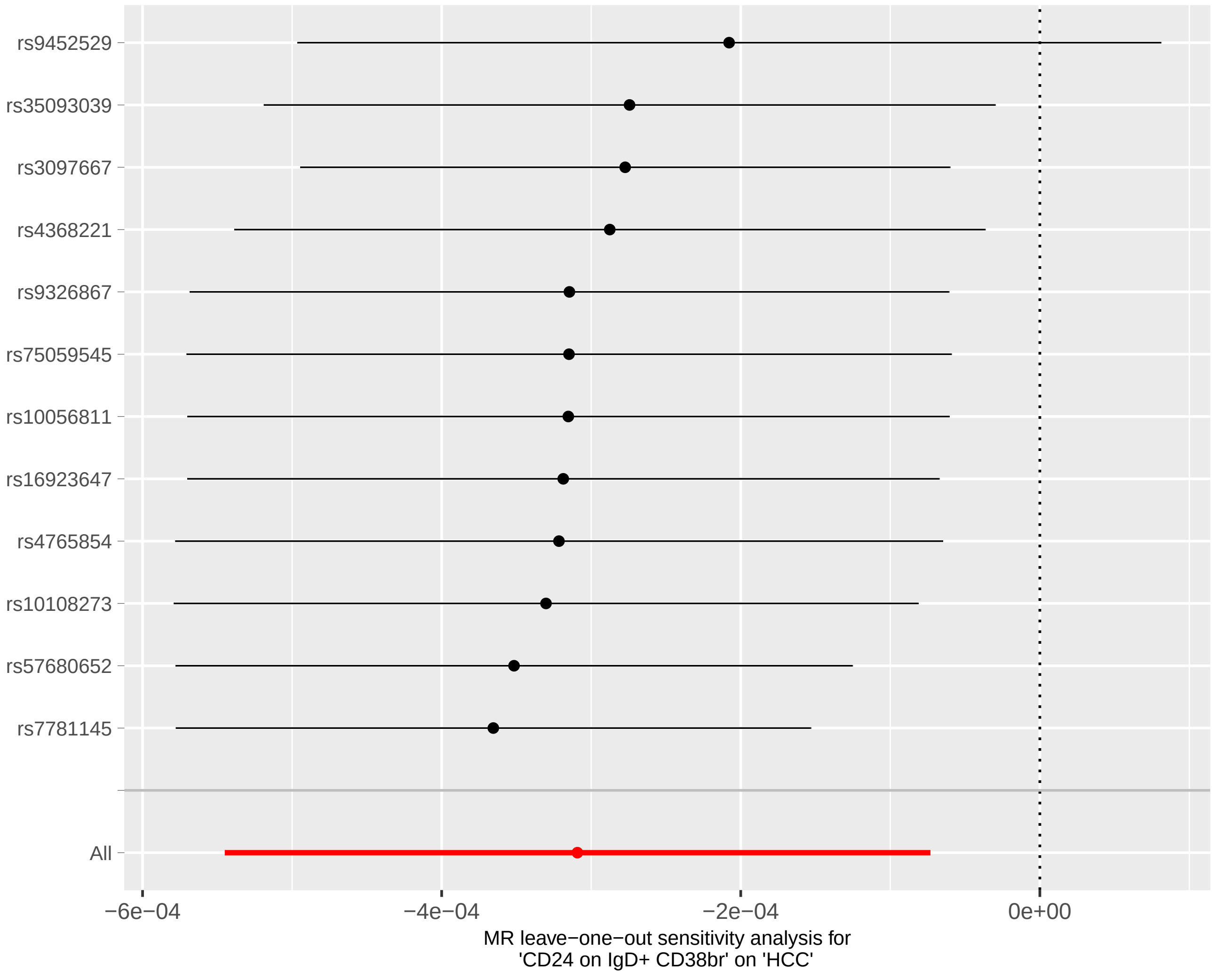
rs9452529
rs35093039
rs3097667
rs4368221
rs9326867
rs75059545
rs10056811
rs16923647
rs4765854
rs10108273
rs57680652
rs7781145
All
−6e−04
−4e−04
−2e−04
0e+00
MR leave−one−out sensitivity analysis for
'CD24 on IgD+ CD38br' on 'HCC'

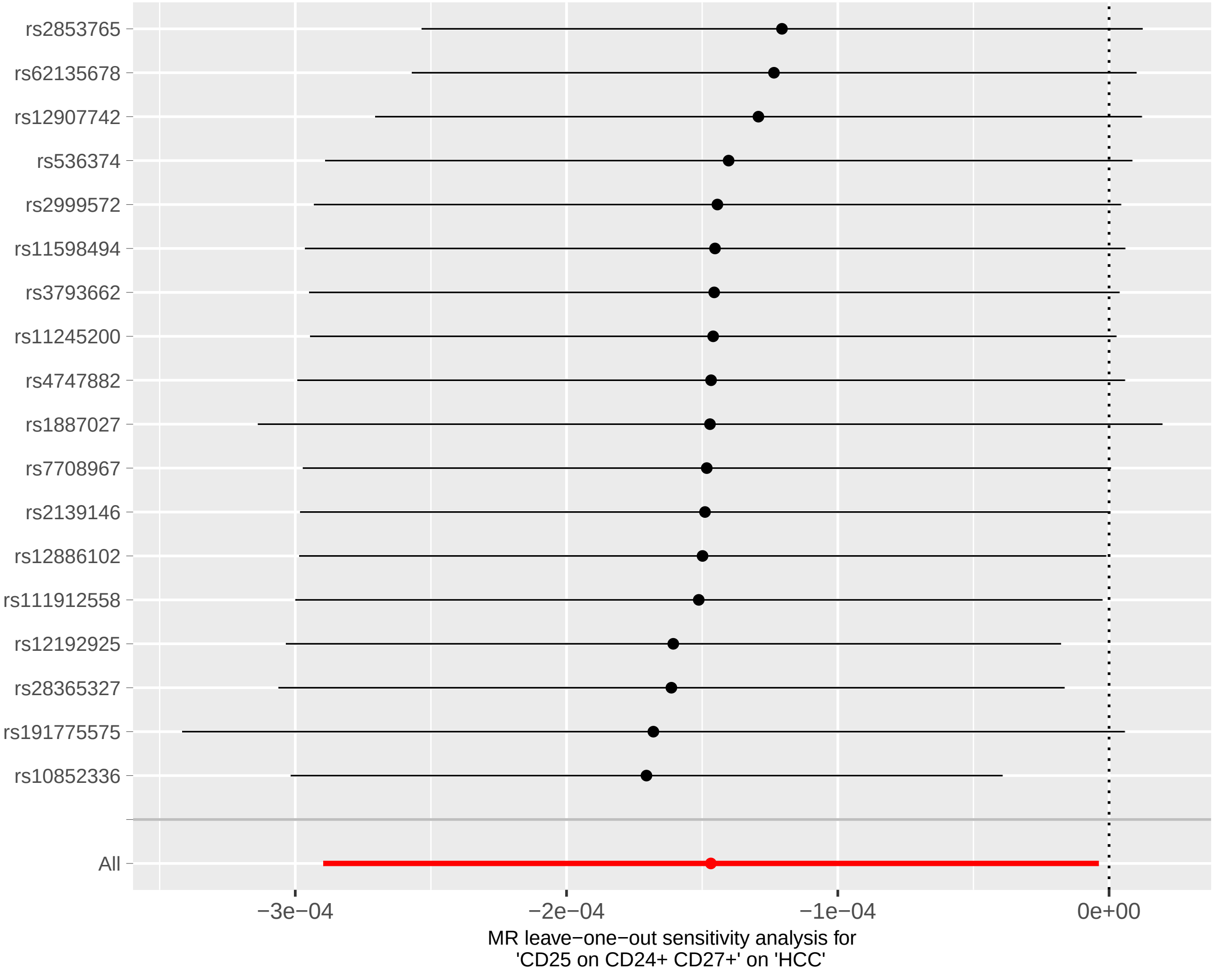
rs2853765
rs62135678
rs12907742
rs536374
rs2999572
rs11598494
rs3793662
rs11245200
rs4747882
rs1887027
rs7708967
rs2139146
rs12886102
rs111912558
rs12192925
rs28365327
rs191775575
rs10852336
All
−3e−04
−2e−04
−1e−04
0e+00
MR leave−one−out sensitivity analysis for
'CD25 on CD24+ CD27+' on 'HCC'

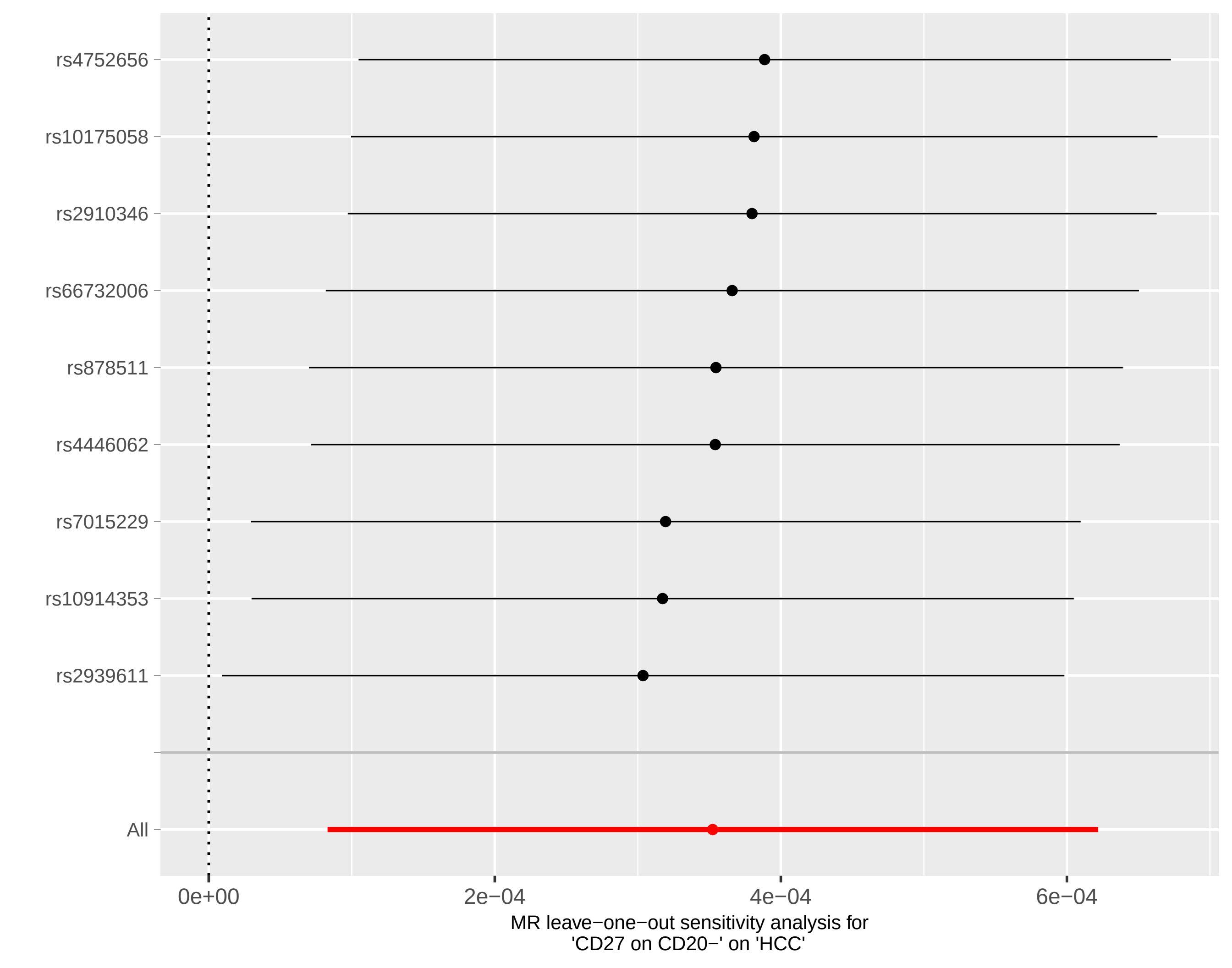
rs4752656
rs10175058
rs2910346
rs66732006
rs878511
rs4446062
rs7015229
rs10914353
rs2939611
All
0e+00
2e−04
4e−04
6e−04
MR leave−one−out sensitivity analysis for
'CD27 on CD20−' on 'HCC'

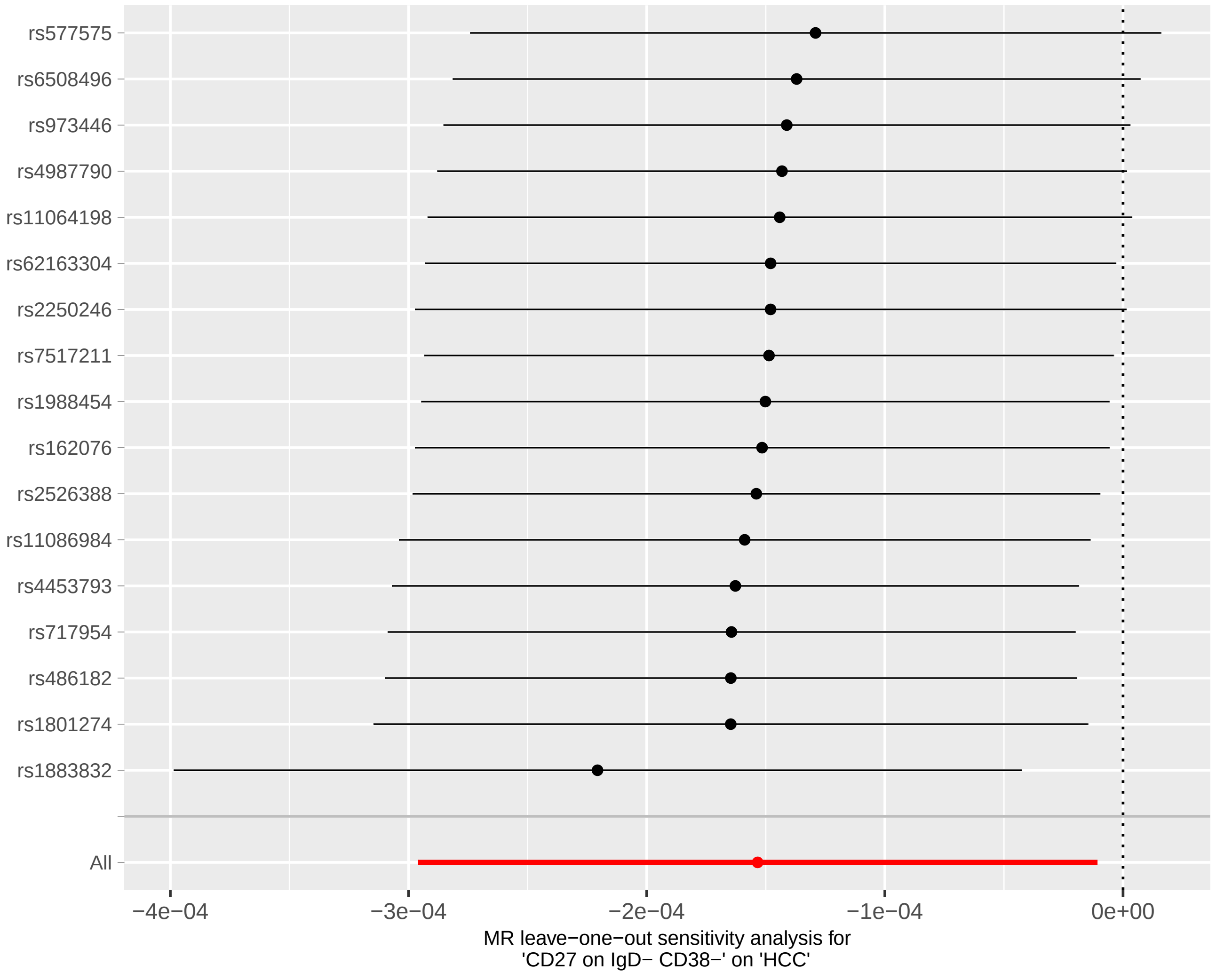
rs577575
rs6508496
rs973446
rs4987790
rs11064198
rs62163304
rs2250246
rs7517211
rs1988454
rs162076
rs2526388
rs11086984
rs4453793
rs717954
rs486182
rs1801274
rs1883832
All
−4e−04
−3e−04
−2e−04
−1e−04
0e+00
MR leave−one−out sensitivity analysis for
'CD27 on IgD− CD38−' on 'HCC'

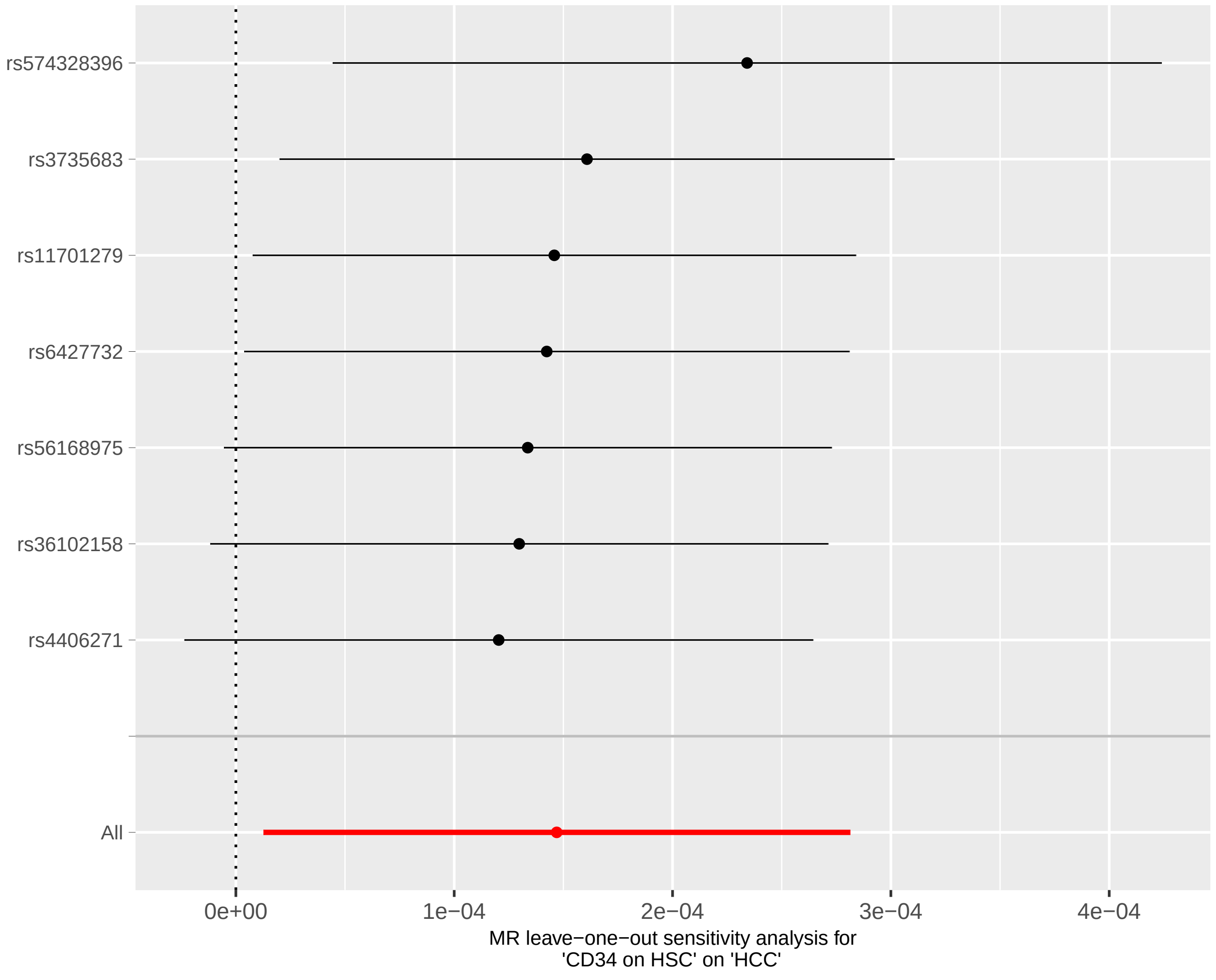

rs574328396
rs3735683
rs11701279
rs6427732
rs56168975
rs36102158
rs4406271
All
0e+00
1e−04
2e−04
3e−04
4e−04
MR leave−one−out sensitivity analysis for
'CD34 on HSC' on 'HCC'

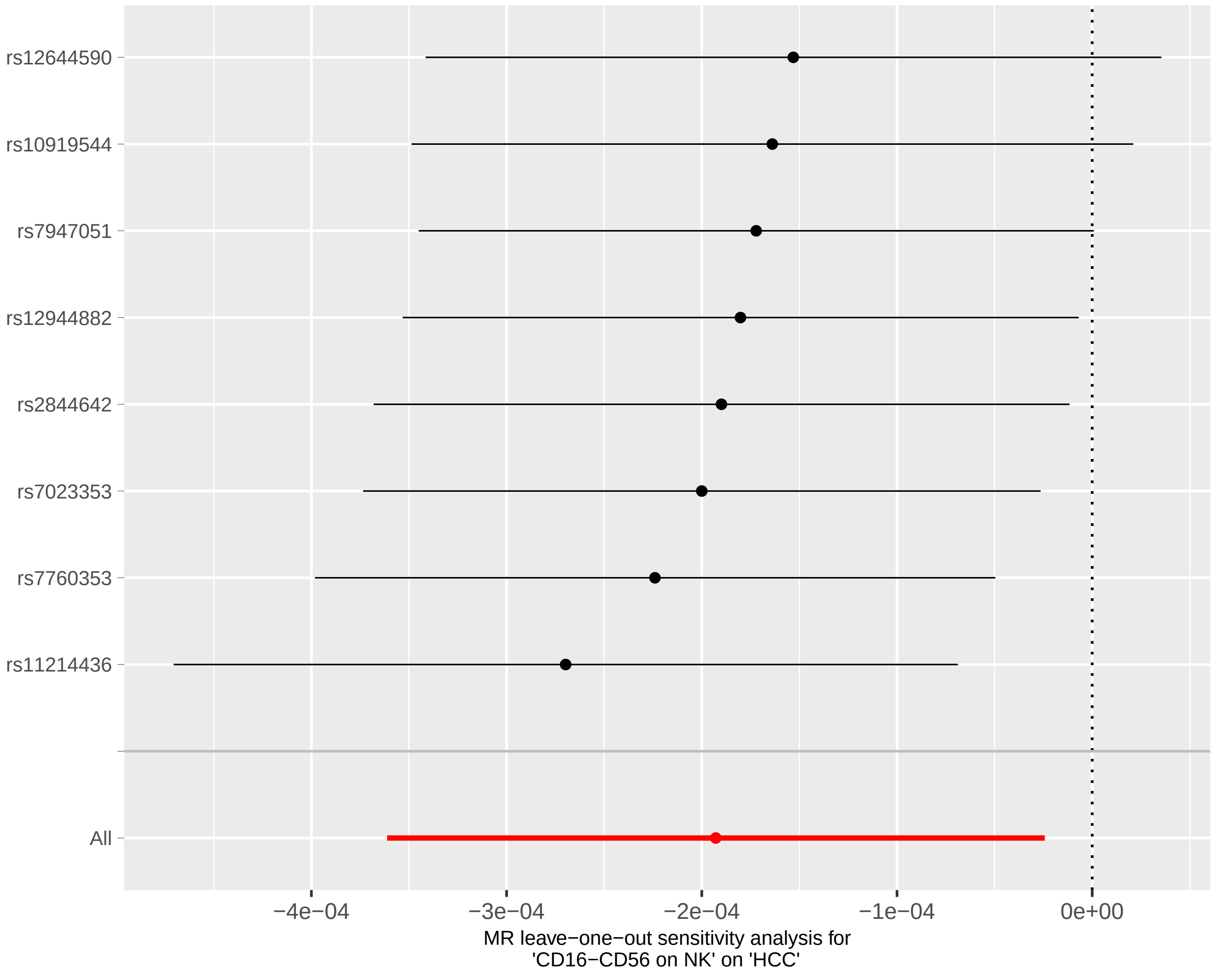

rs12644590
rs10919544
rs7947051
rs12944882
rs2844642
rs7023353
rs7760353
rs11214436
All
−4e−04
−3e−04
−2e−04
−1e−04
0e+00
MR leave−one−out sensitivity analysis for
'CD16−CD56 on NK' on 'HCC'

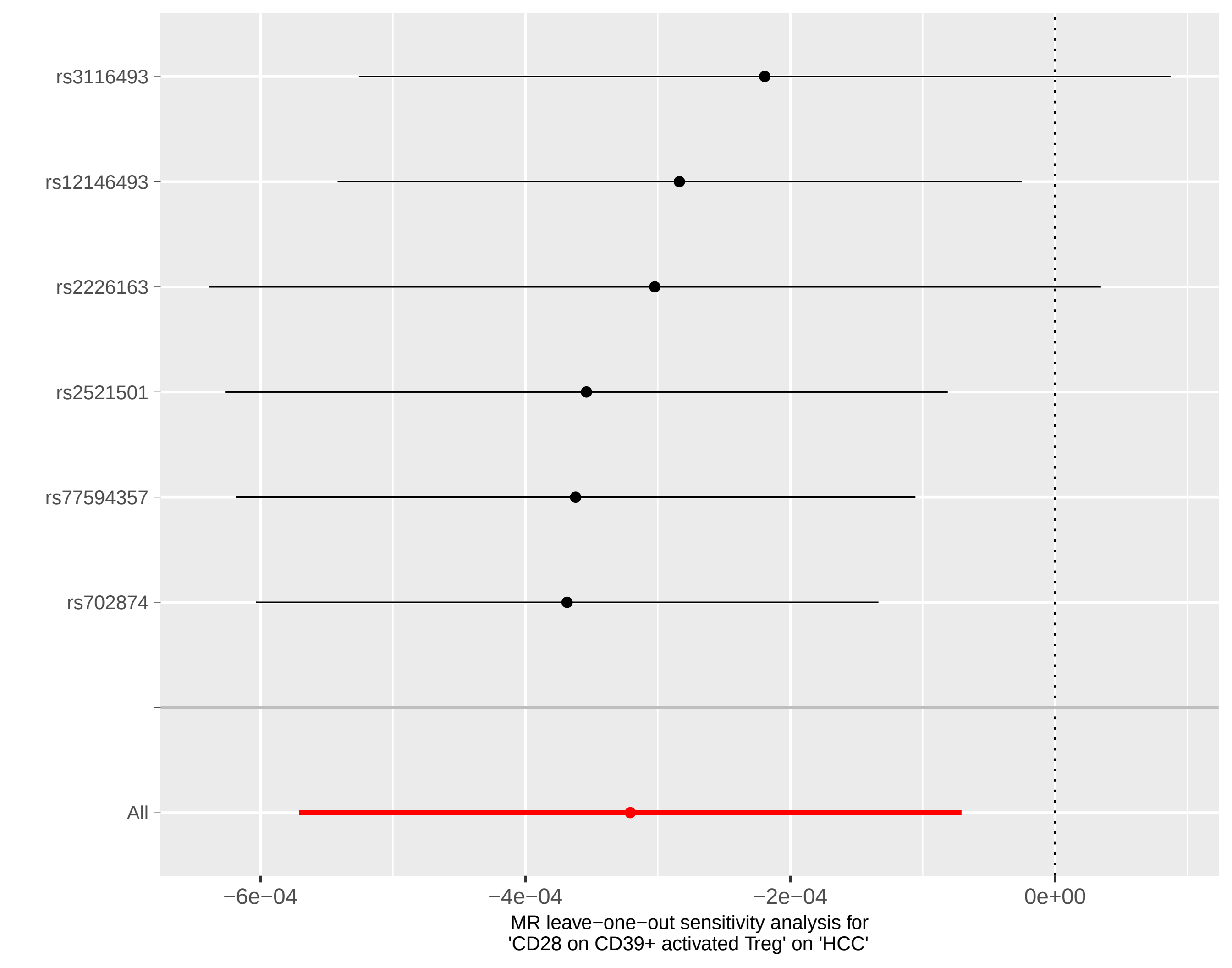
rs3116493
rs12146493
rs2226163
rs2521501
rs77594357
rs702874
All
−6e−04
−4e−04
−2e−04
0e+00
MR leave−one−out sensitivity analysis for
'CD28 on CD39+ activated Treg' on 'HCC'

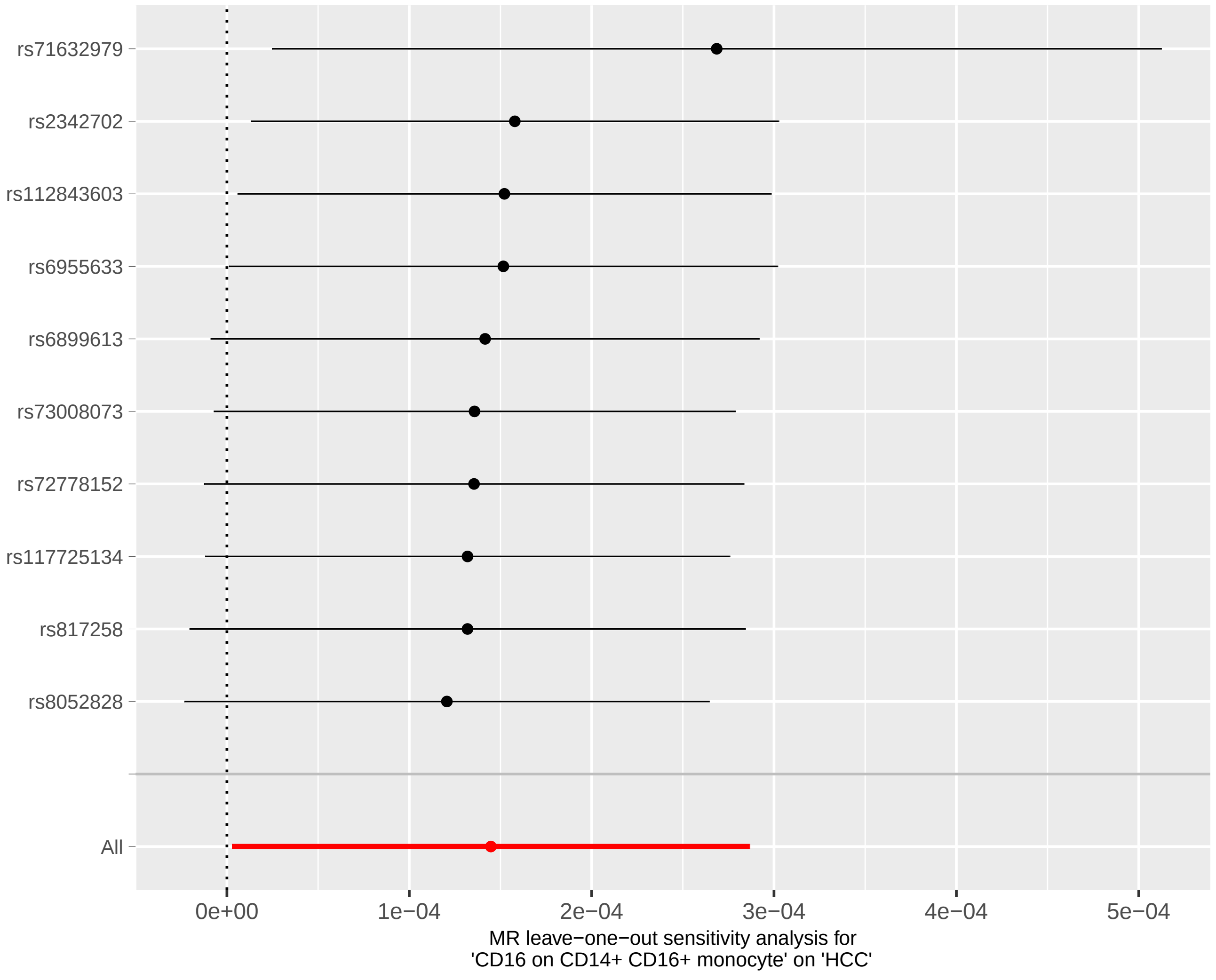
rs71632979
rs2342702
rs112843603
rs6955633
rs6899613
rs73008073
rs72778152
rs117725134
rs817258
rs8052828
All
0e+00
1e−04
2e−04
3e−04
4e−04
5e−04
MR leave−one−out sensitivity analysis for
'CD16 on CD14+ CD16+ monocyte' on 'HCC'

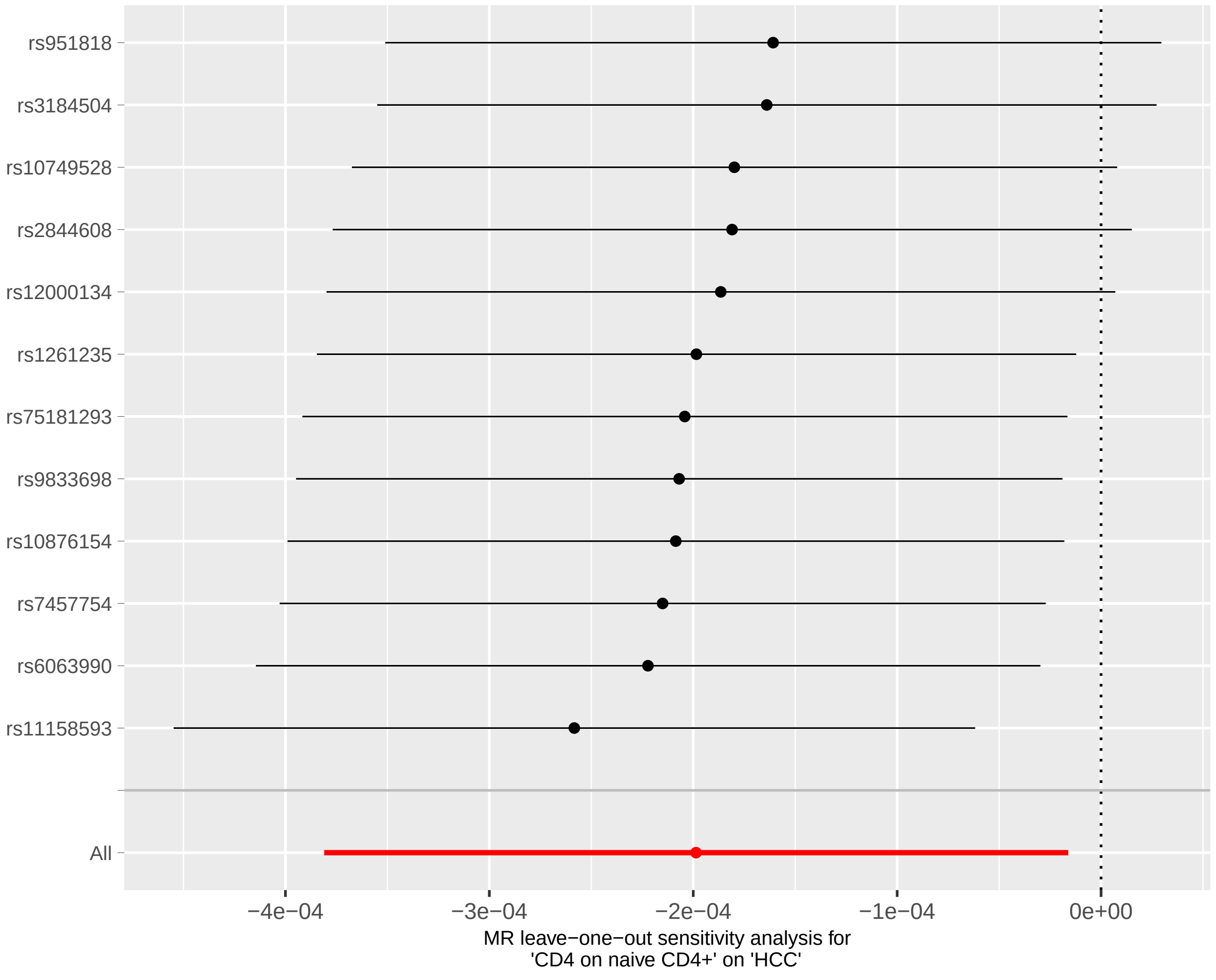
rs951818
rs3184504
rs10749528
rs2844608
rs12000134
rs1261235
rs75181293
rs9833698
rs10876154
rs7457754
rs6063990
rs11158593
All
−4e−04
−3e−04
−2e−04
−1e−04
0e+00
MR leave−one−out sensitivity analysis for
'CD4 on naive CD4+' on 'HCC'

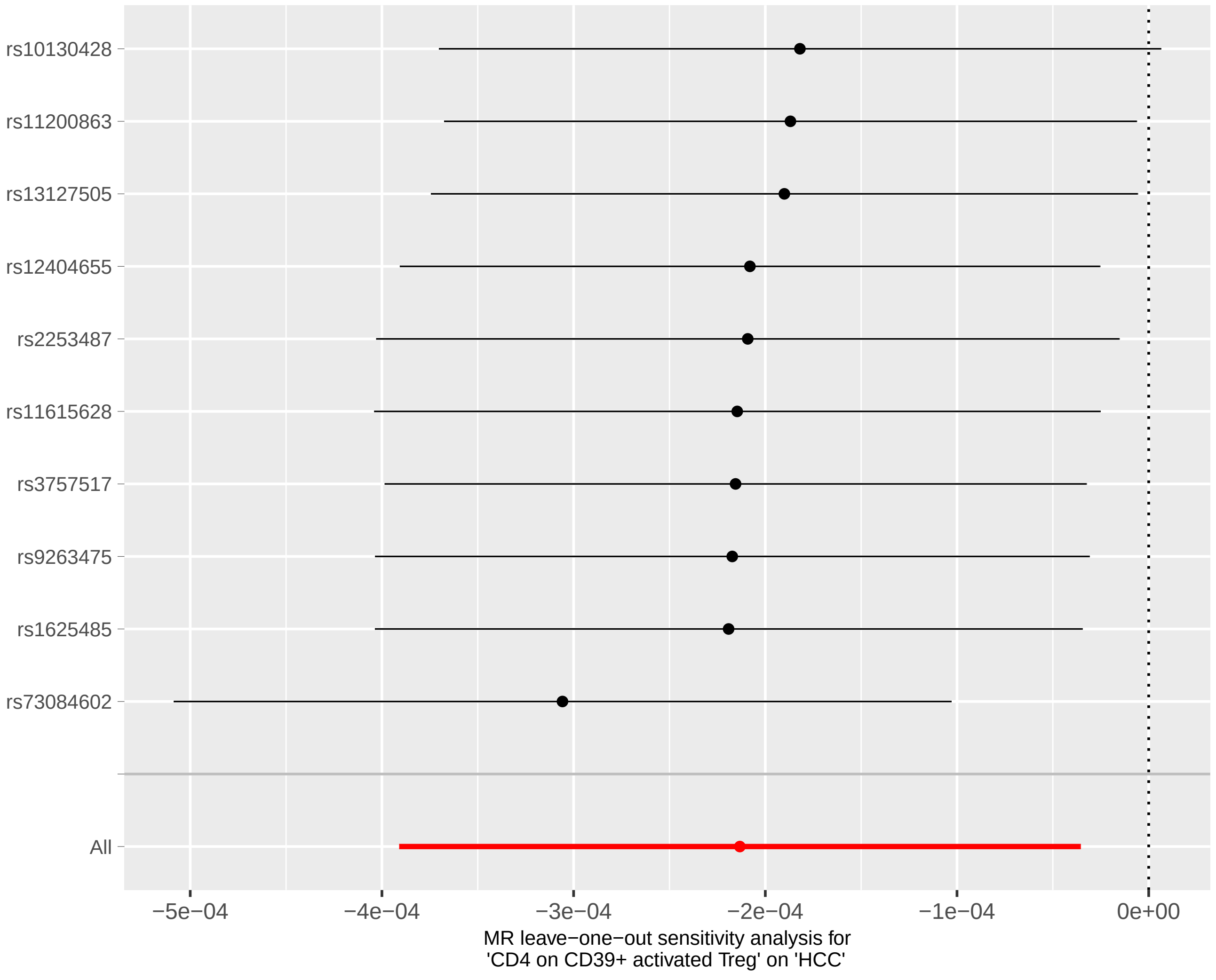
rs10130428
rs11200863
rs13127505
rs12404655
rs2253487
rs11615628
rs3757517
rs9263475
rs1625485
rs73084602
All
−5e−04
−4e−04
−3e−04
−2e−04
−1e−04
0e+00
MR leave−one−out sensitivity analysis for
'CD4 on CD39+ activated Treg' on 'HCC'

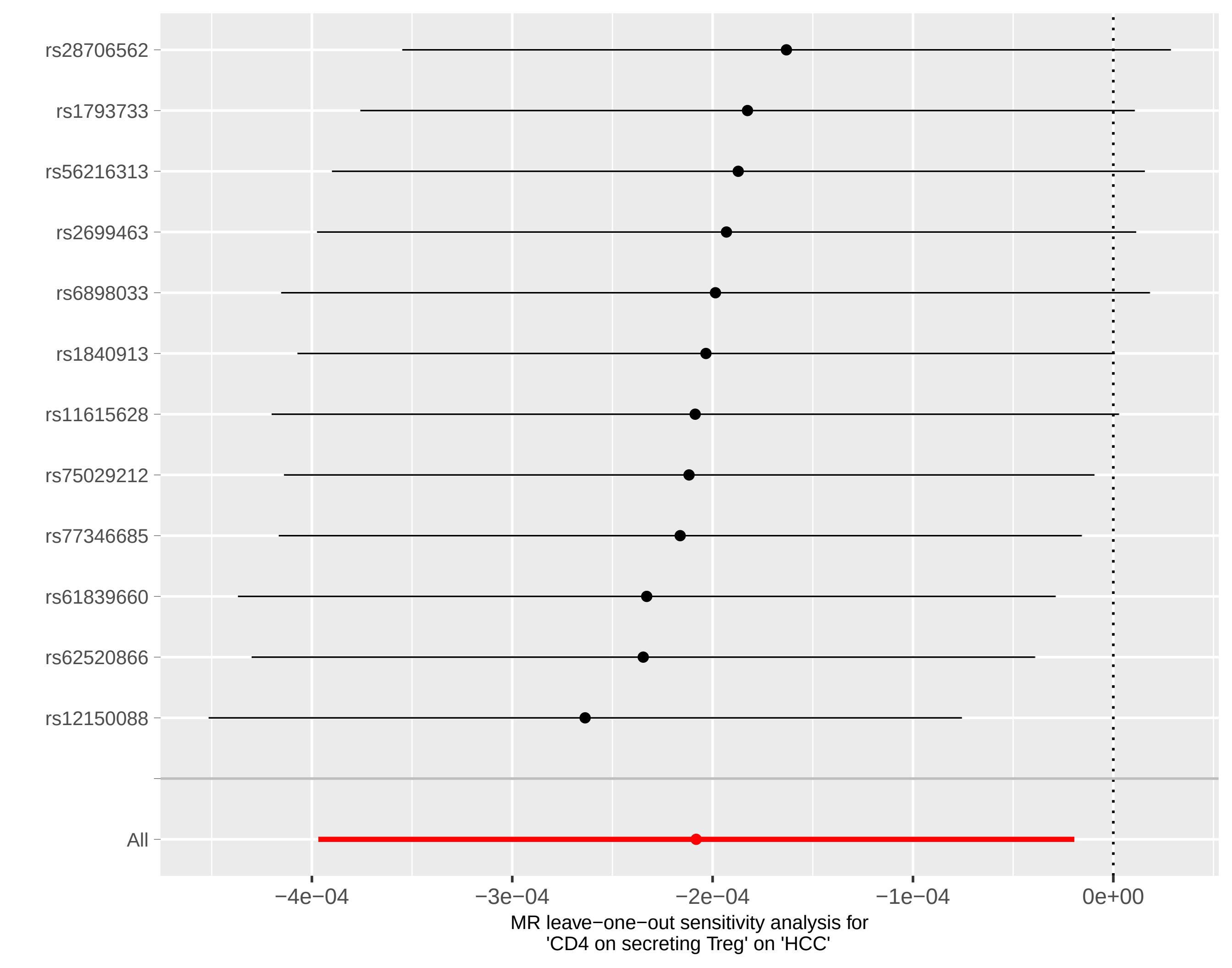

rs28706562
rs1793733
rs56216313
rs2699463
rs6898033
rs1840913
rs11615628
rs75029212
rs77346685
rs61839660
rs62520866
rs12150088
All
−4e−04
−3e−04
−2e−04
−1e−04
0e+00
MR leave−one−out sensitivity analysis for
'CD4 on secreting Treg' on 'HCC'

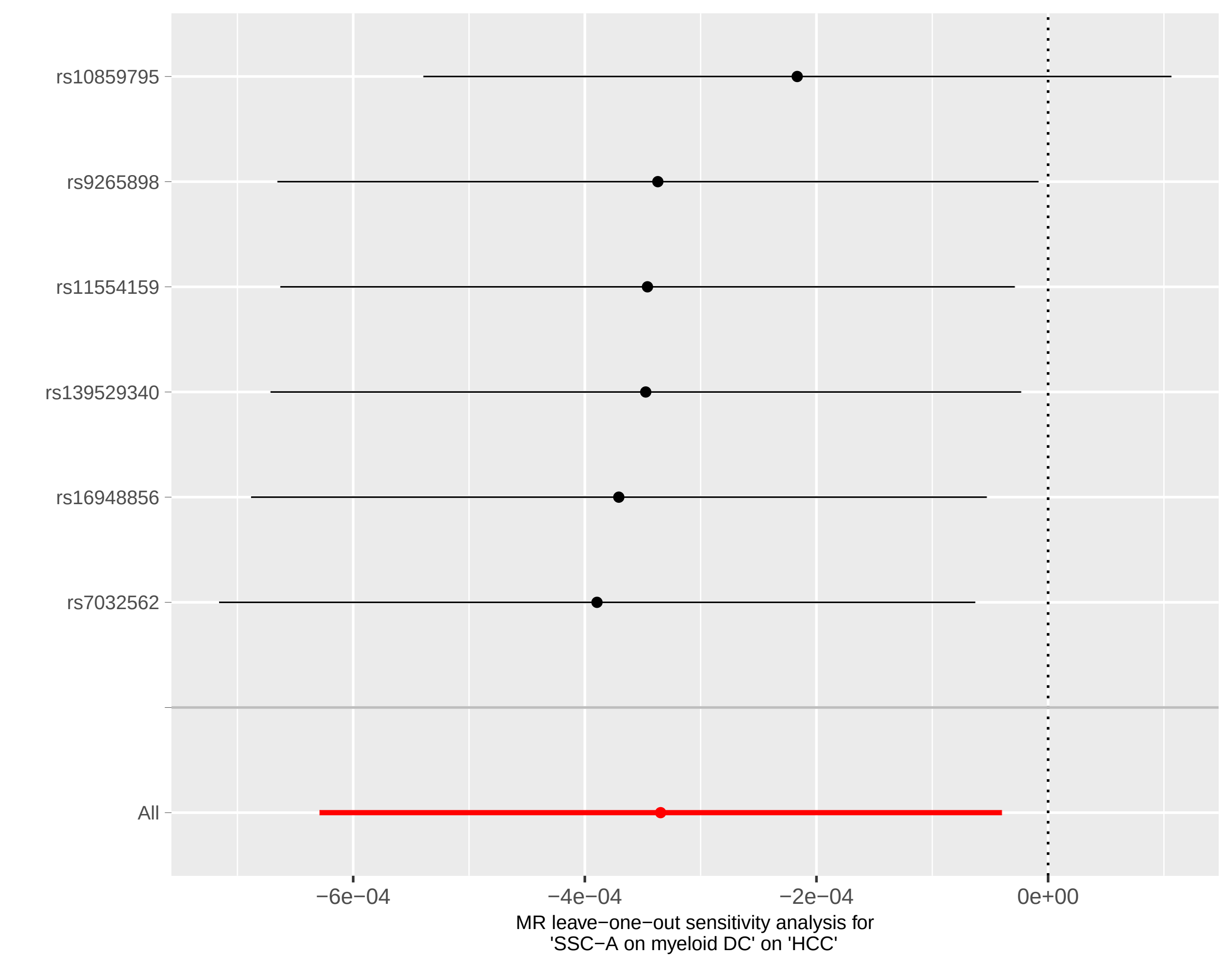
rs10859795
rs9265898
rs11554159
rs139529340
rs16948856
rs7032562
All
−6e−04
−4e−04
−2e−04
0e+00
MR leave−one−out sensitivity analysis for
'SSC−A on myeloid DC' on 'HCC'

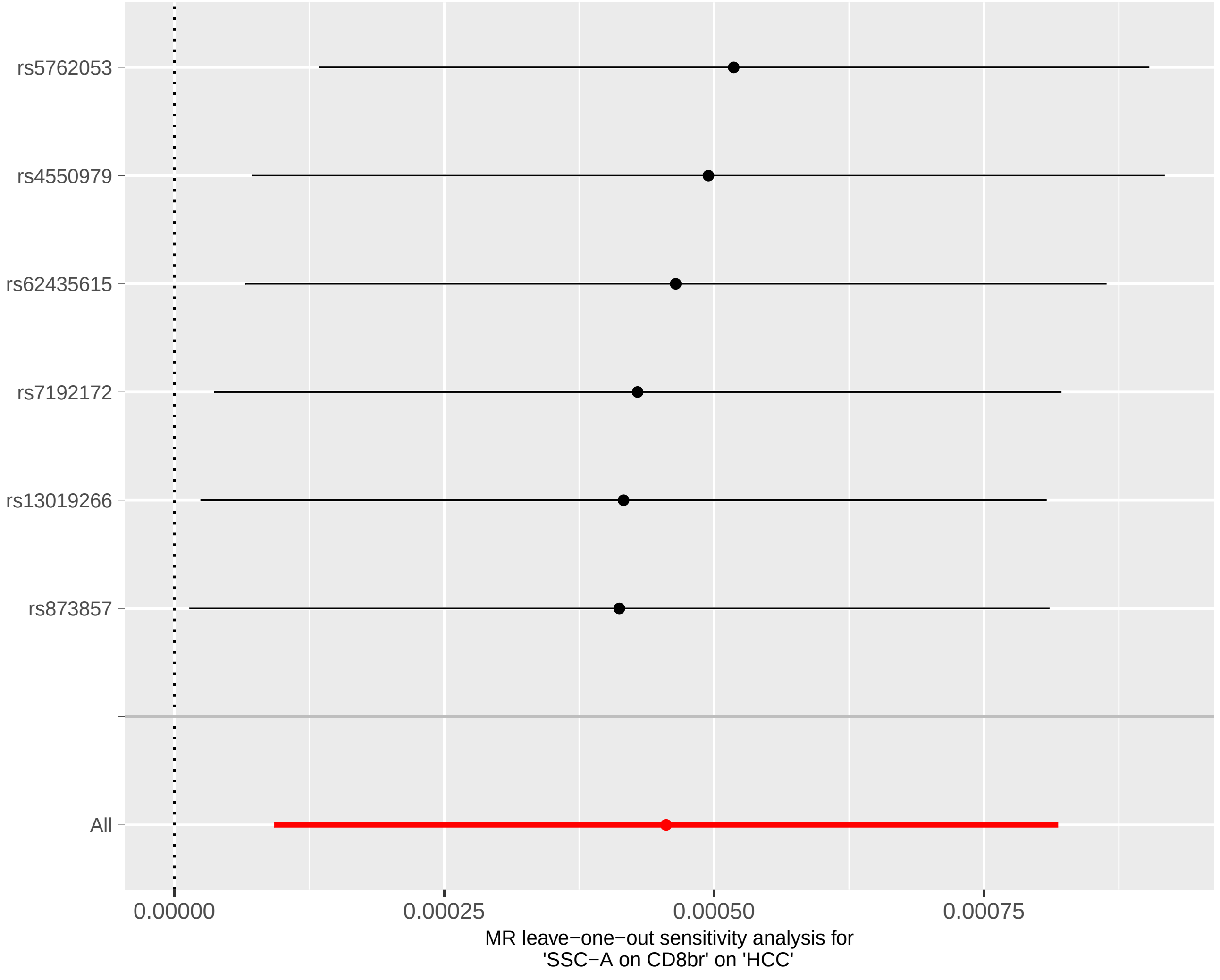

rs5762053
rs4550979
rs62435615
rs7192172
rs13019266
rs873857
All
0.00000
0.00025
0.00050
0.00075
MR leave−one−out sensitivity analysis for
'SSC−A on CD8br' on 'HCC'

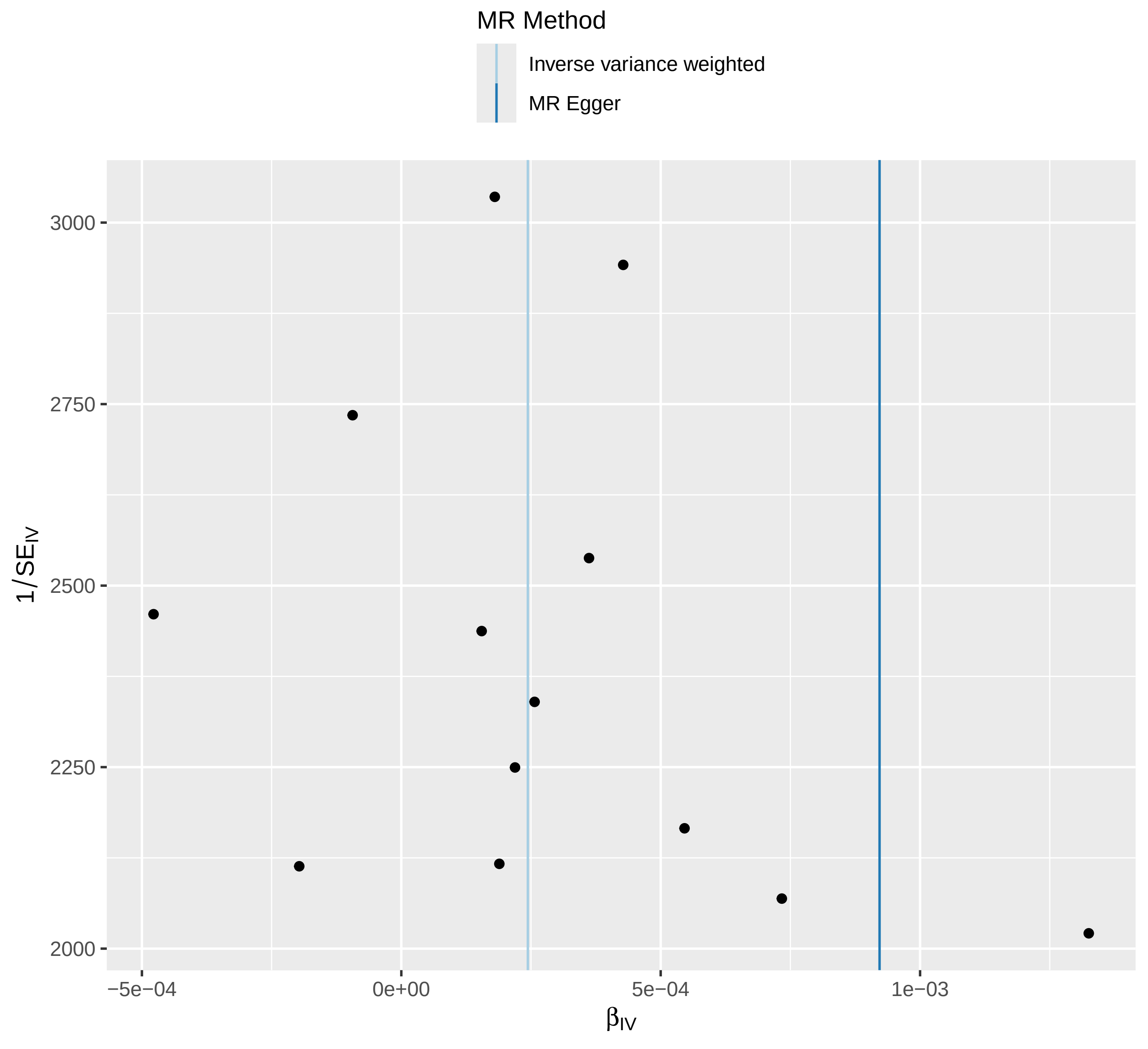
MR Method
Inverse variance weighted
MR Egger
3000
2750
2500
2250
2000
1/SE_IV
−5e−04
0e+00
5e−04
1e−03
β_IV

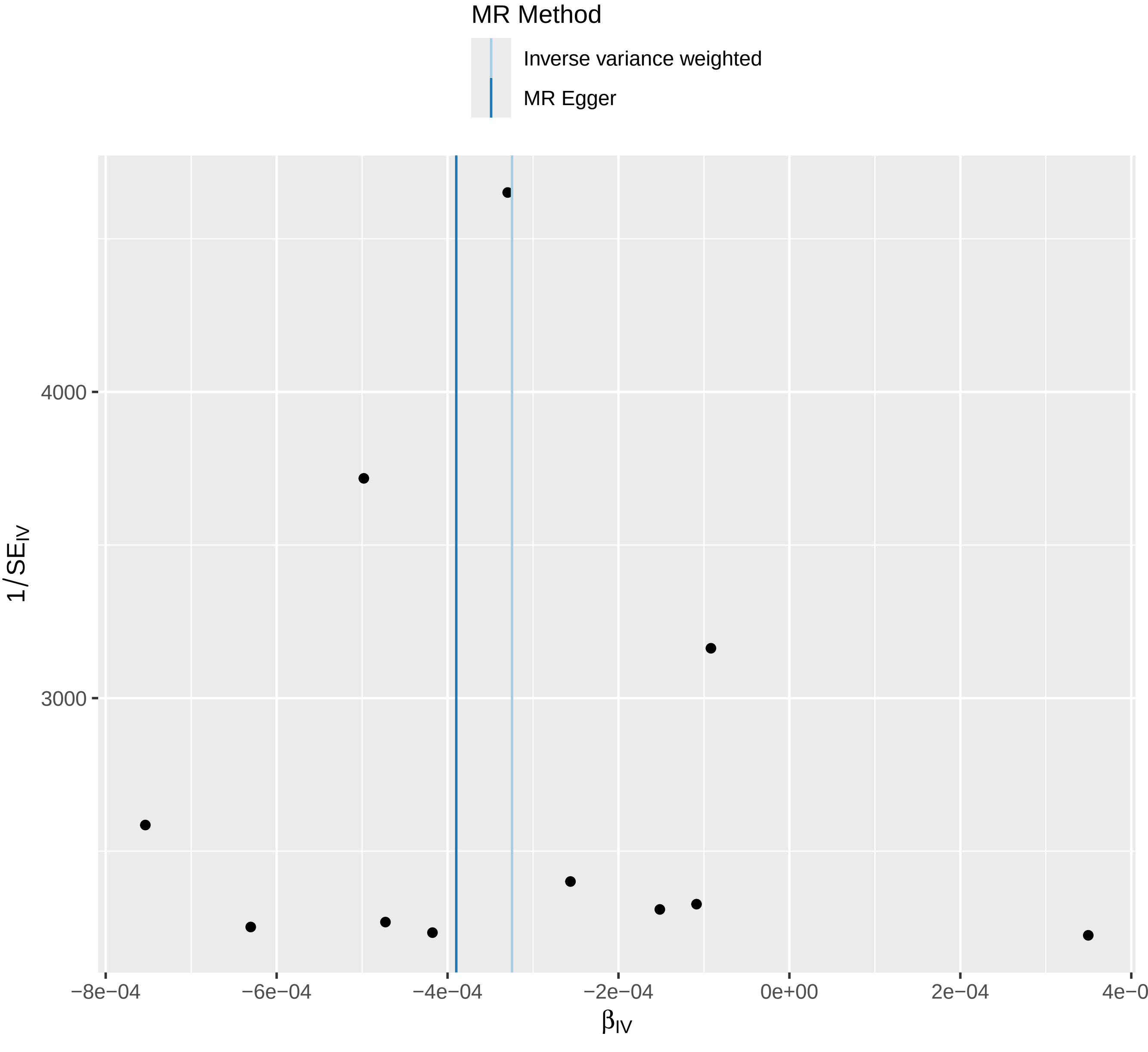

MR Method
Inverse variance weighted
MR Egger
4000
3000
1/SE_IV
−8e−04
−6e−04
−4e−04
−2e−04
0e+00
2e−04
4e−0
β_IV

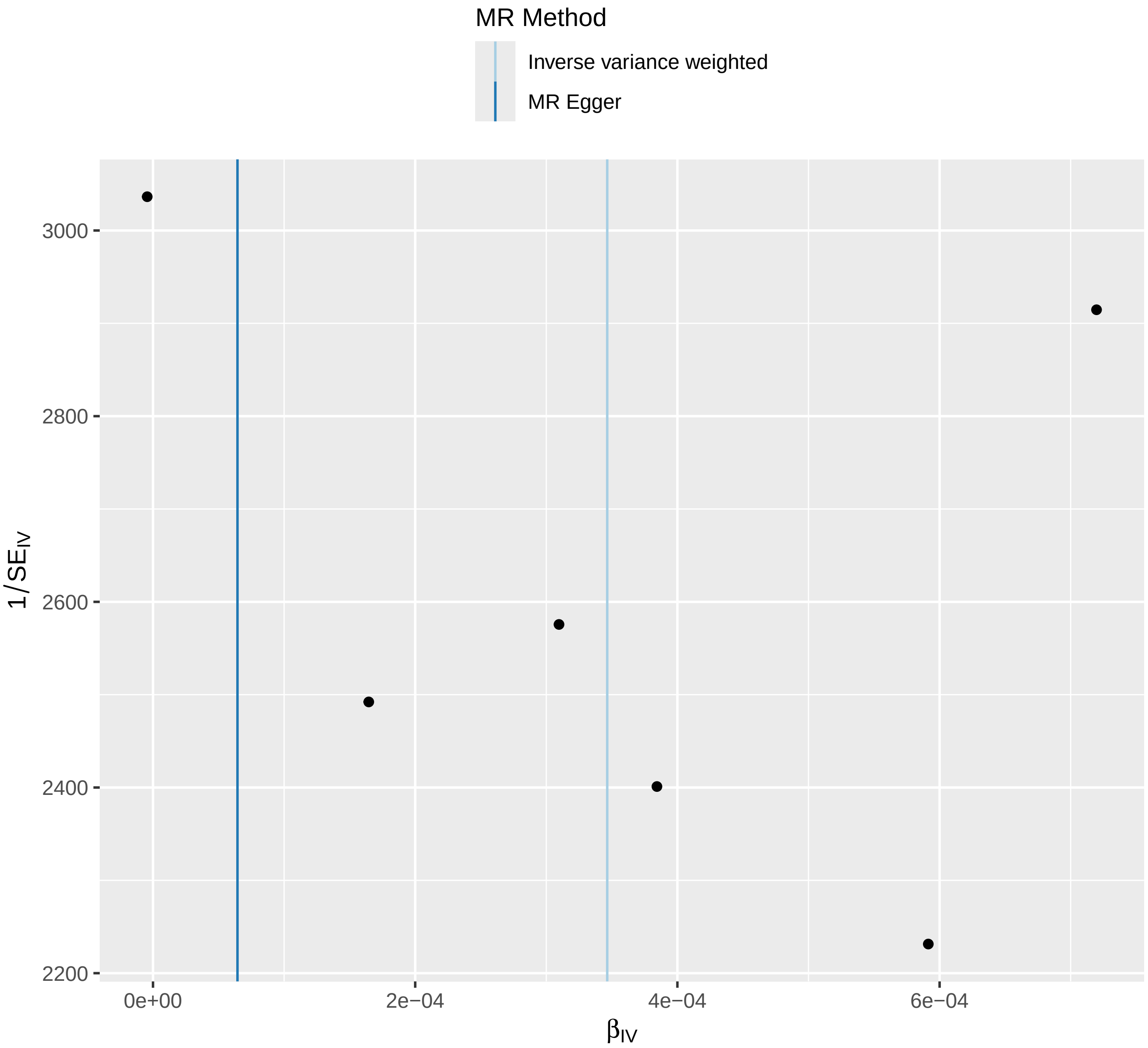
MR Method
Inverse variance weighted
MR Egger
3000
2800
2600
2400
2200
$1/SE_{IV}$
0e+00
2e−04
4e−04
6e−04
β_{IV}

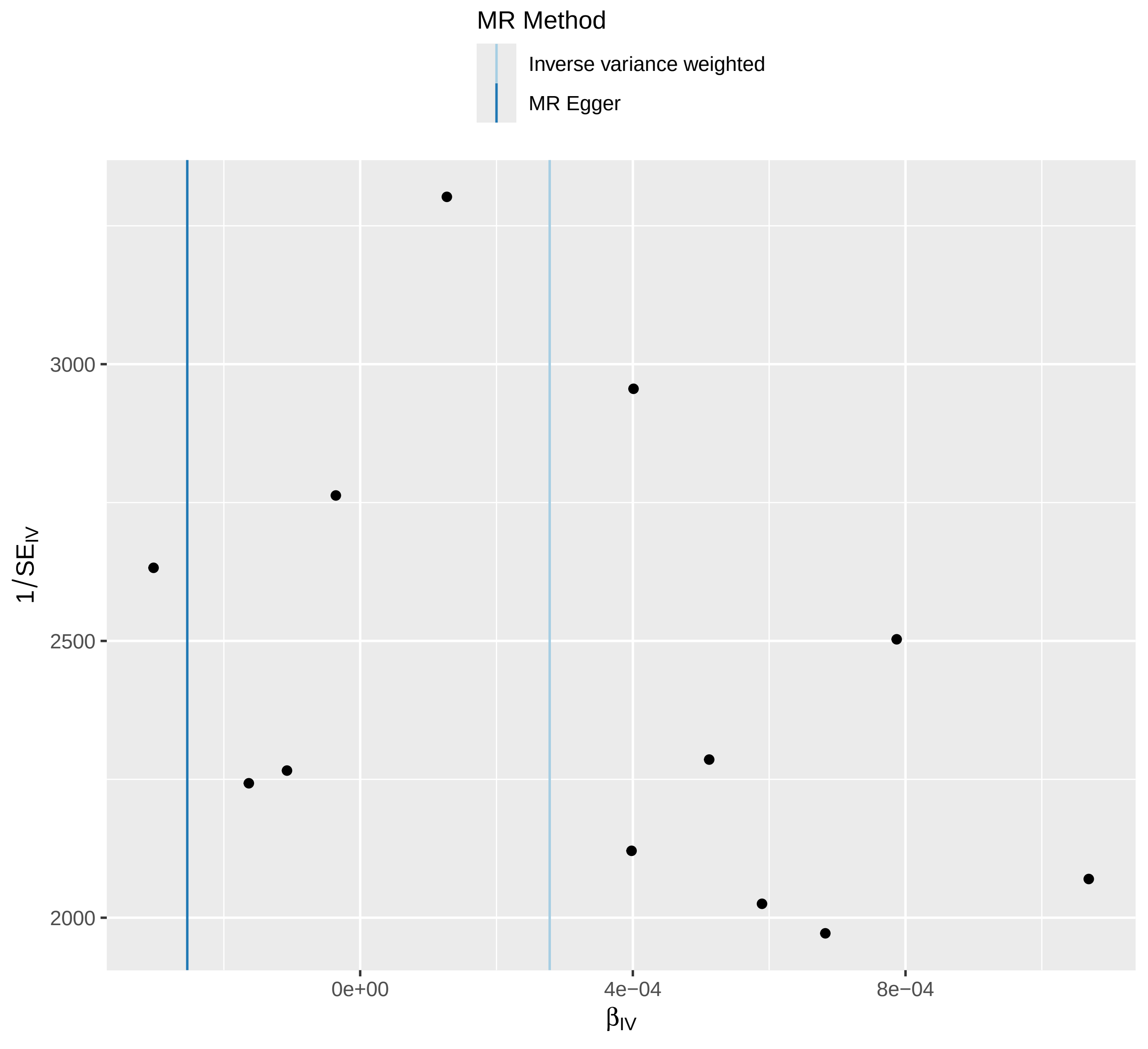
MR Method
Inverse variance weighted
MR Egger
3000
2500
2000
1/SE_IV
0e+00
4e−04
8e−04
β_IV

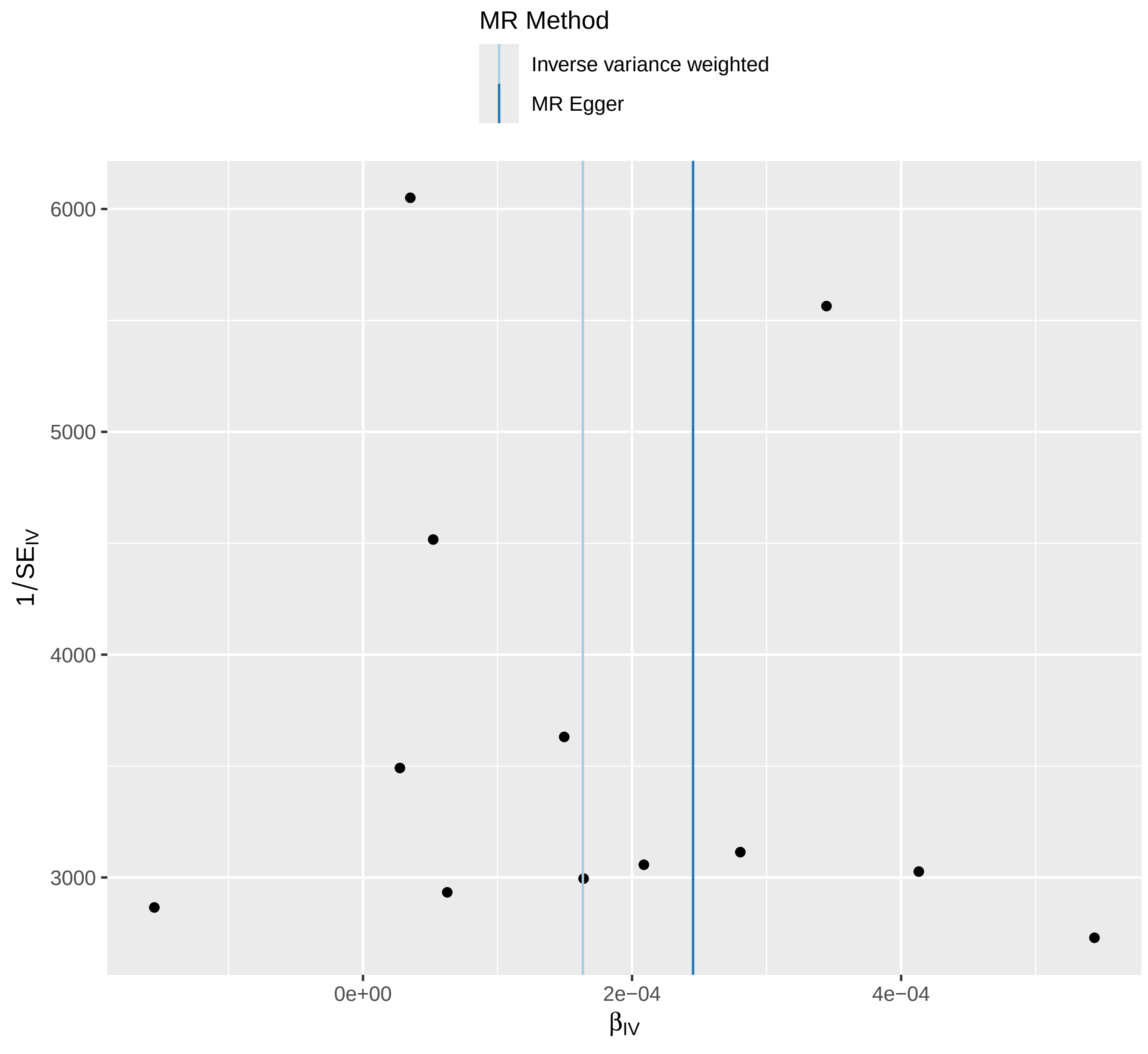
MR Method
Inverse variance weighted
MR Egger
6000
5000
4000
3000
$1/SE_{IV}$
0e+00
2e−04
4e−04
β_{IV}

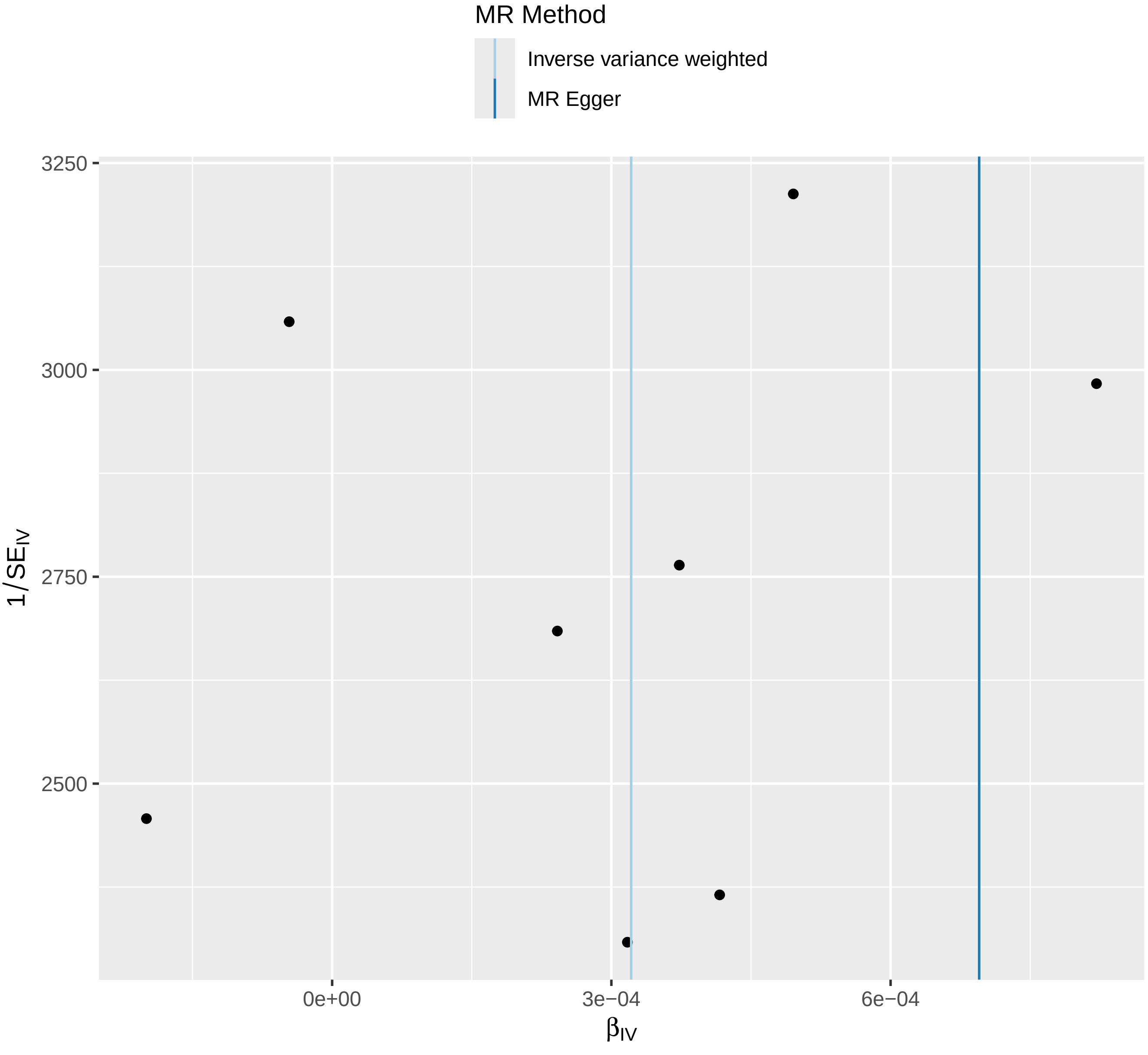

MR Method
Inverse variance weighted
MR Egger
3250
3000
2750
2500
$1/SE_{IV}$
0e+00
3e−04
6e−04
β_{IV}

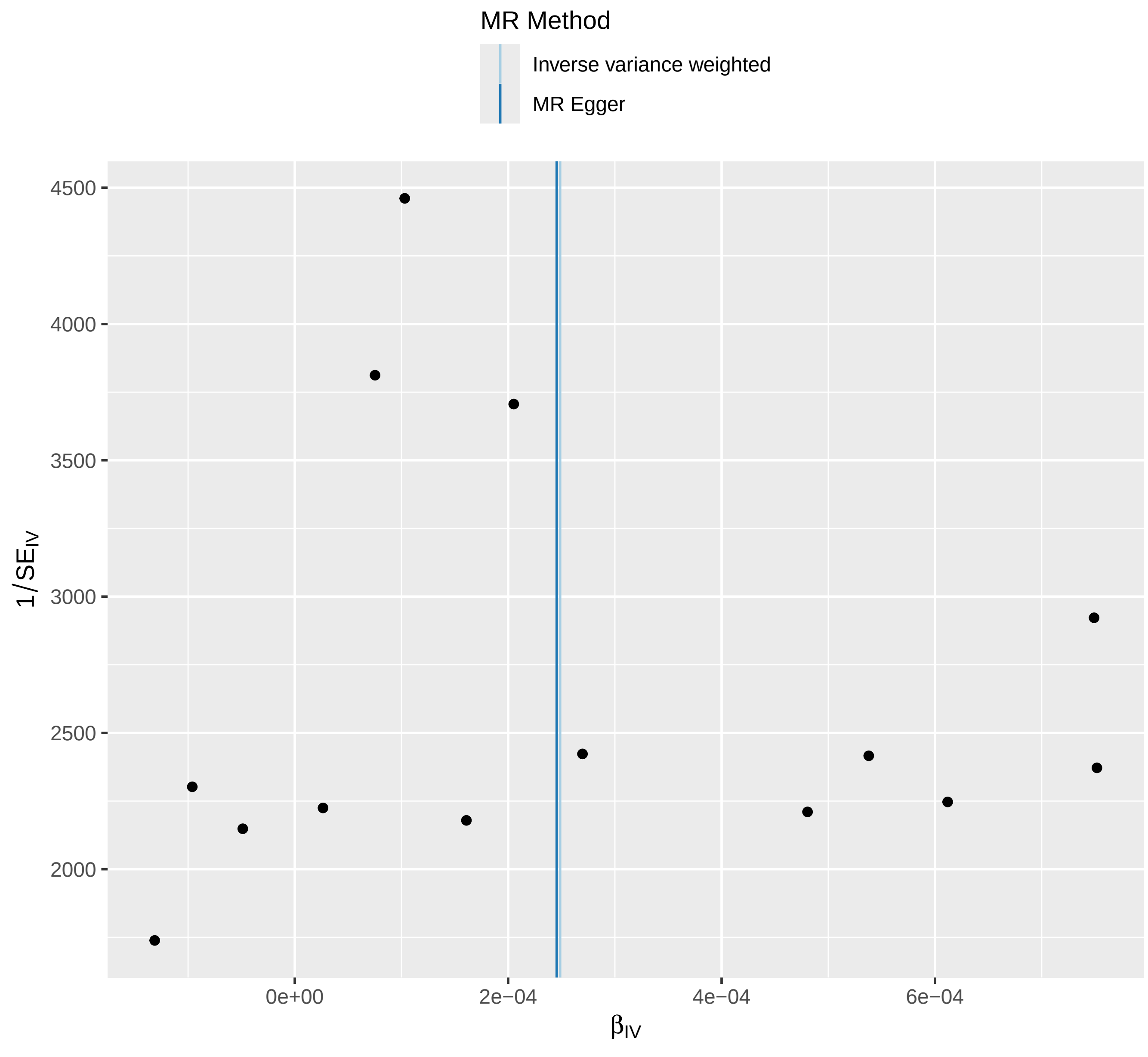

MR Method
Inverse variance weighted
MR Egger
4500
4000
3500
3000
2500
2000
$1/SE_{IV}$
0e+00
2e−04
4e−04
6e−04
β_{IV}

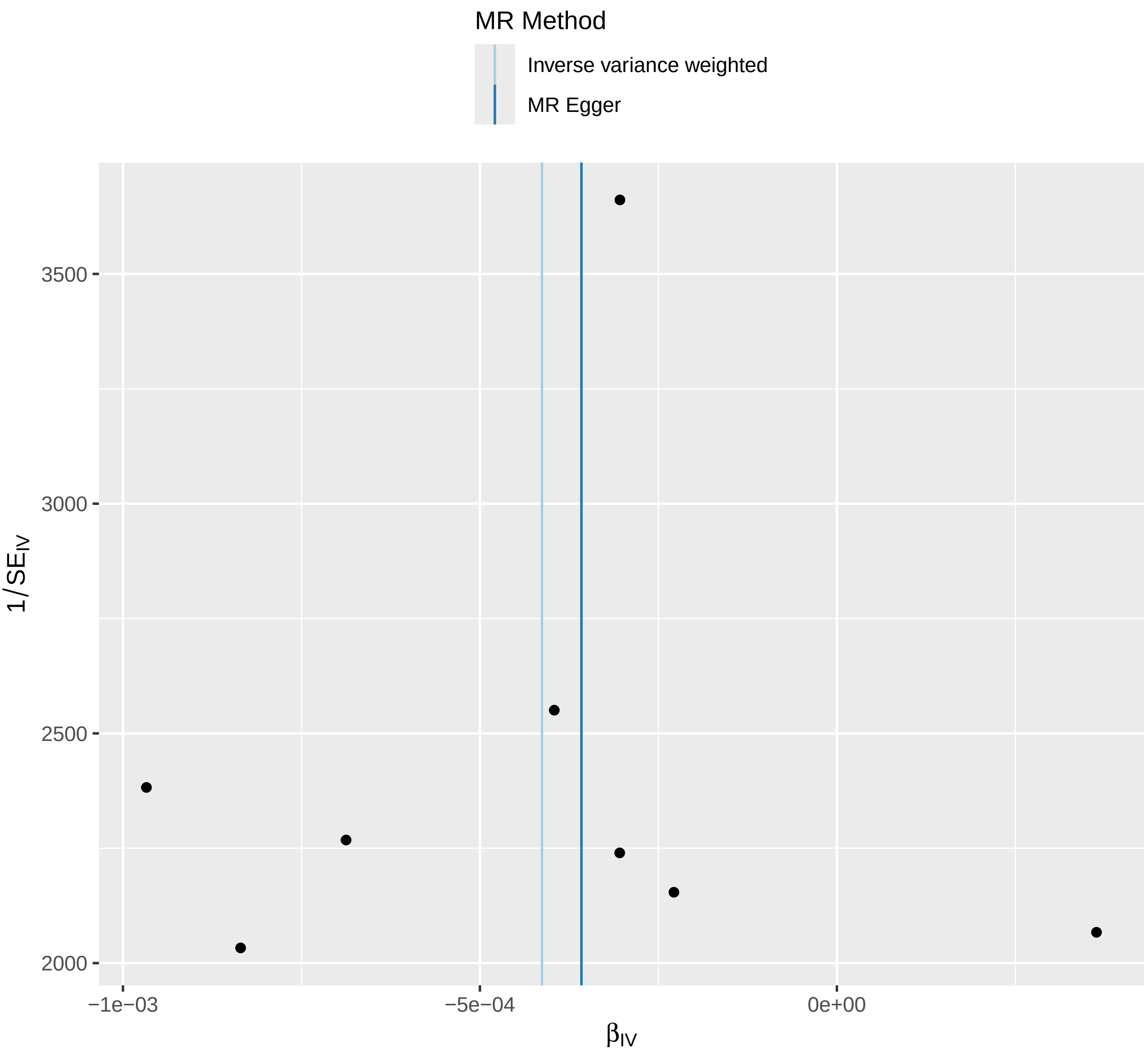

MR Method
Inverse variance weighted
MR Egger
3500
3000
2500
2000
1/SE_IV
−1e−03
−5e−04
0e+00
β_IV

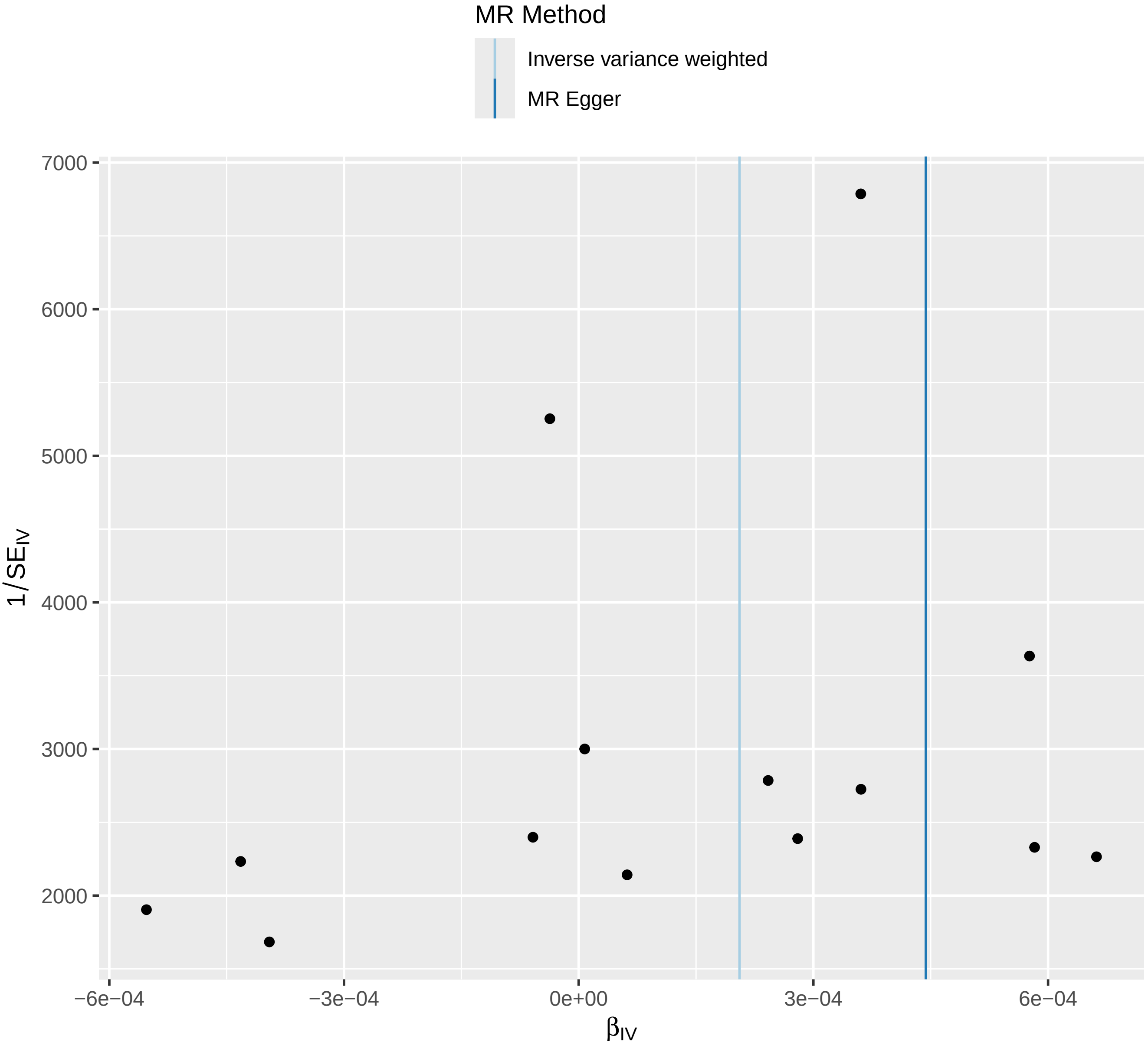
MR Method
Inverse variance weighted
MR Egger
7000
6000
5000
4000
3000
2000
1/SE_IV
−6e−04
−3e−04
0e+00
3e−04
6e−04
β_IV

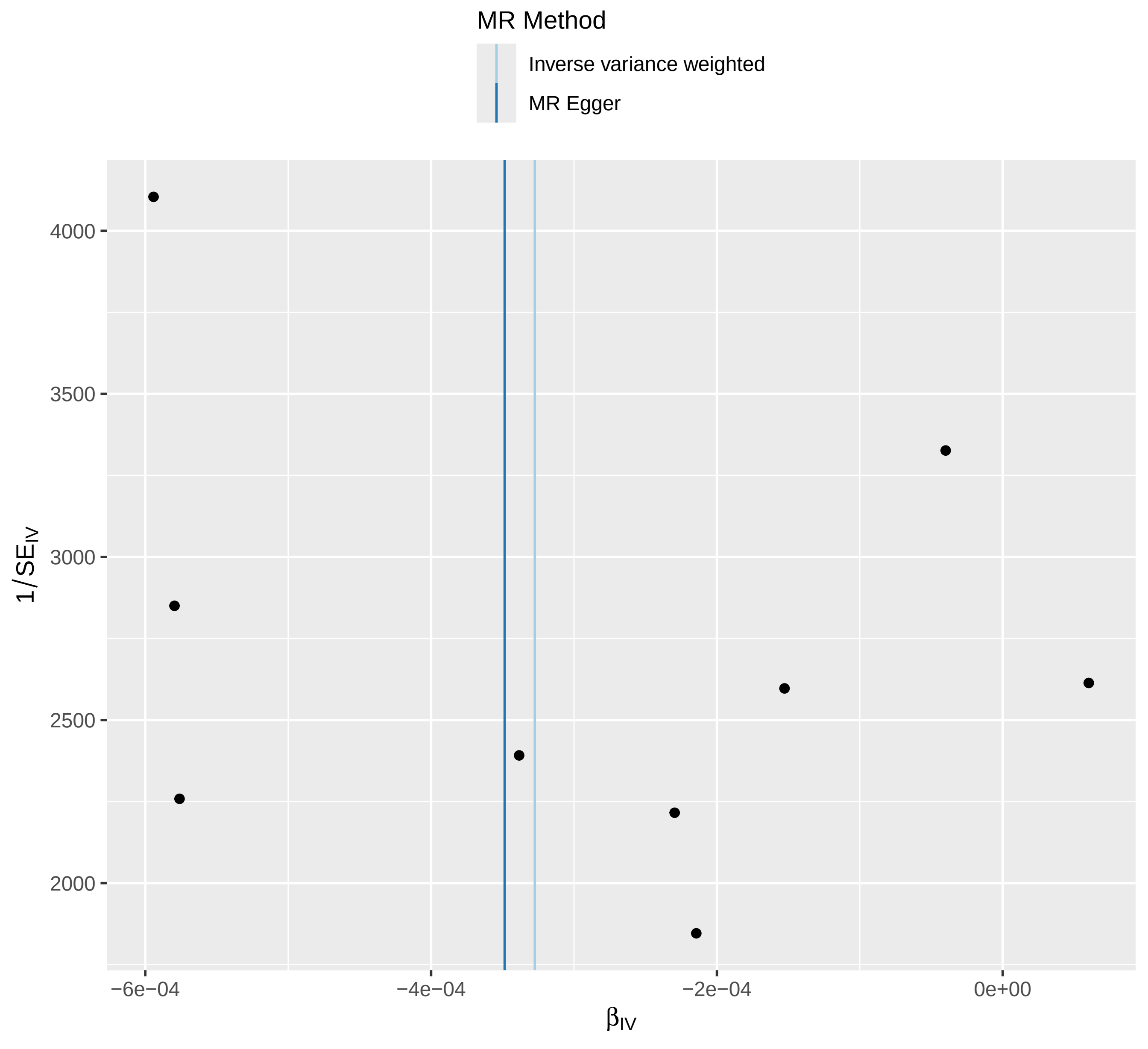

MR Method
Inverse variance weighted
MR Egger
4000
3500
3000
2500
2000
1/SE$_{IV}$
−6e−04
−4e−04
−2e−04
0e+00
β$_{IV}$

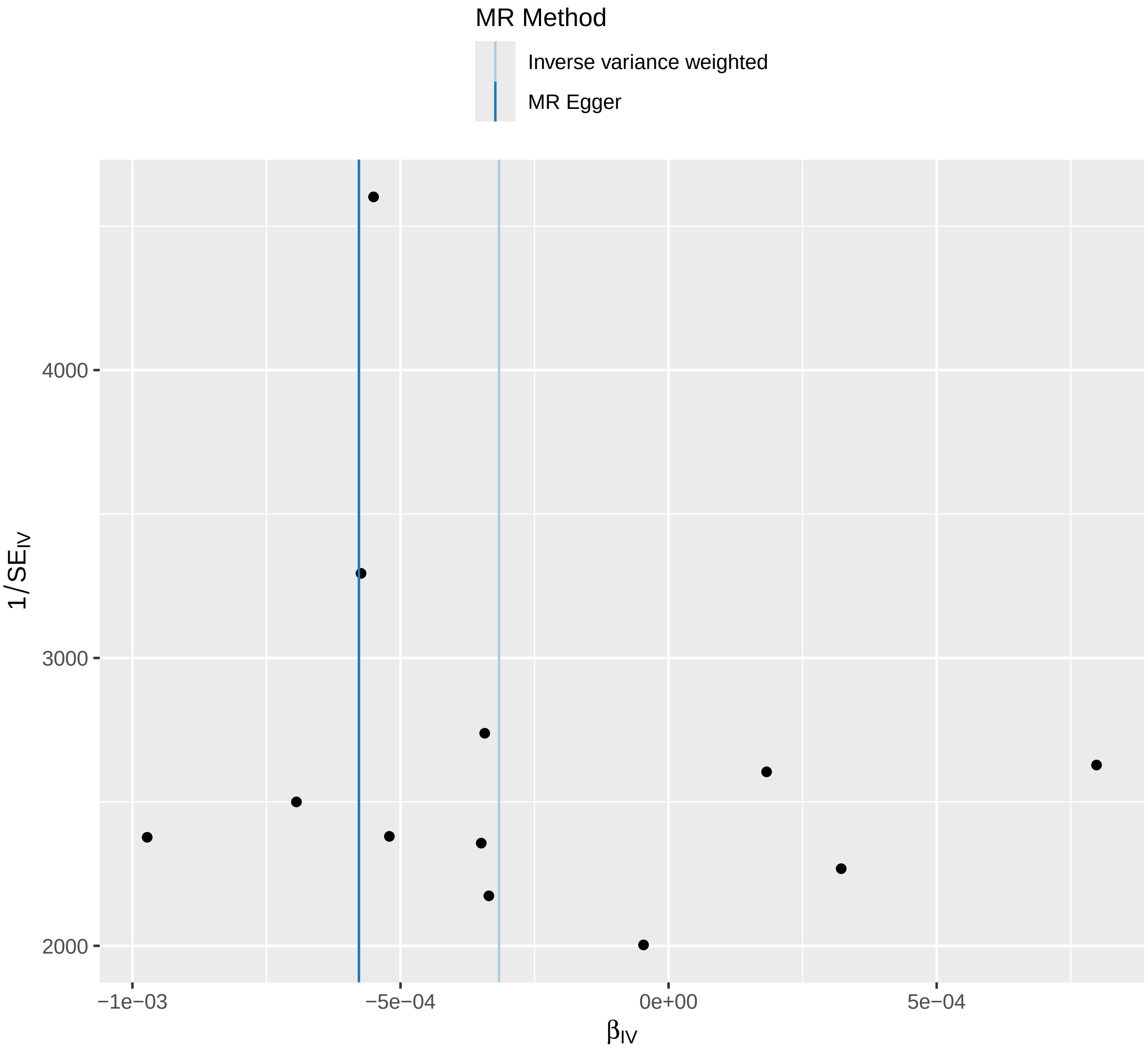

MR Method
Inverse variance weighted
MR Egger
1/SE_IV
4000
3000
2000
−1e−03
−5e−04
0e+00
5e−04
β_IV

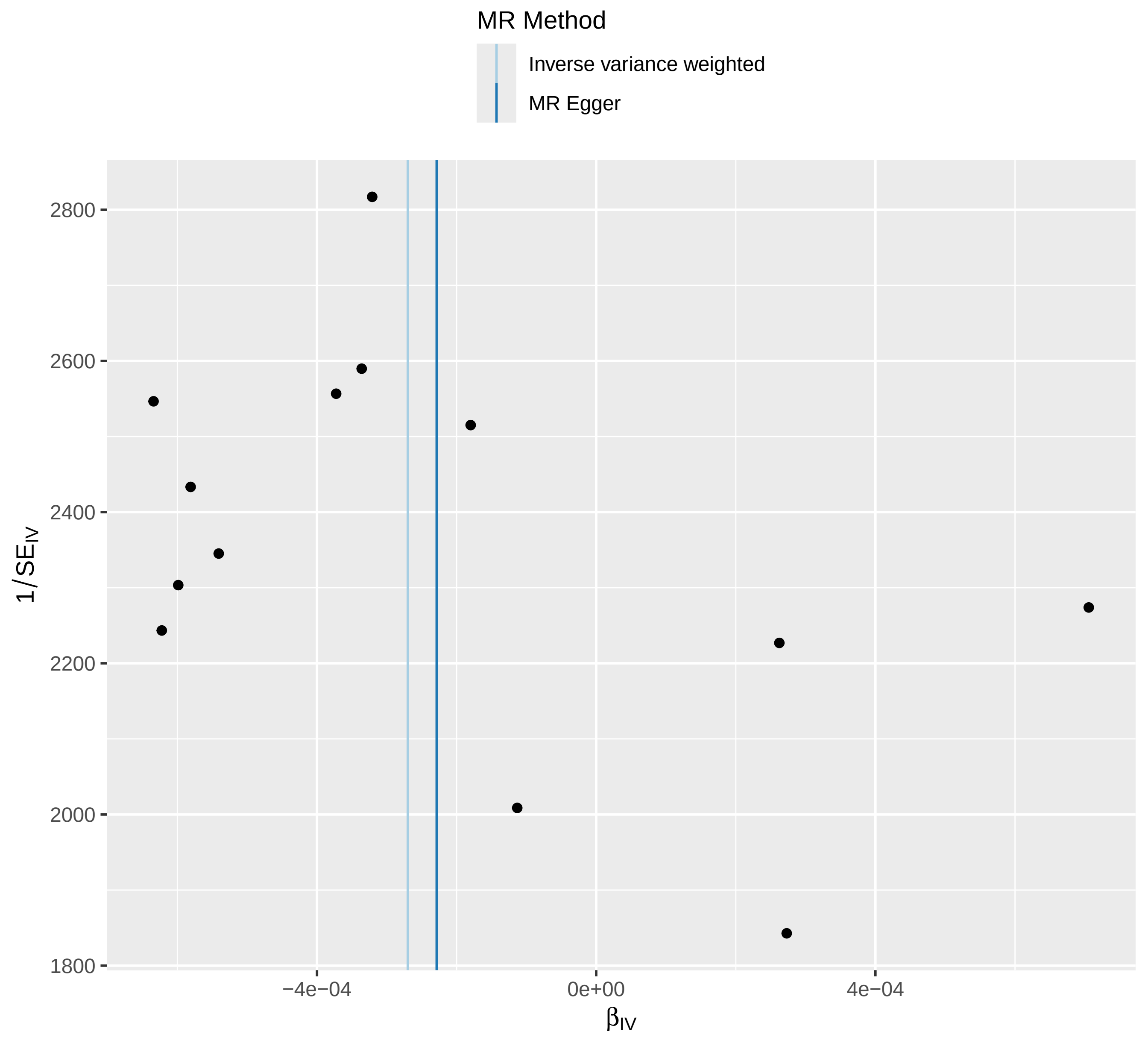

MR Method
Inverse variance weighted
MR Egger
2800
2600
2400
2200
2000
1800
$1/SE_{IV}$
−4e−04
0e+00
4e−04
β_{IV}

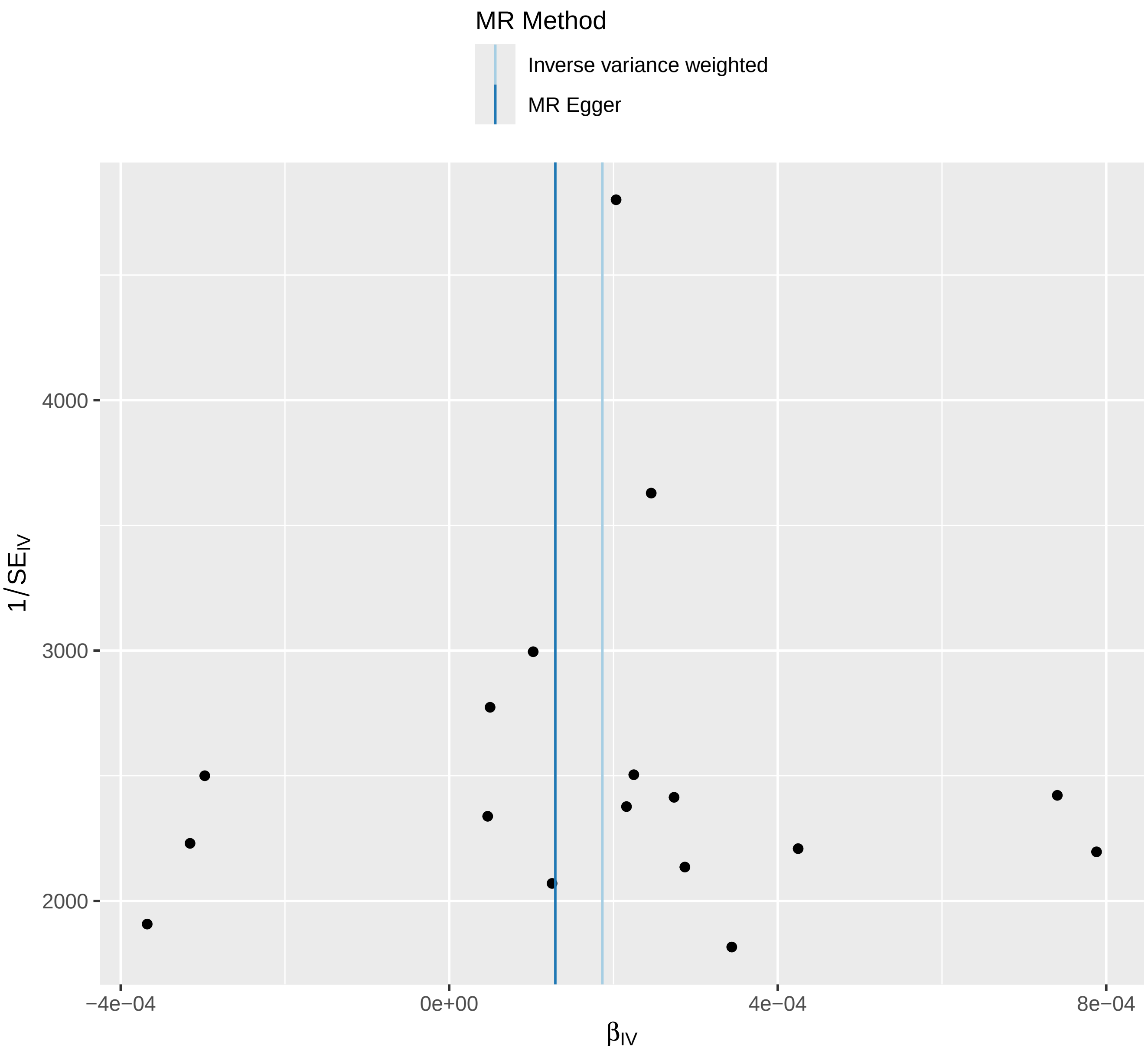
MR Method
Inverse variance weighted
MR Egger
4000
3000
2000
1/SE_IV
−4e−04
0e+00
4e−04
8e−04
β_IV

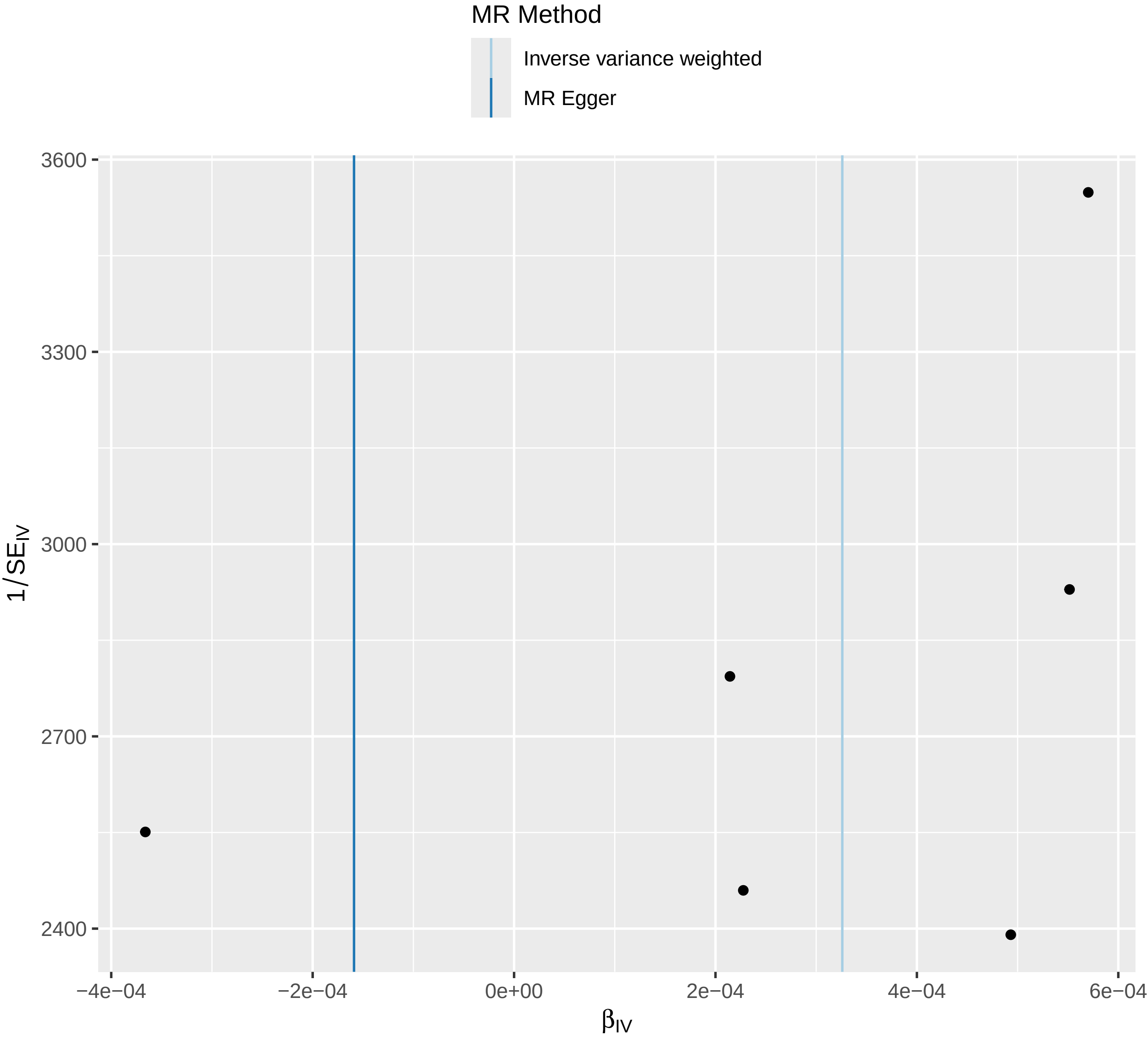
MR Method
Inverse variance weighted
MR Egger
3600
3300
3000
2700
2400
1/SE_IV
−4e−04
−2e−04
0e+00
2e−04
4e−04
6e−04
β_IV

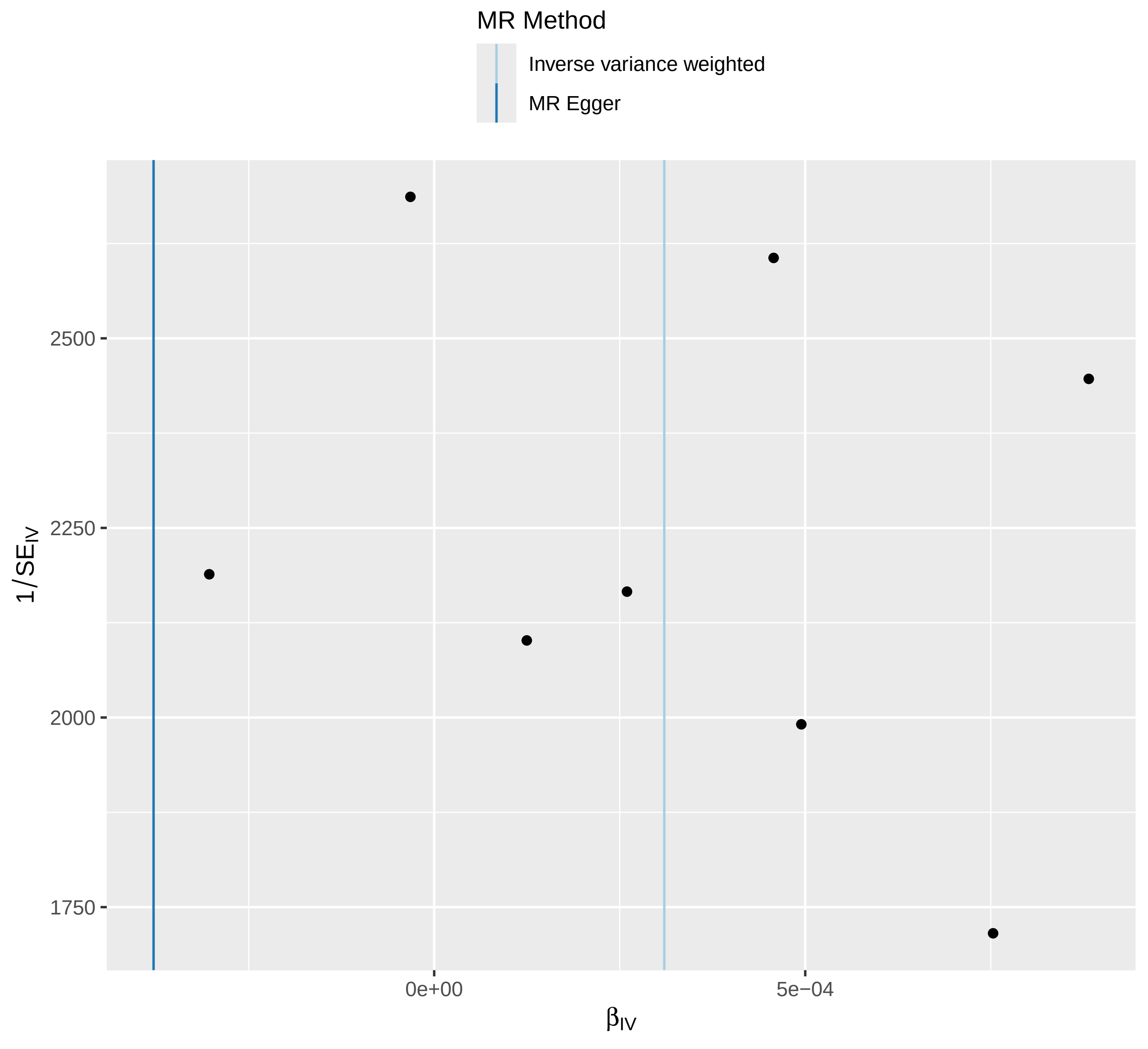
MR Method
Inverse variance weighted
MR Egger
2500
2250
2000
1750
1/SE_IV
0e+00
5e−04
β_IV

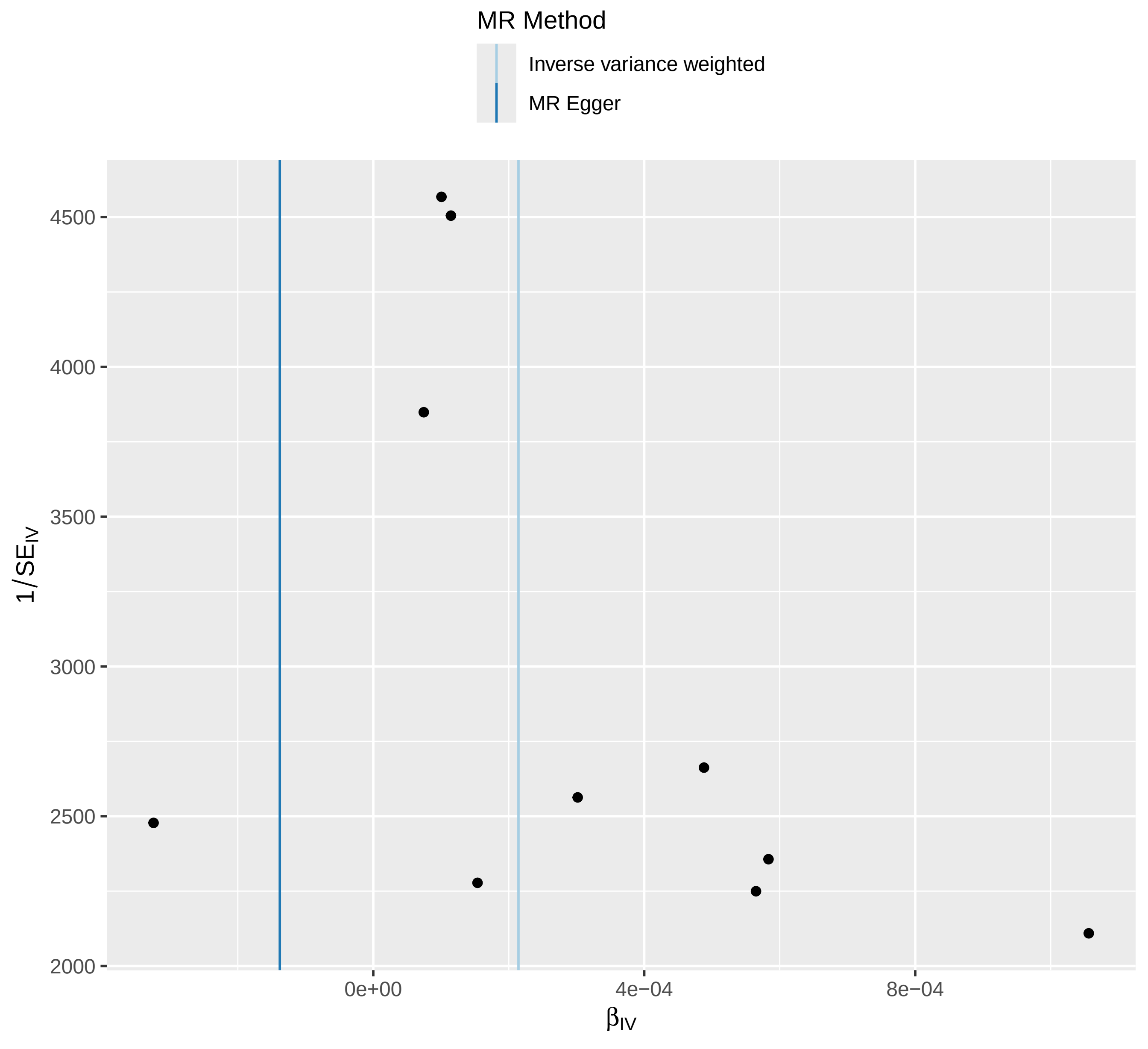
MR Method
Inverse variance weighted
MR Egger
4500
4000
3500
3000
2500
2000
1/SE_IV
0e+00
4e−04
8e−04
β_IV

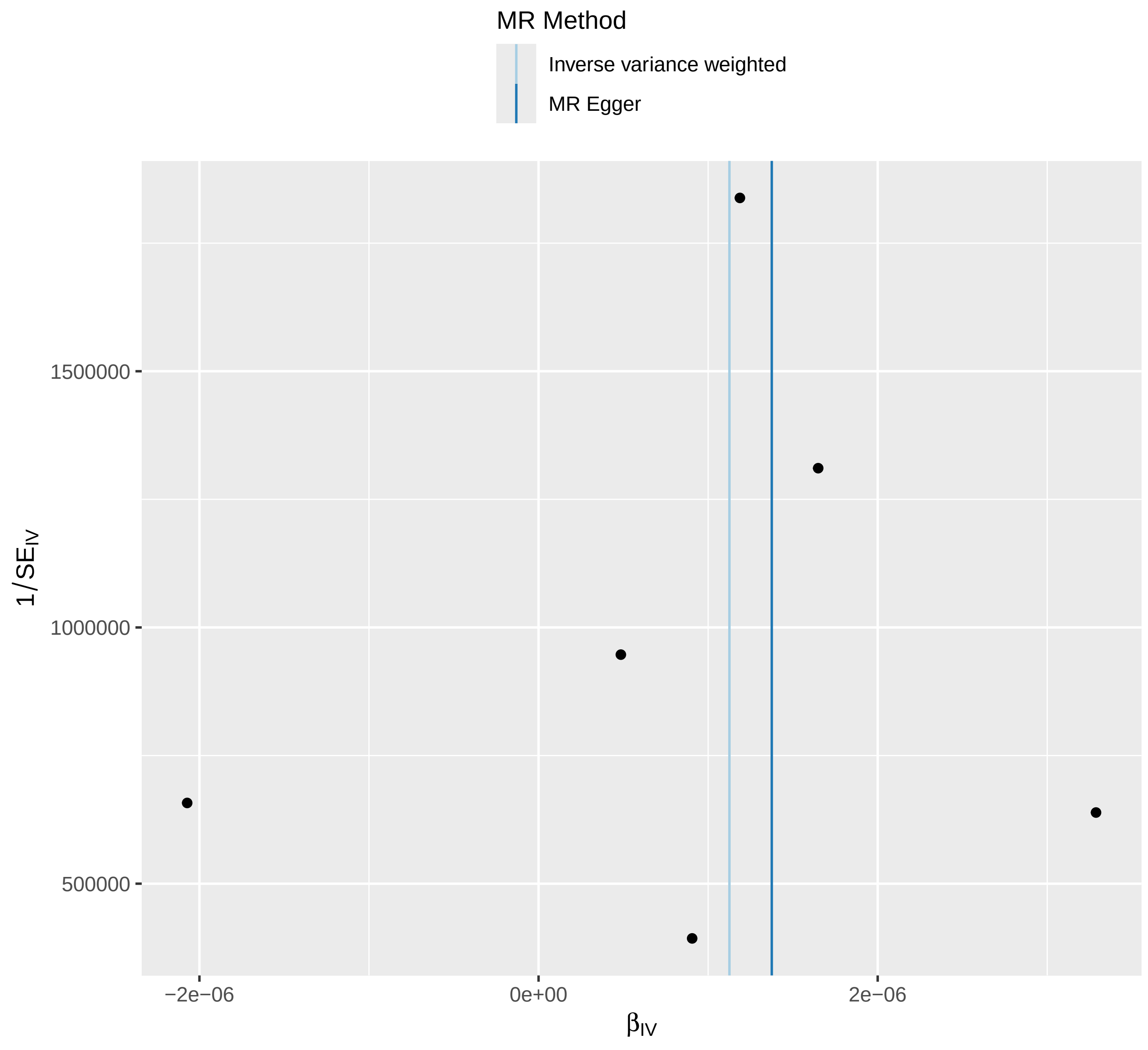
MR Method
Inverse variance weighted
MR Egger
1500000
1000000
500000
$1/SE_{IV}$
−2e−06
0e+00
2e−06
β_{IV}

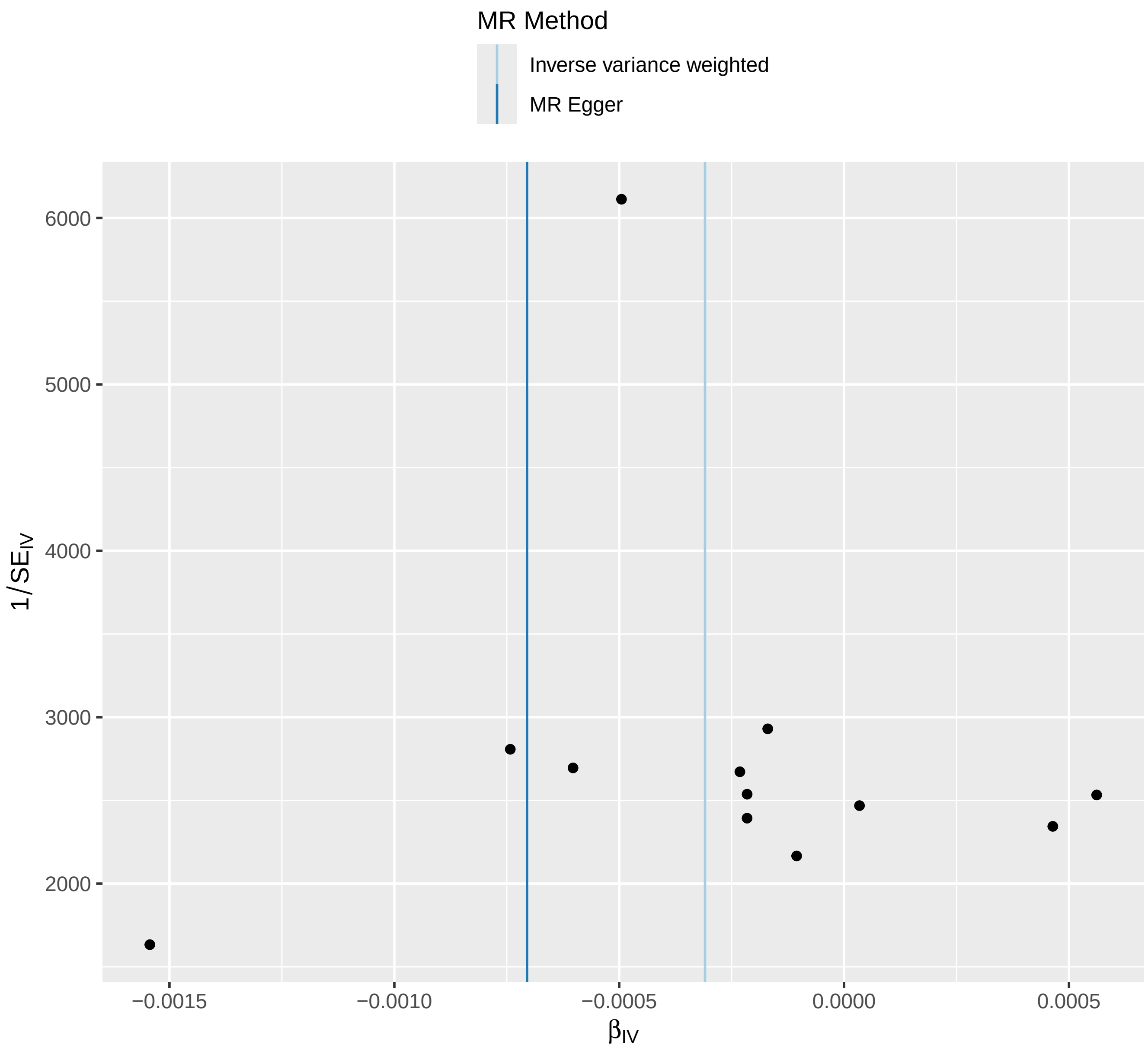

MR Method
Inverse variance weighted
MR Egger
6000
5000
4000
3000
2000
1/SE_IV
−0.0015
−0.0010
−0.0005
0.0000
0.0005
β_IV

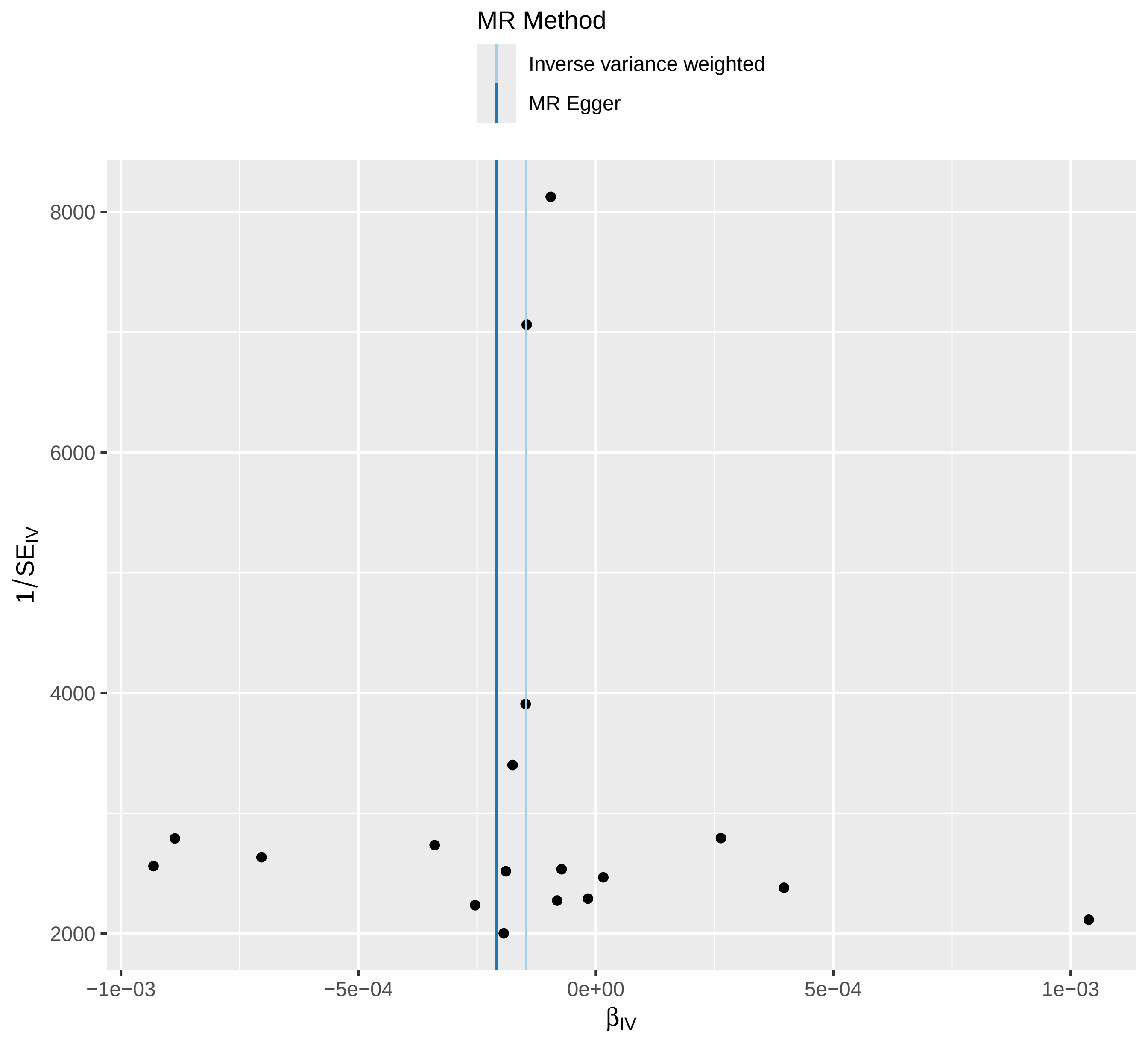

MR Method
Inverse variance weighted
MR Egger
8000
6000
4000
2000
1/SE_IV
−1e−03
−5e−04
0e+00
5e−04
1e−03
β_IV

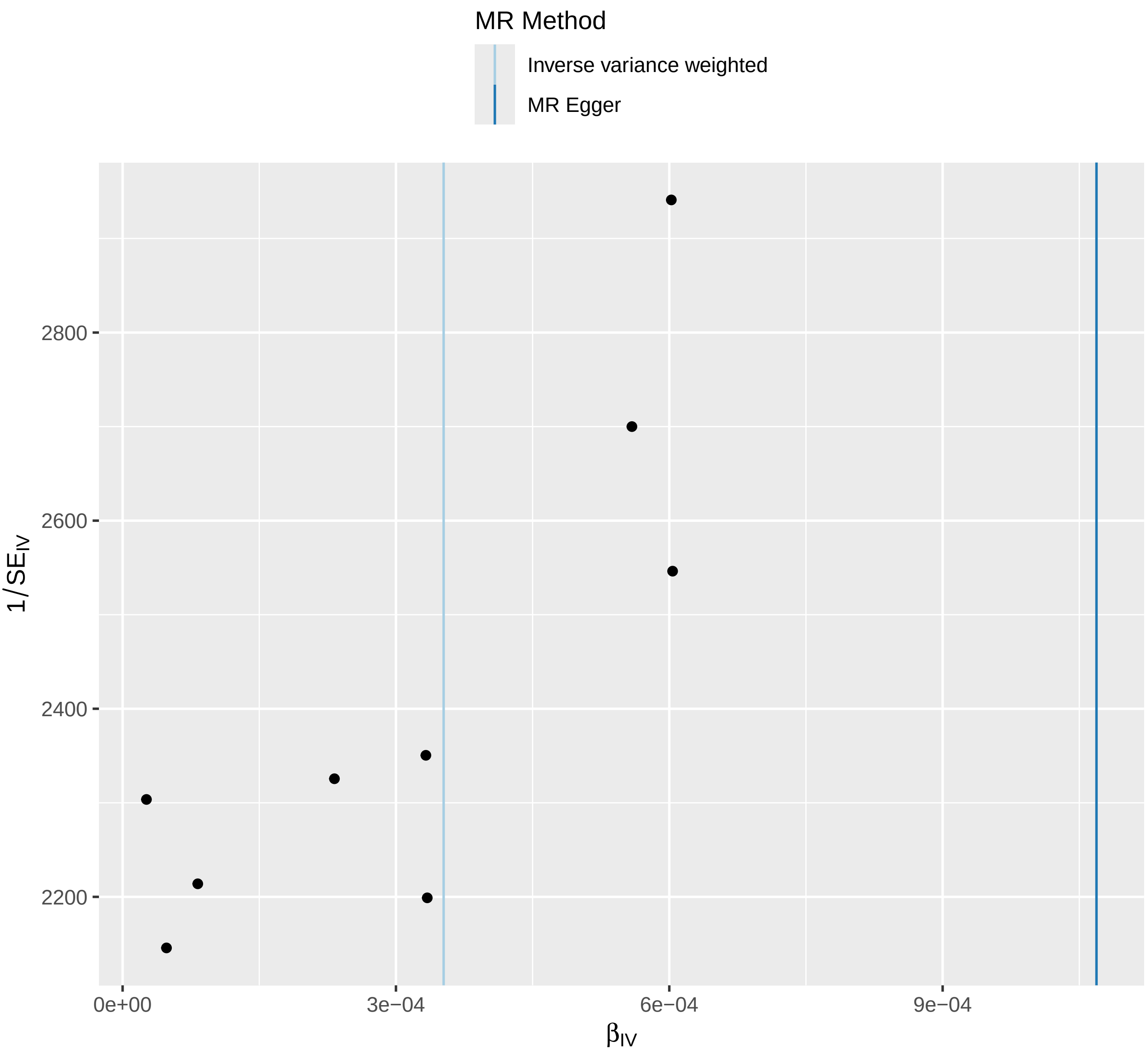

MR Method
Inverse variance weighted
MR Egger
2800
2600
2400
2200
1/SE_IV
0e+00
3e−04
6e−04
9e−04
β_IV

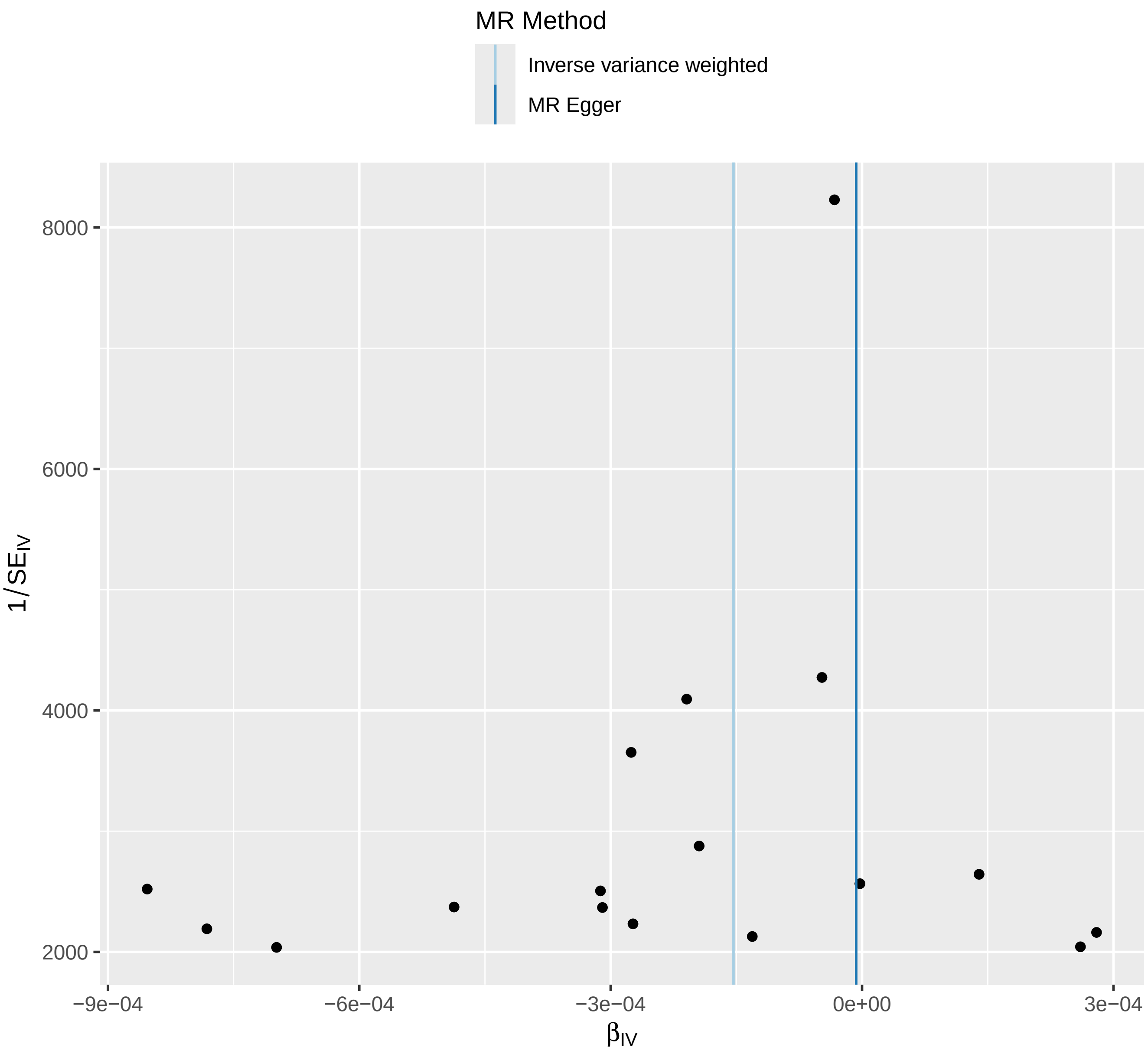
MR Method
Inverse variance weighted
MR Egger
8000
6000
4000
2000
1/SE_IV
−9e−04
−6e−04
−3e−04
0e+00
3e−04
β_IV

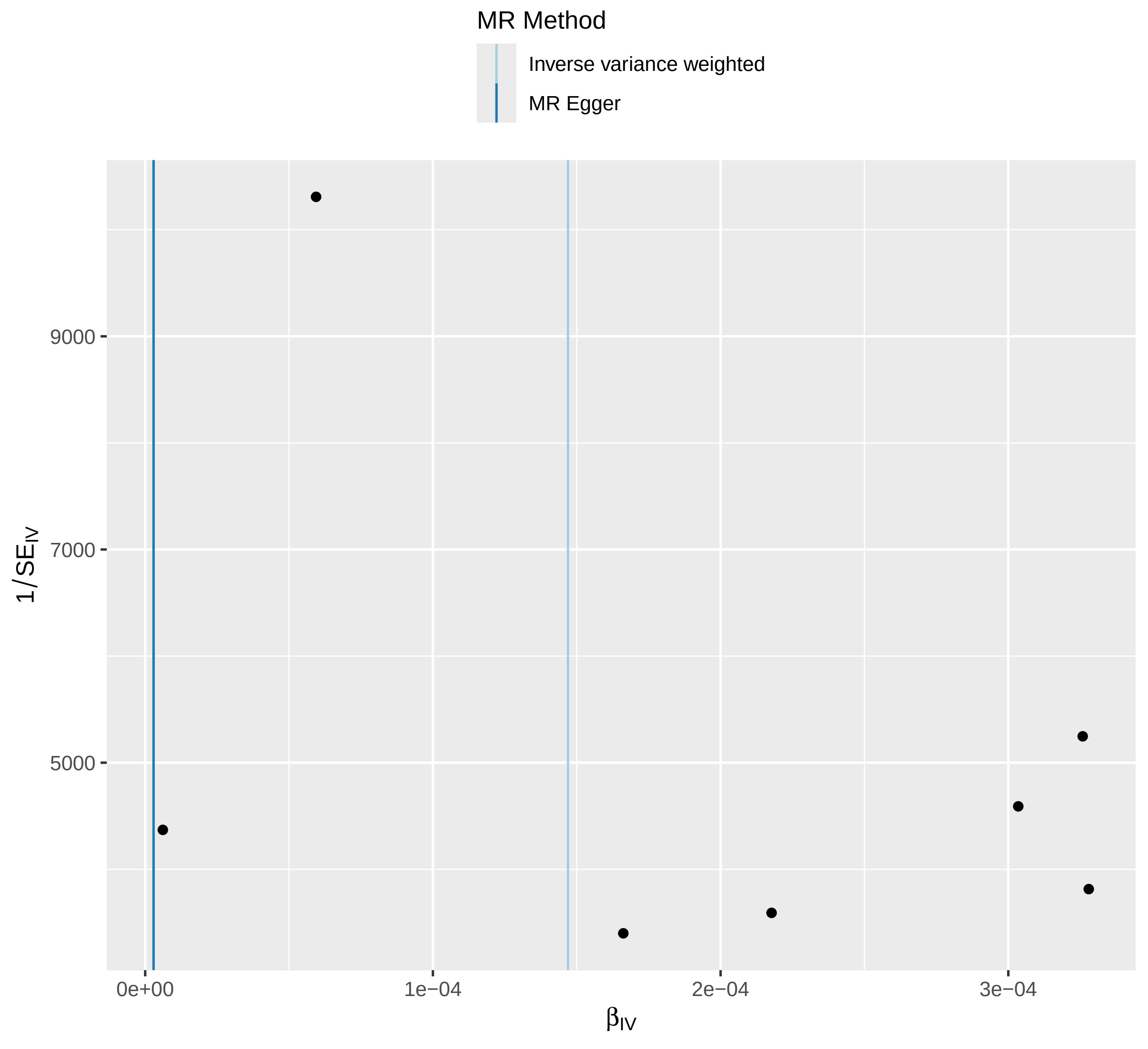

MR Method
Inverse variance weighted
MR Egger
9000
7000
5000
$1/SE_{IV}$
0e+00
1e−04
2e−04
3e−04
β_{IV}

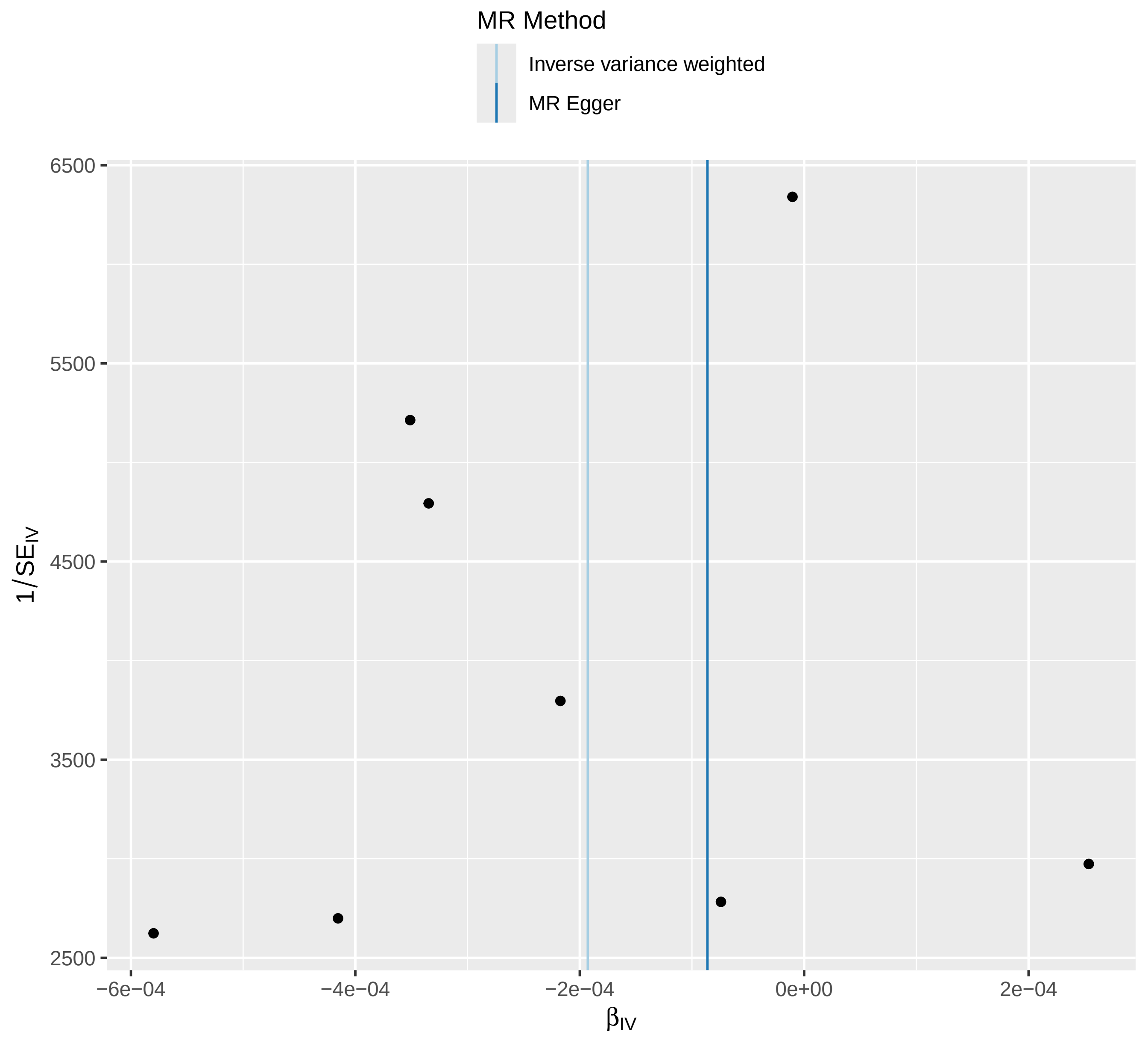

MR Method
Inverse variance weighted
MR Egger
6500
5500
4500
3500
2500
1/SE_IV
−6e−04
−4e−04
−2e−04
0e+00
2e−04
β_IV

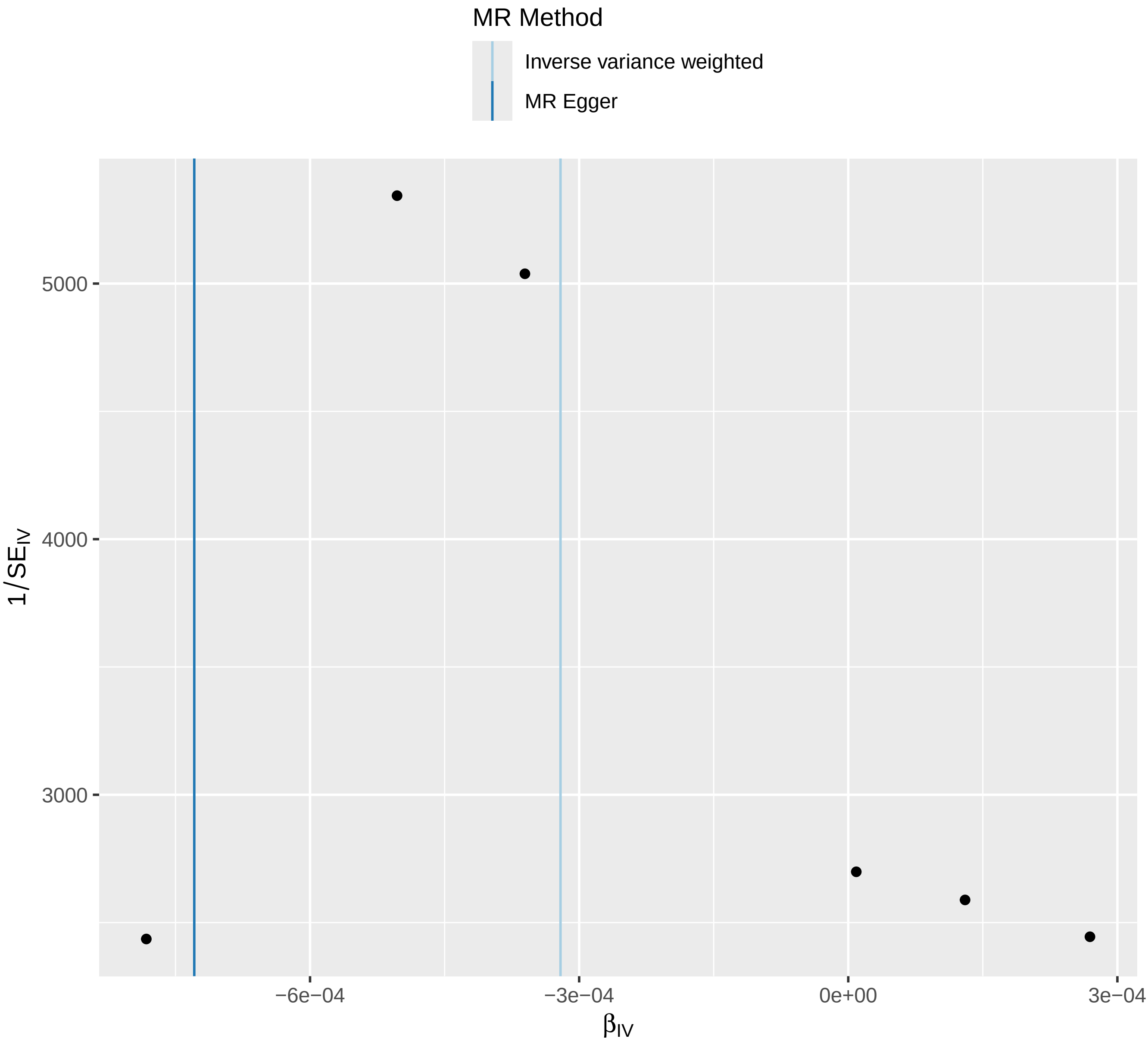

MR Method
Inverse variance weighted
MR Egger
5000
4000
3000
1/SE$_{IV}$
−6e−04
−3e−04
0e+00
3e−04
β$_{IV}$

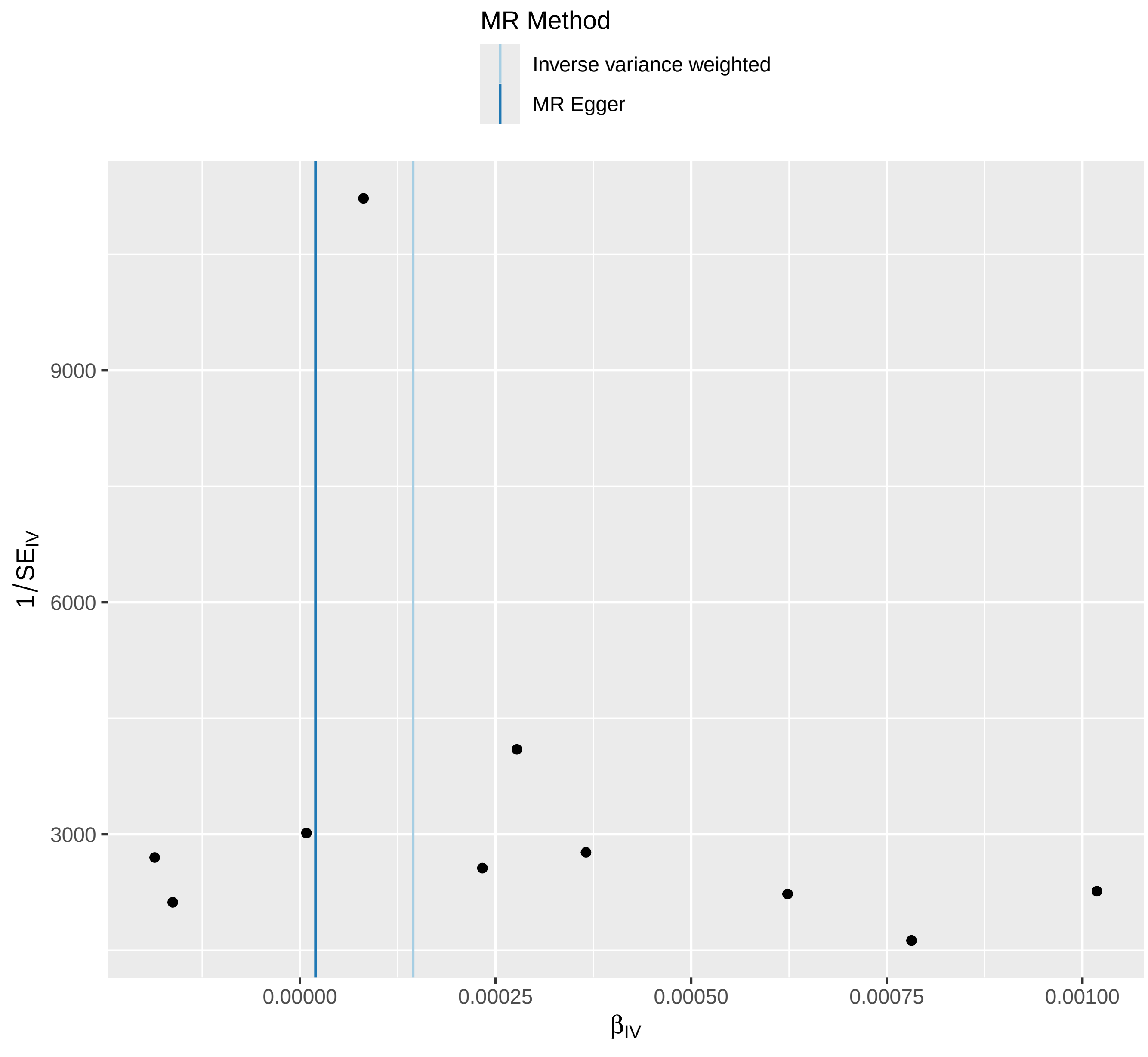
MR Method
Inverse variance weighted
MR Egger
9000
6000
3000
1/SE_IV
0.00000
0.00025
0.00050
0.00075
0.00100
β_IV

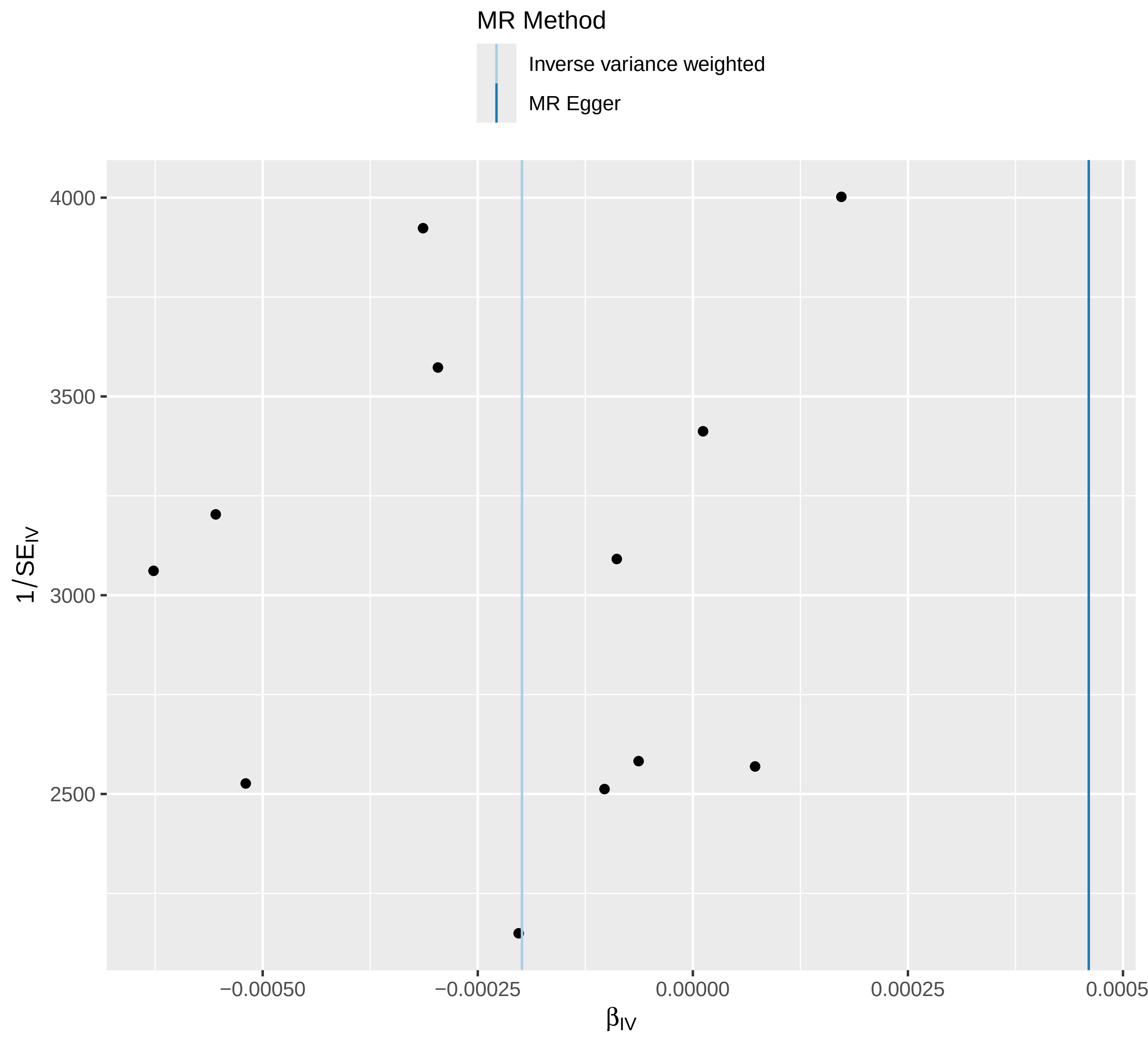

MR Method
Inverse variance weighted
MR Egger
4000
3500
3000
2500
$1/SE_{IV}$
−0.00050
−0.00025
0.00000
0.00025
0.0005
β_{IV}

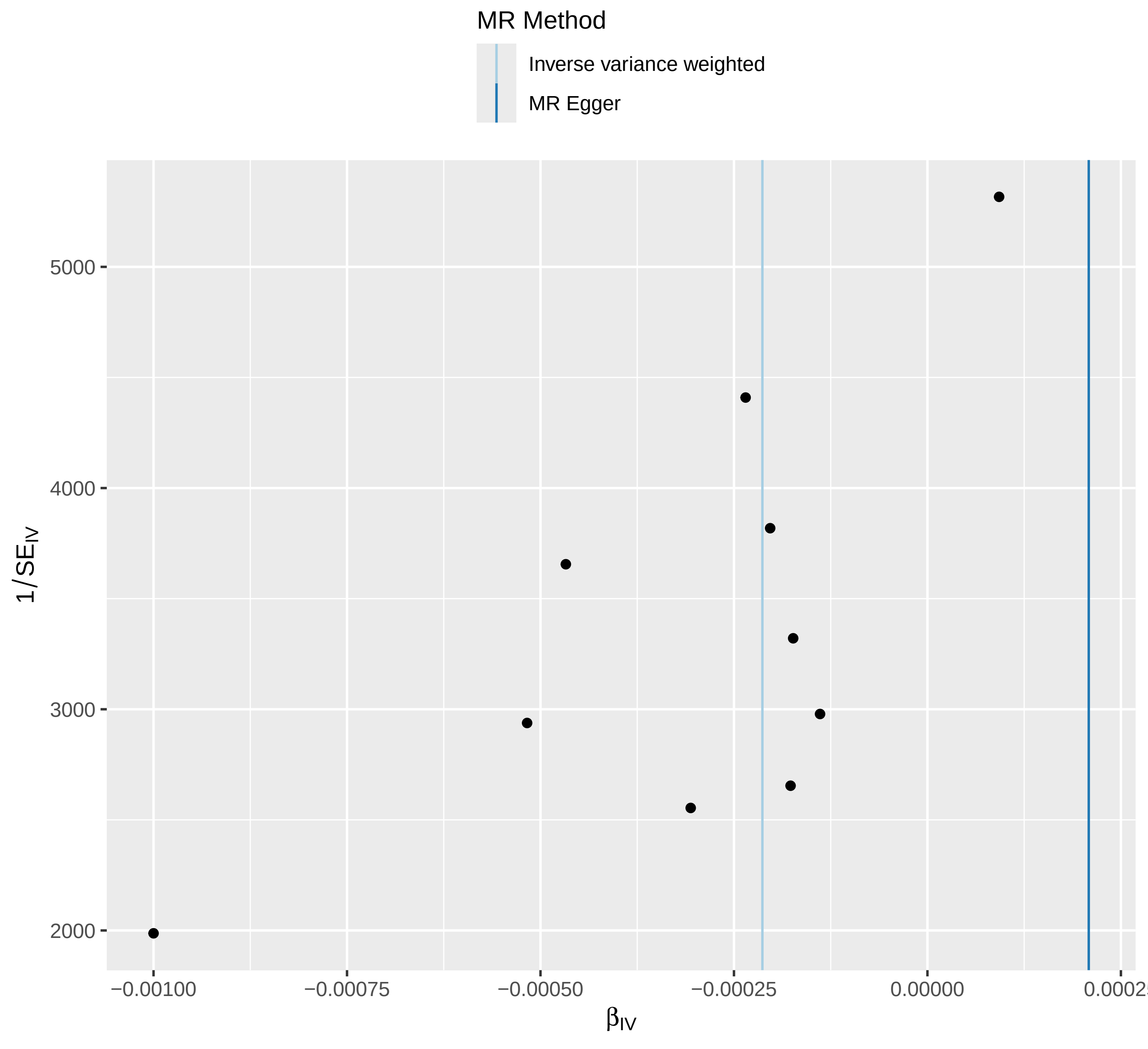
MR Method
Inverse variance weighted
MR Egger
5000
4000
3000
2000
1/SE_IV
−0.00100
−0.00075
−0.00050
−0.00025
0.00000
0.00025
β_IV

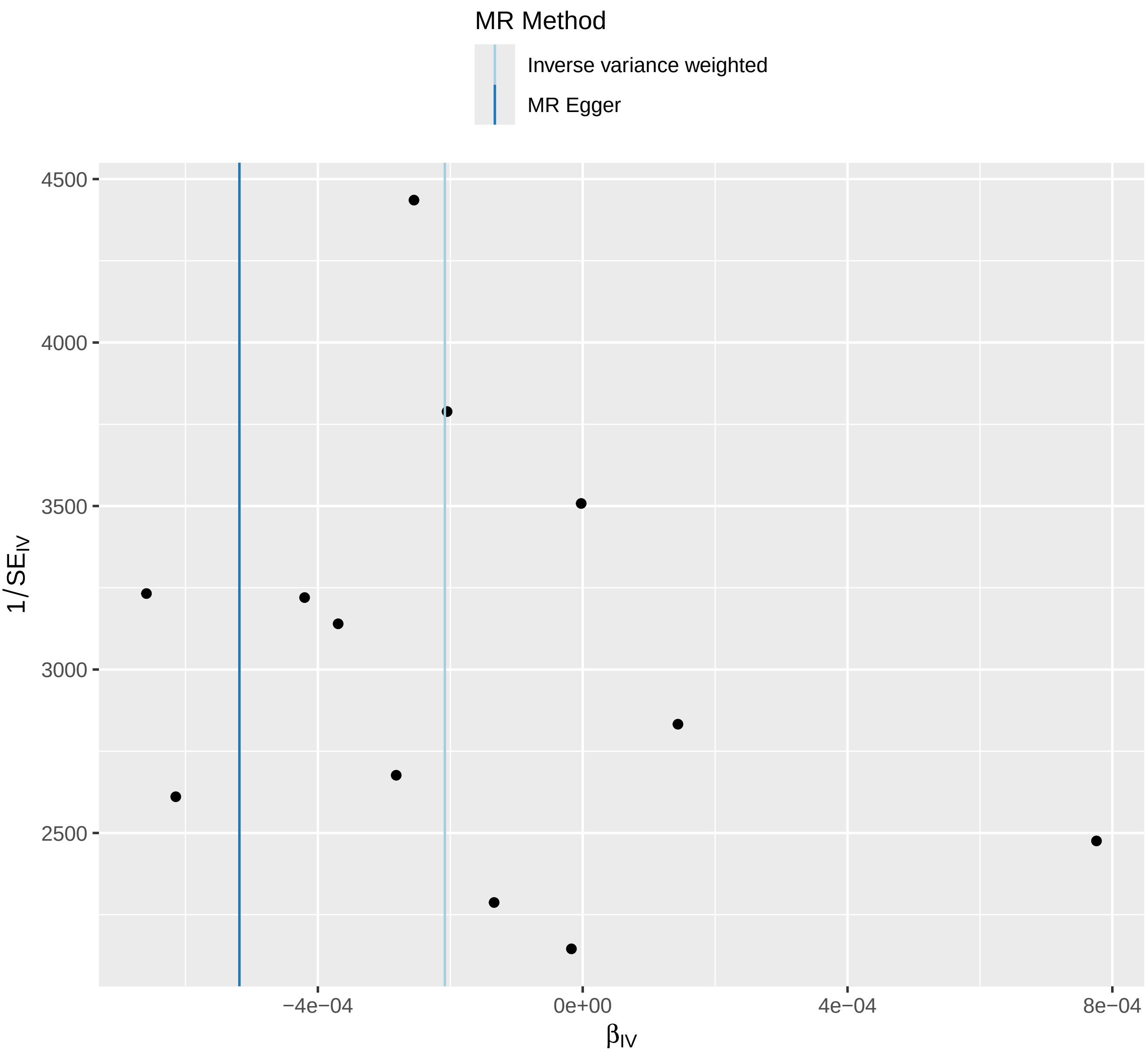

MR Method
Inverse variance weighted
MR Egger
4500
4000
3500
3000
2500
1/SE_IV
−4e−04
0e+00
4e−04
8e−04
β_IV

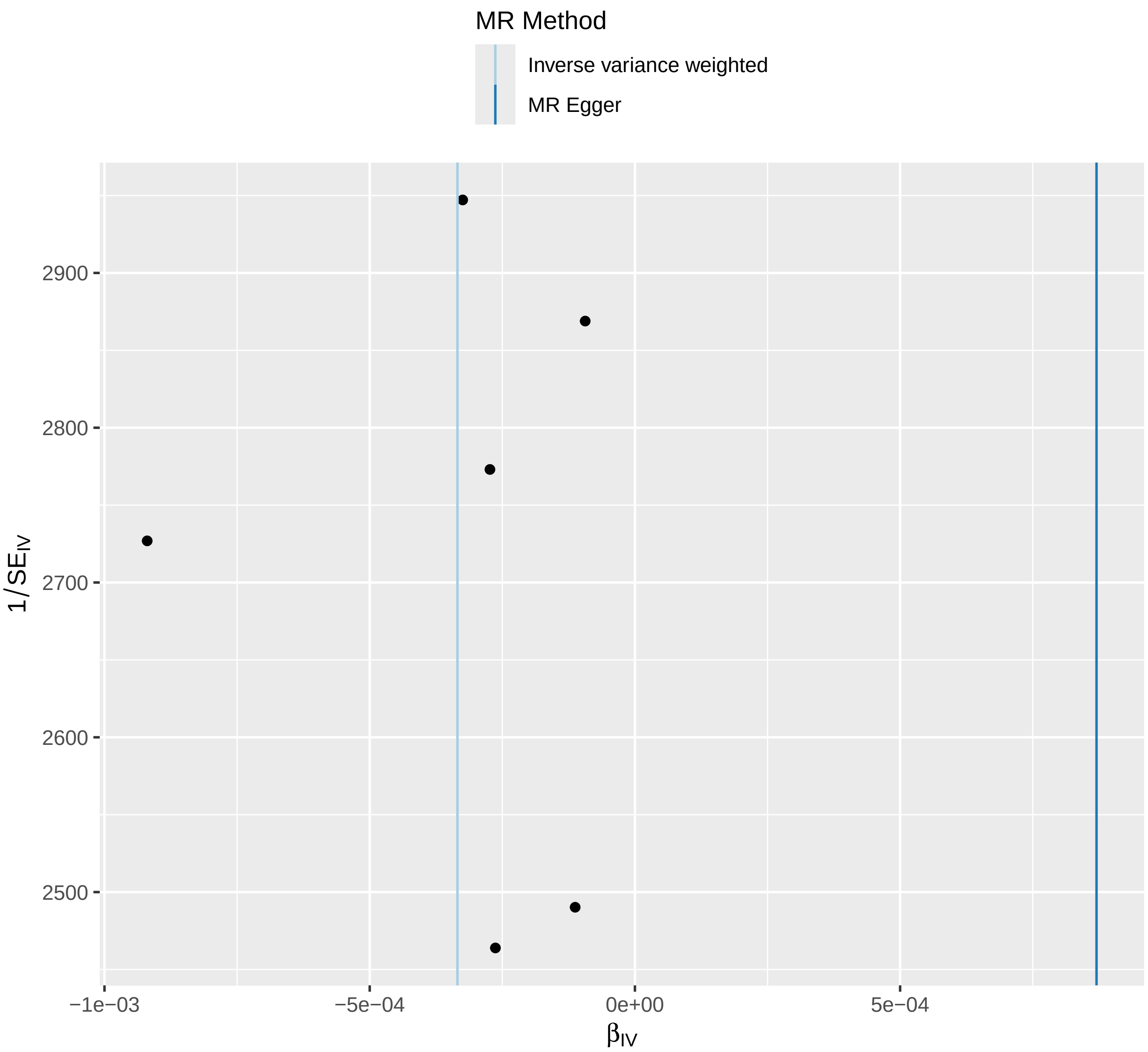

MR Method
Inverse variance weighted
MR Egger
2900
2800
2700
2600
2500
1/SE_IV
−1e−03
−5e−04
0e+00
5e−04
β_IV

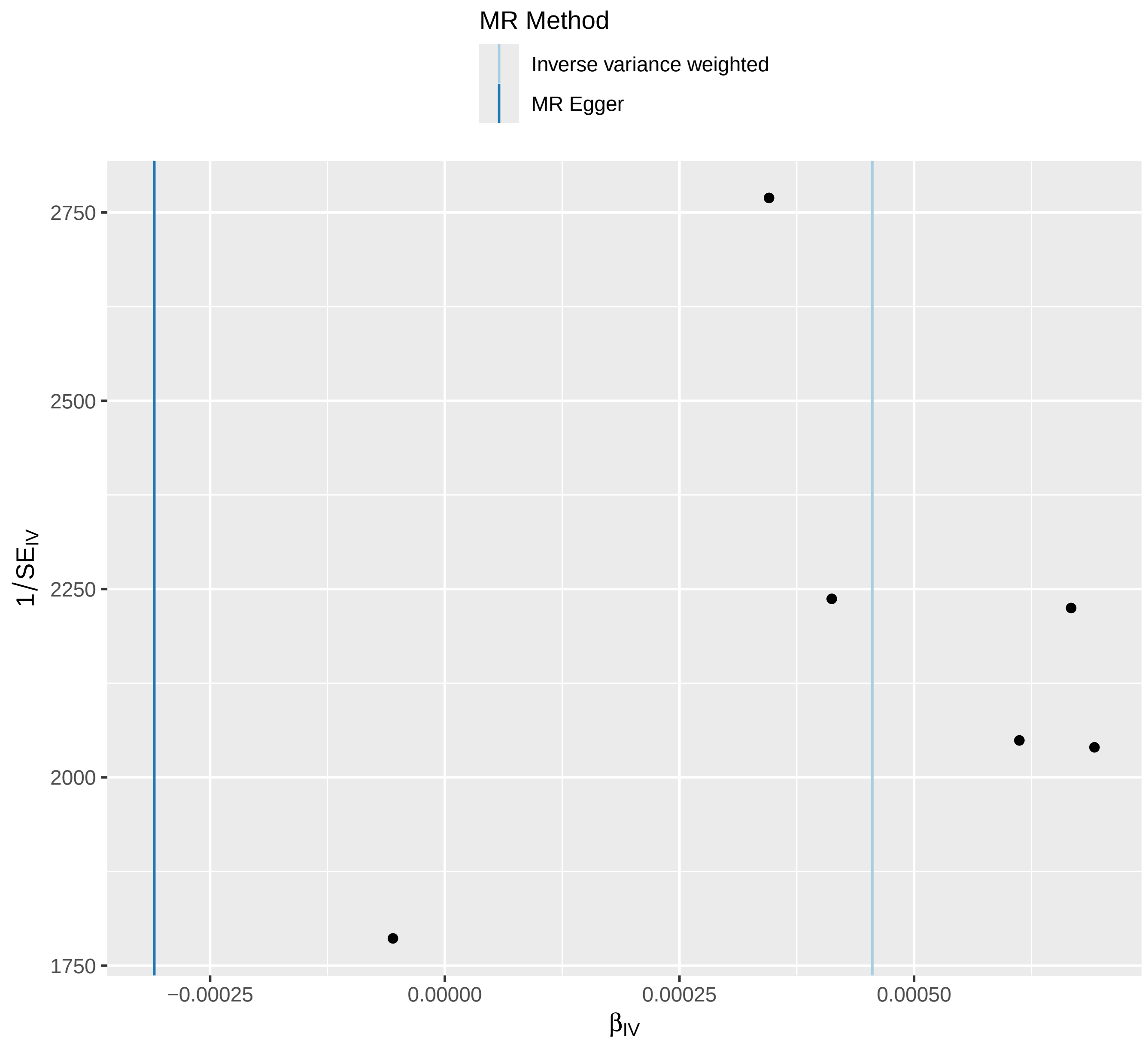

MR Method
Inverse variance weighted
MR Egger
2750
2500
2250
2000
1750
$1/SE_{IV}$
−0.00025
0.00000
0.00025
0.00050
β_{IV}

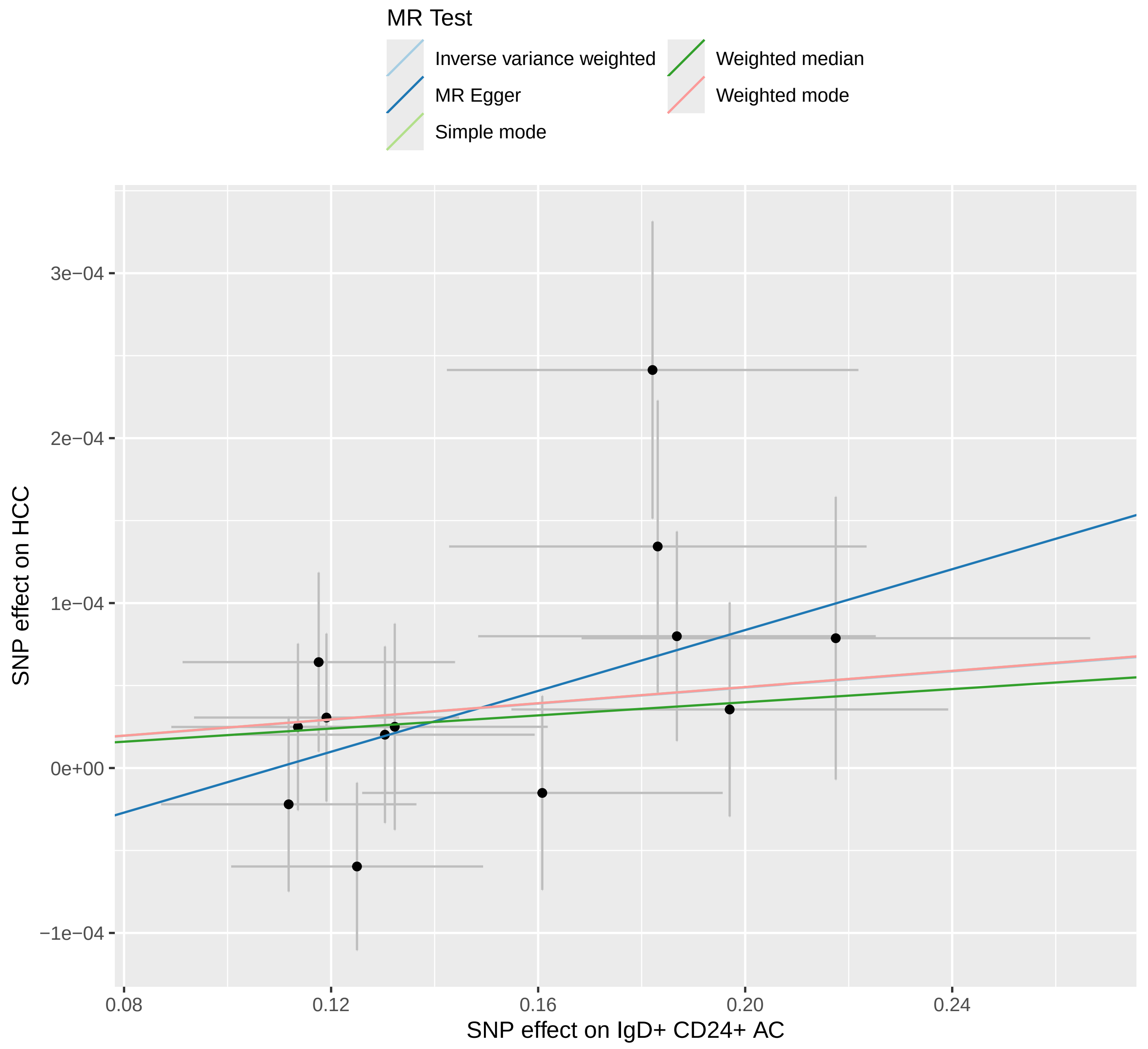
MR Test
Inverse variance weighted
MR Egger
Simple mode
Weighted median
Weighted mode
3e−04
2e−04
1e−04
0e+00
−1e−04
SNP effect on HCC
0.08
0.12
0.16
0.20
0.24
SNP effect on IgD+ CD24+ AC

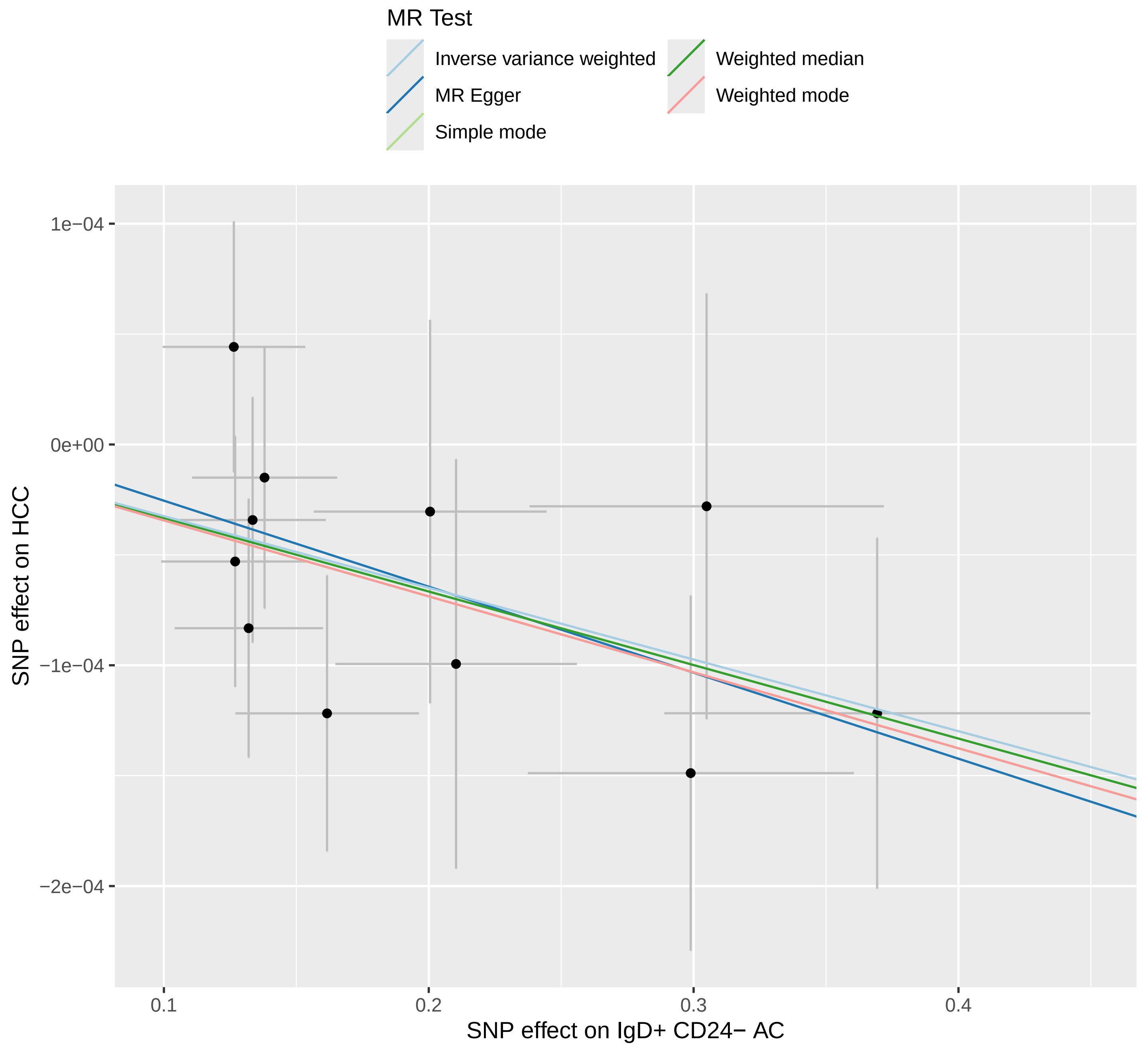
MR Test
Inverse variance weighted
MR Egger
Simple mode
Weighted median
Weighted mode
1e−04
0e+00
−1e−04
−2e−04
SNP effect on HCC
0.1
0.2
0.3
0.4
SNP effect on IgD+ CD24− AC

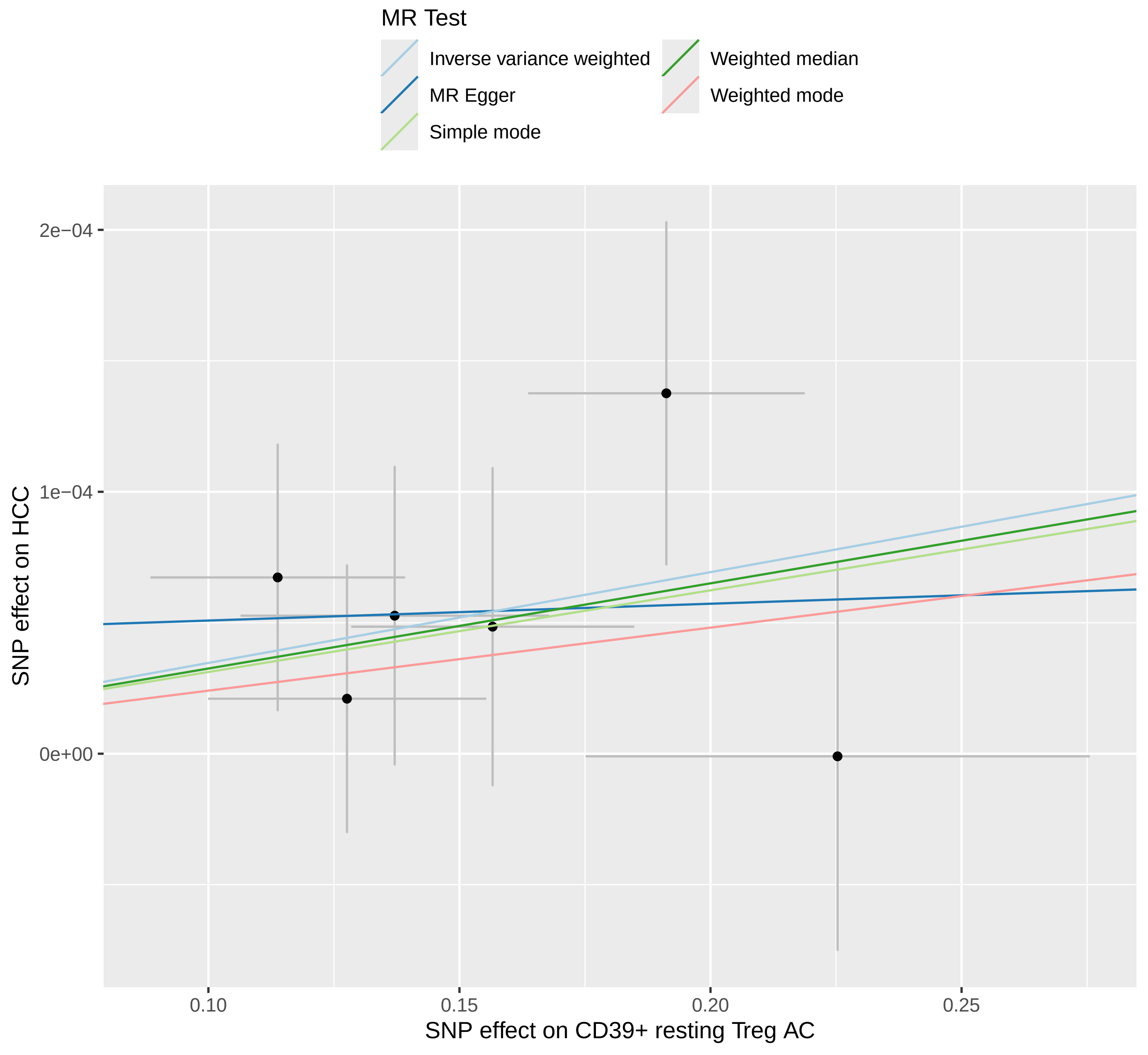

MR Test
Inverse variance weighted
MR Egger
Simple mode
Weighted median
Weighted mode
2e−04
1e−04
0e+00
SNP effect on HCC
0.10
0.15
0.20
0.25
SNP effect on CD39+ resting Treg AC

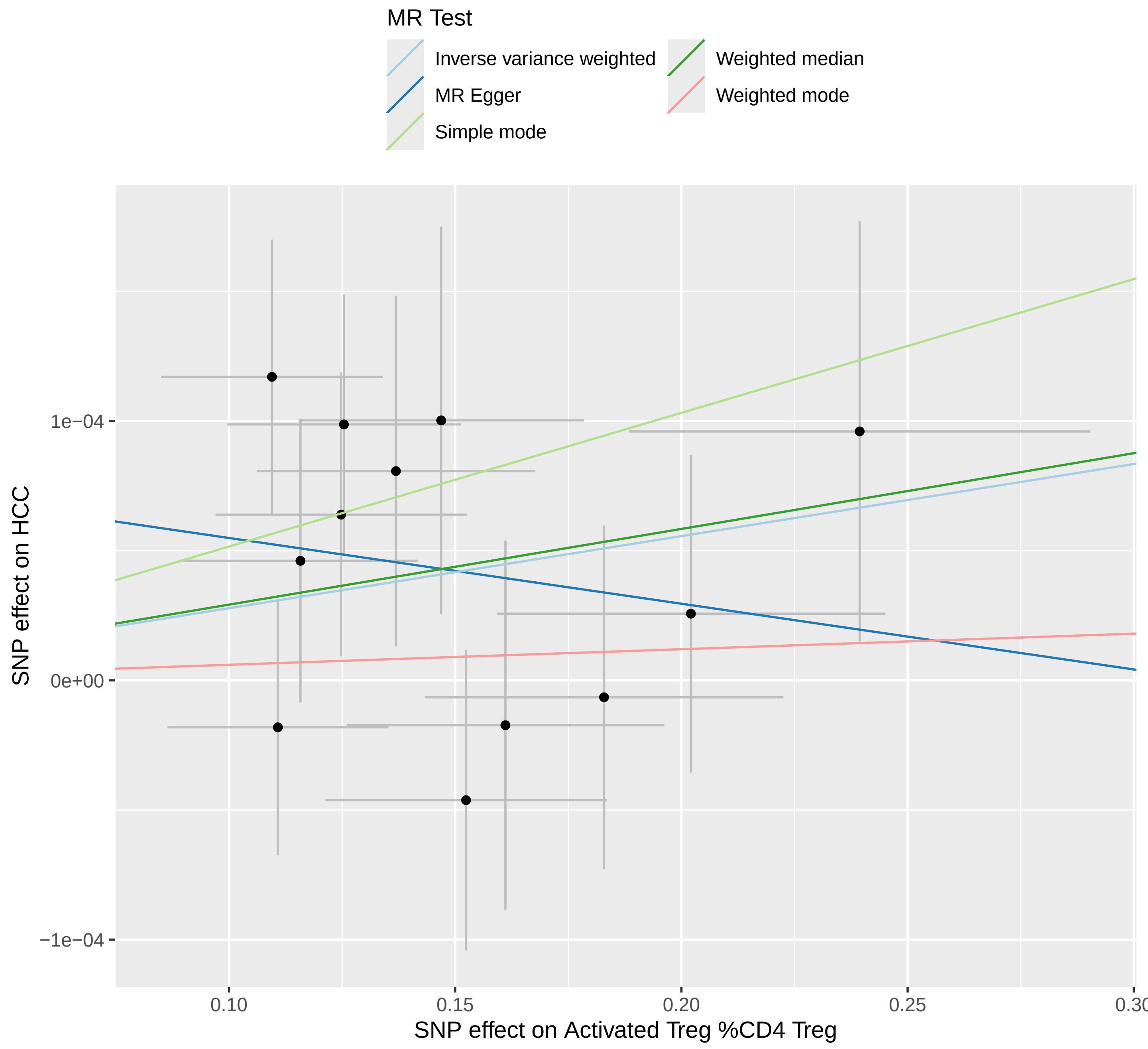
MR Test
Inverse variance weighted
MR Egger
Simple mode
Weighted median
Weighted mode
SNP effect on HCC
1e−04
0e+00
−1e−04
0.10
0.15
0.20
0.25
0.30
SNP effect on Activated Treg %CD4 Treg

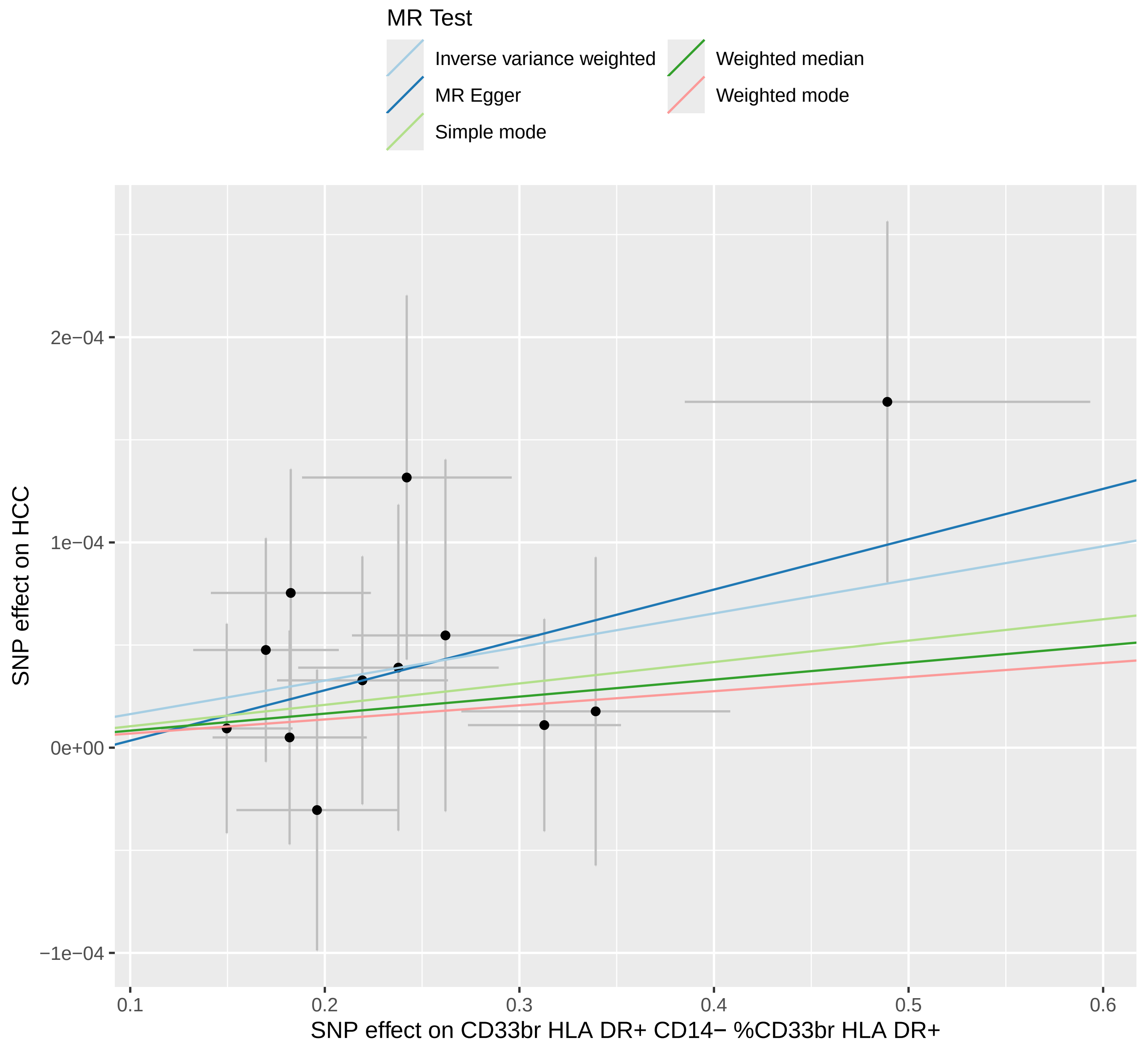
MR Test
Inverse variance weighted
MR Egger
Simple mode
Weighted median
Weighted mode
2e−04
1e−04
0e+00
−1e−04
SNP effect on HCC
0.1
0.2
0.3
0.4
0.5
0.6
SNP effect on CD33br HLA DR+ CD14− %CD33br HLA DR+

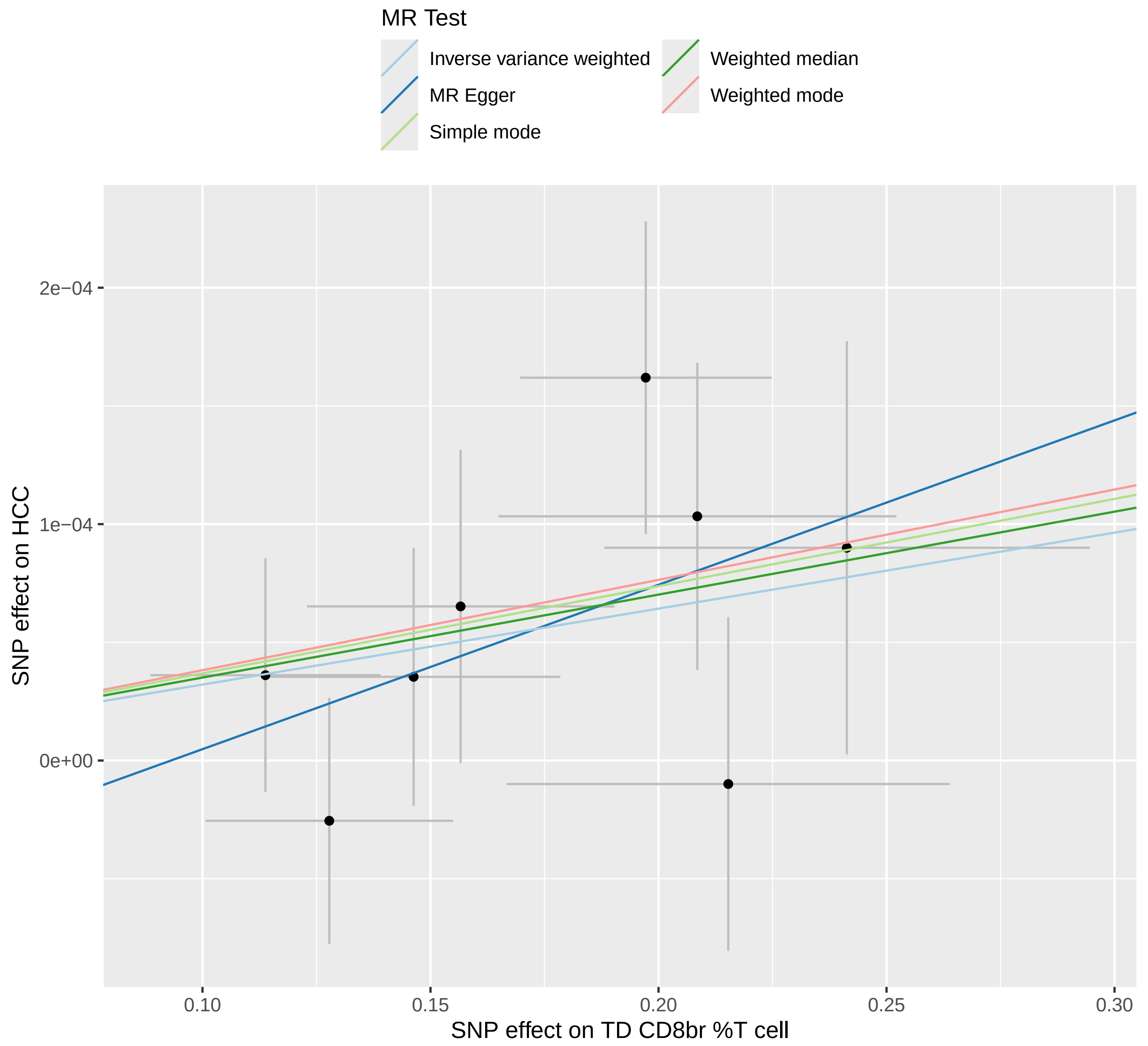

MR Test
Inverse variance weighted
MR Egger
Simple mode
Weighted median
Weighted mode
2e−04
1e−04
0e+00
SNP effect on HCC
0.10
0.15
0.20
0.25
0.30
SNP effect on TD CD8br %T cell

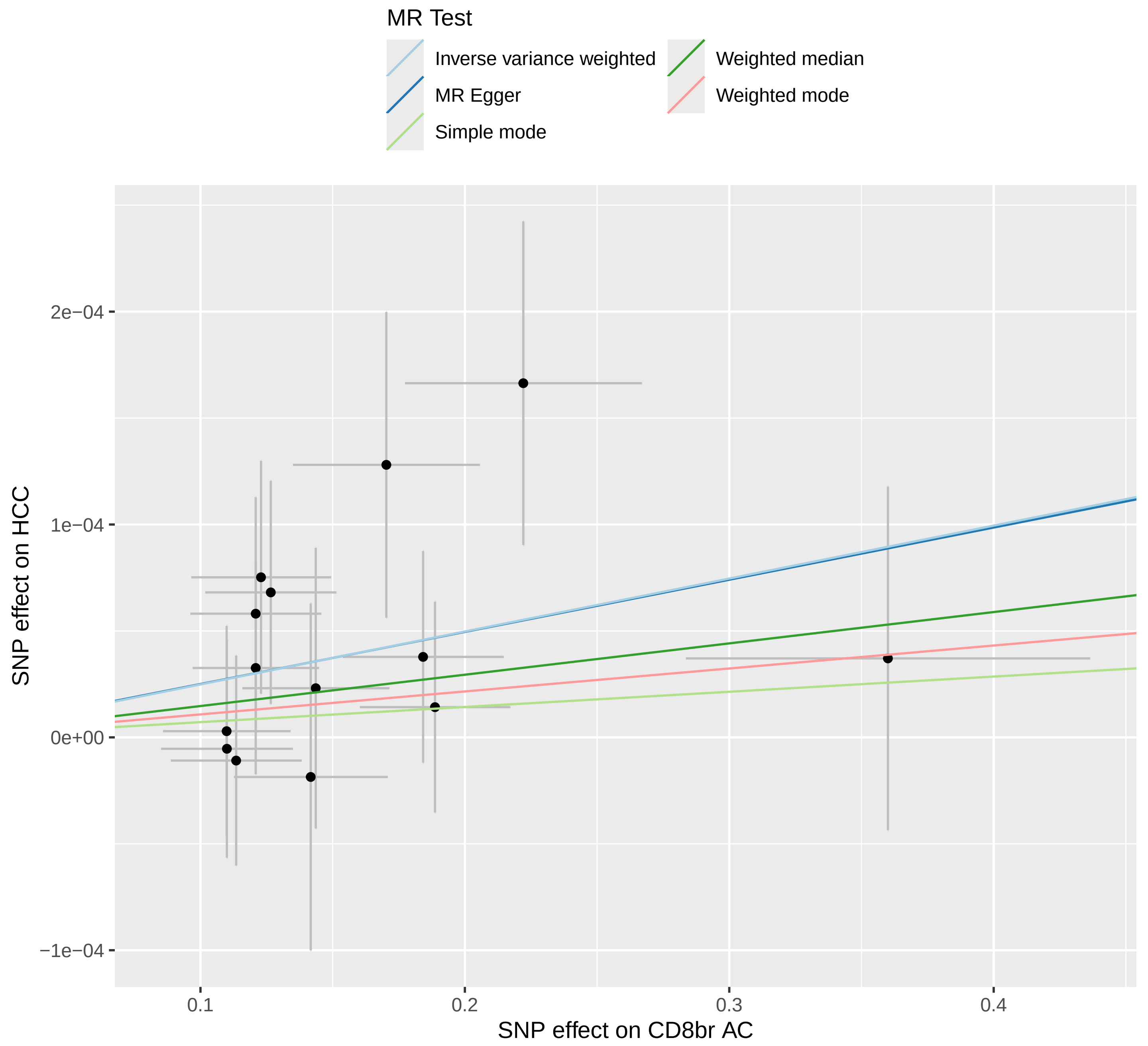
MR Test
Inverse variance weighted
MR Egger
Simple mode
Weighted median
Weighted mode
SNP effect on HCC
2e−04
1e−04
0e+00
−1e−04
0.1
0.2
0.3
0.4
SNP effect on CD8br AC

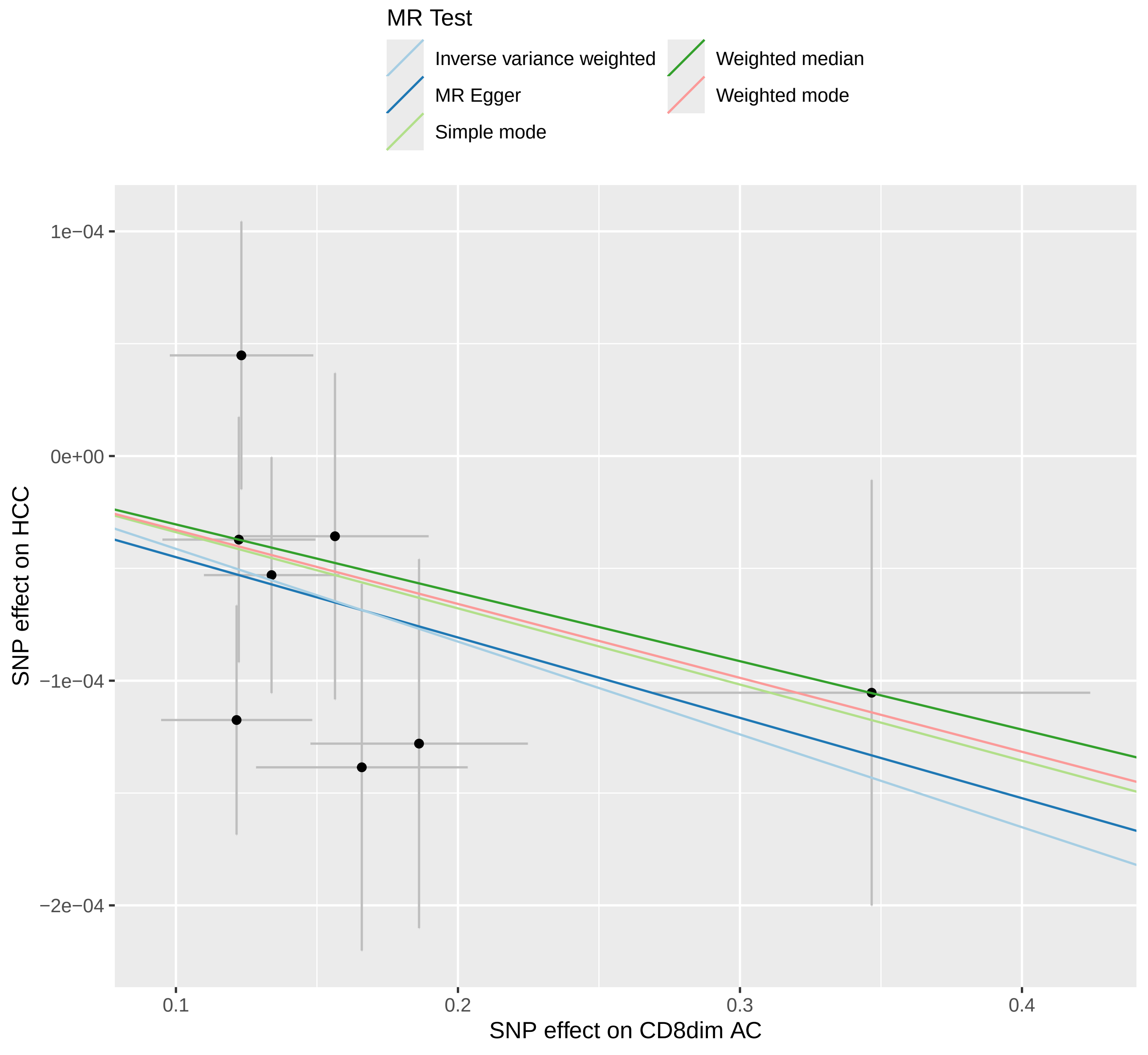
MR Test
Inverse variance weighted
MR Egger
Simple mode
Weighted median
Weighted mode
1e−04
0e+00
−1e−04
−2e−04
SNP effect on HCC
0.1
0.2
0.3
0.4
SNP effect on CD8dim AC

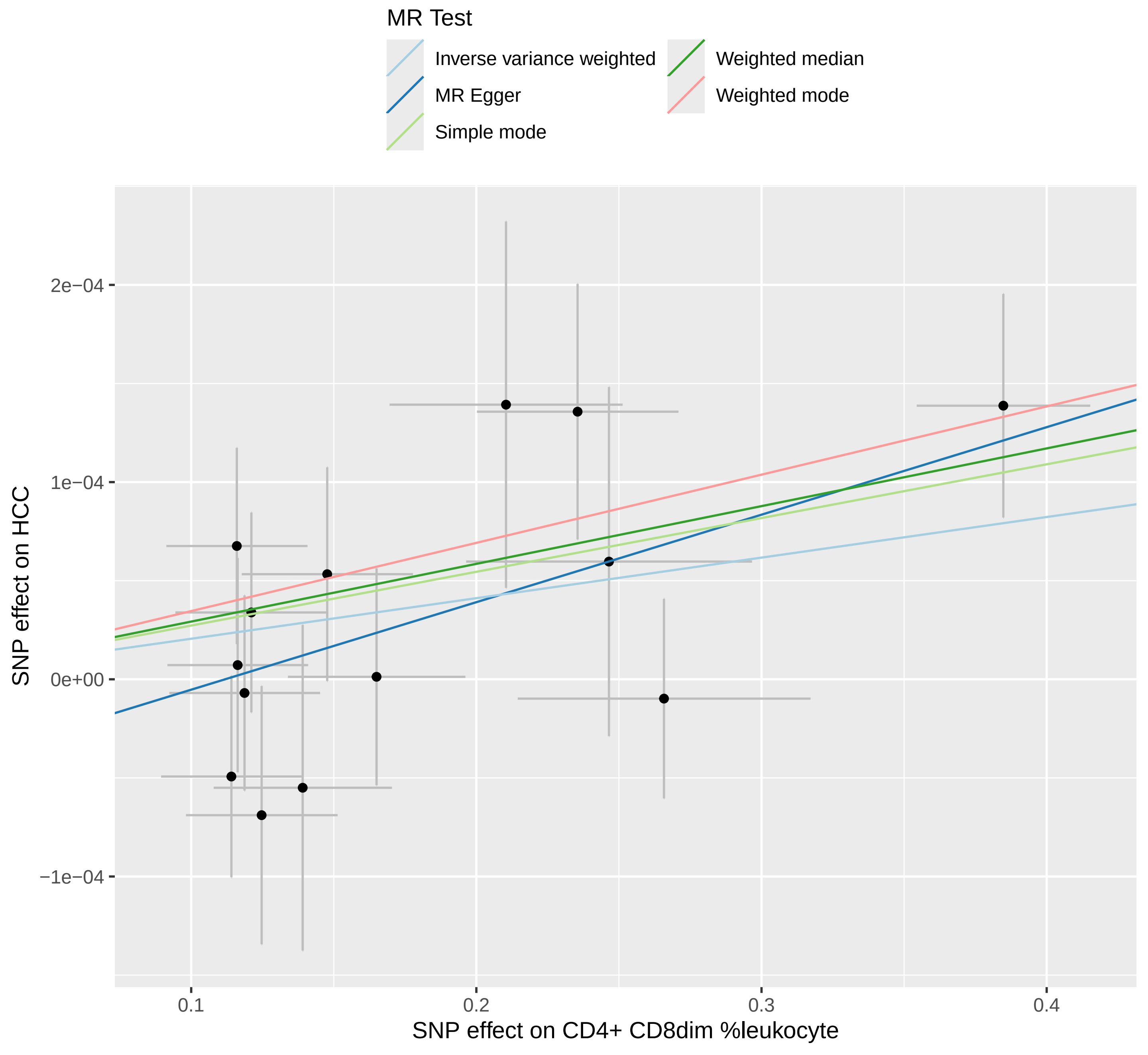
MR Test
Inverse variance weighted
MR Egger
Simple mode
Weighted median
Weighted mode
2e−04
1e−04
0e+00
−1e−04
0.1
0.2
0.3
0.4
SNP effect on HCC
SNP effect on CD4+ CD8dim %leukocyte

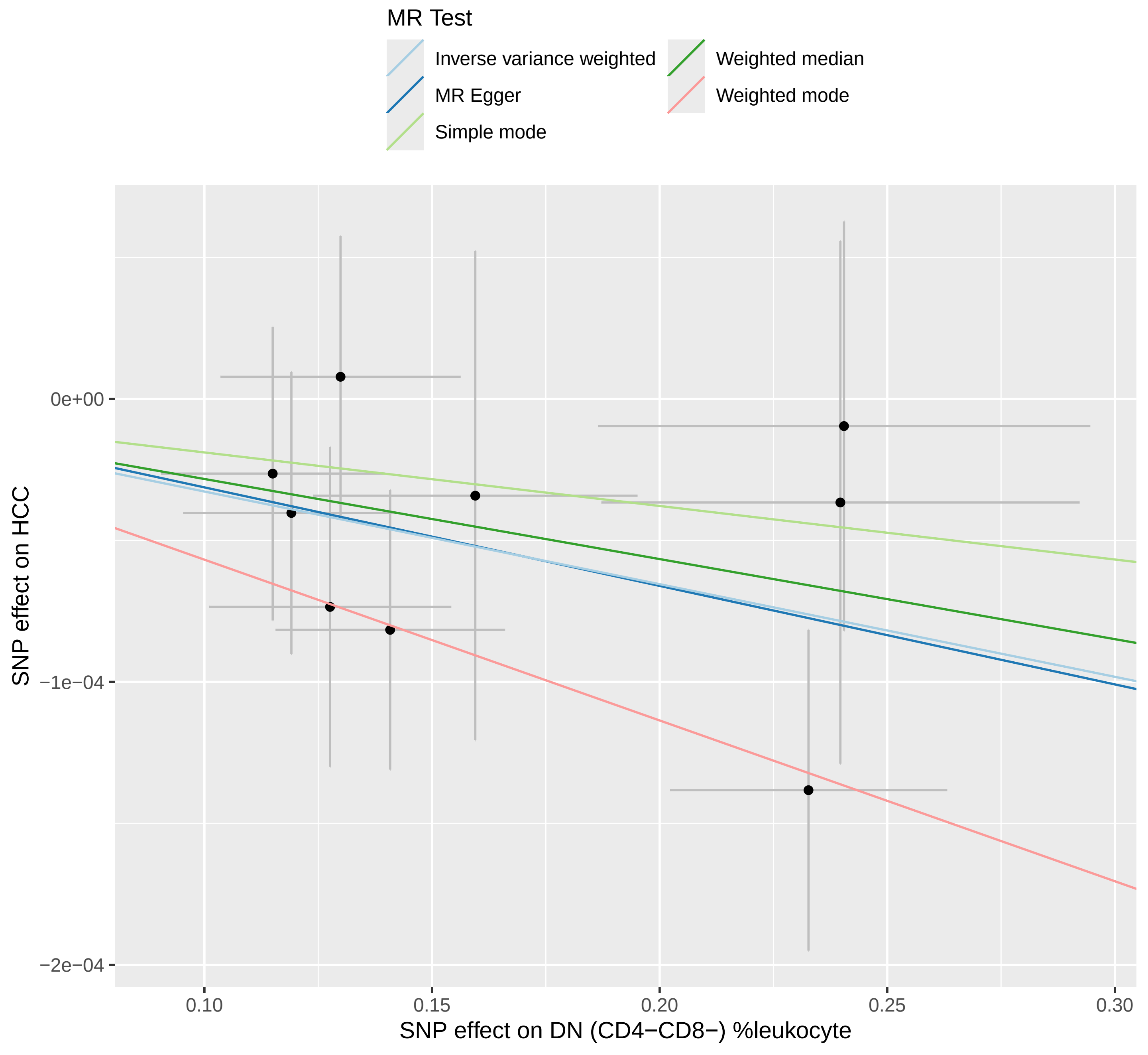
MR Test
Inverse variance weighted
MR Egger
Simple mode
Weighted median
Weighted mode
SNP effect on HCC
0e+00
−1e−04
−2e−04
0.10
0.15
0.20
0.25
0.30
SNP effect on DN (CD4−CD8−) %leukocyte

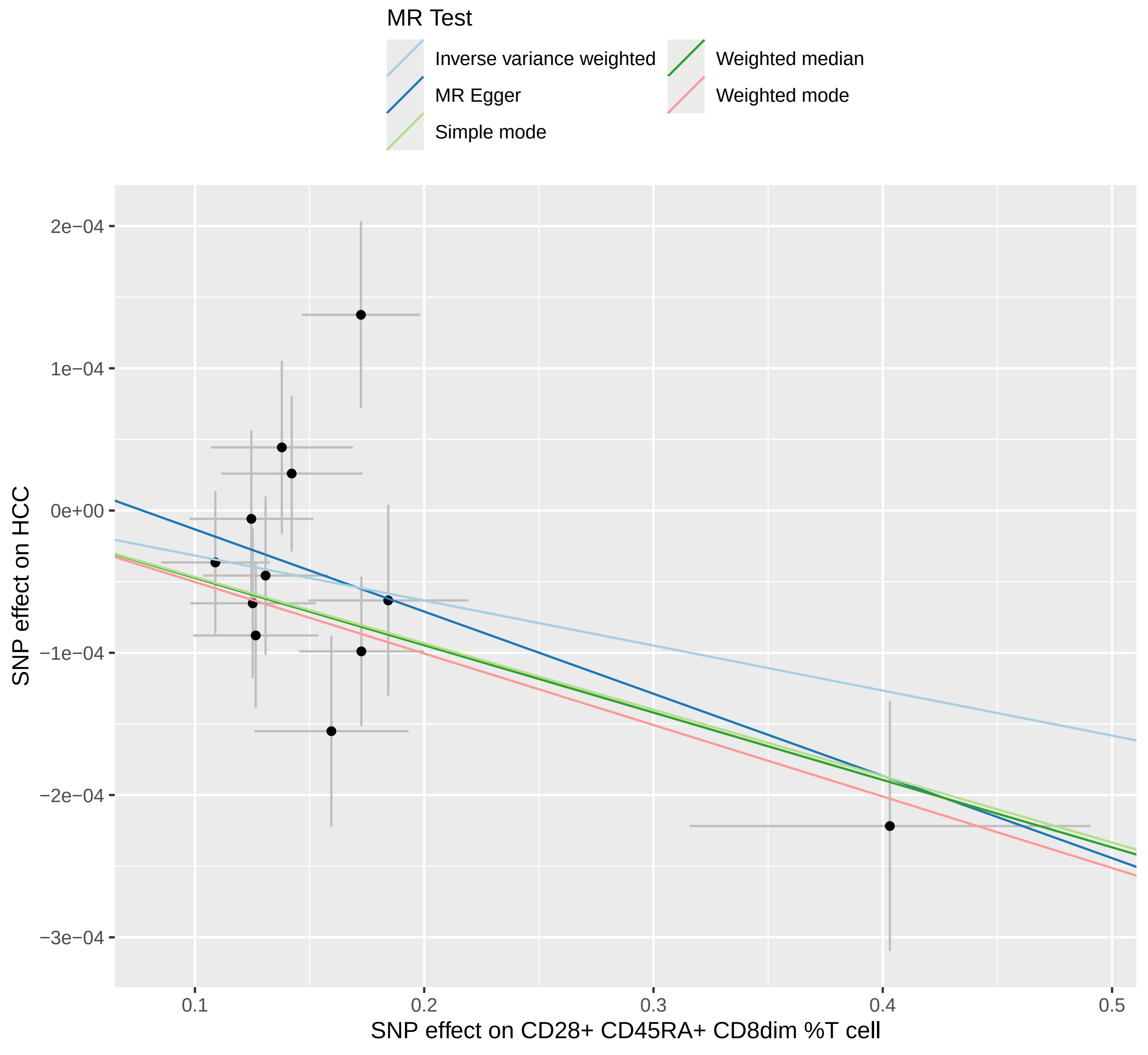

MR Test
Inverse variance weighted
MR Egger
Simple mode
Weighted median
Weighted mode
2e−04
1e−04
0e+00
−1e−04
−2e−04
−3e−04
SNP effect on HCC
0.1
0.2
0.3
0.4
0.5
SNP effect on CD28+ CD45RA+ CD8dim %T cell

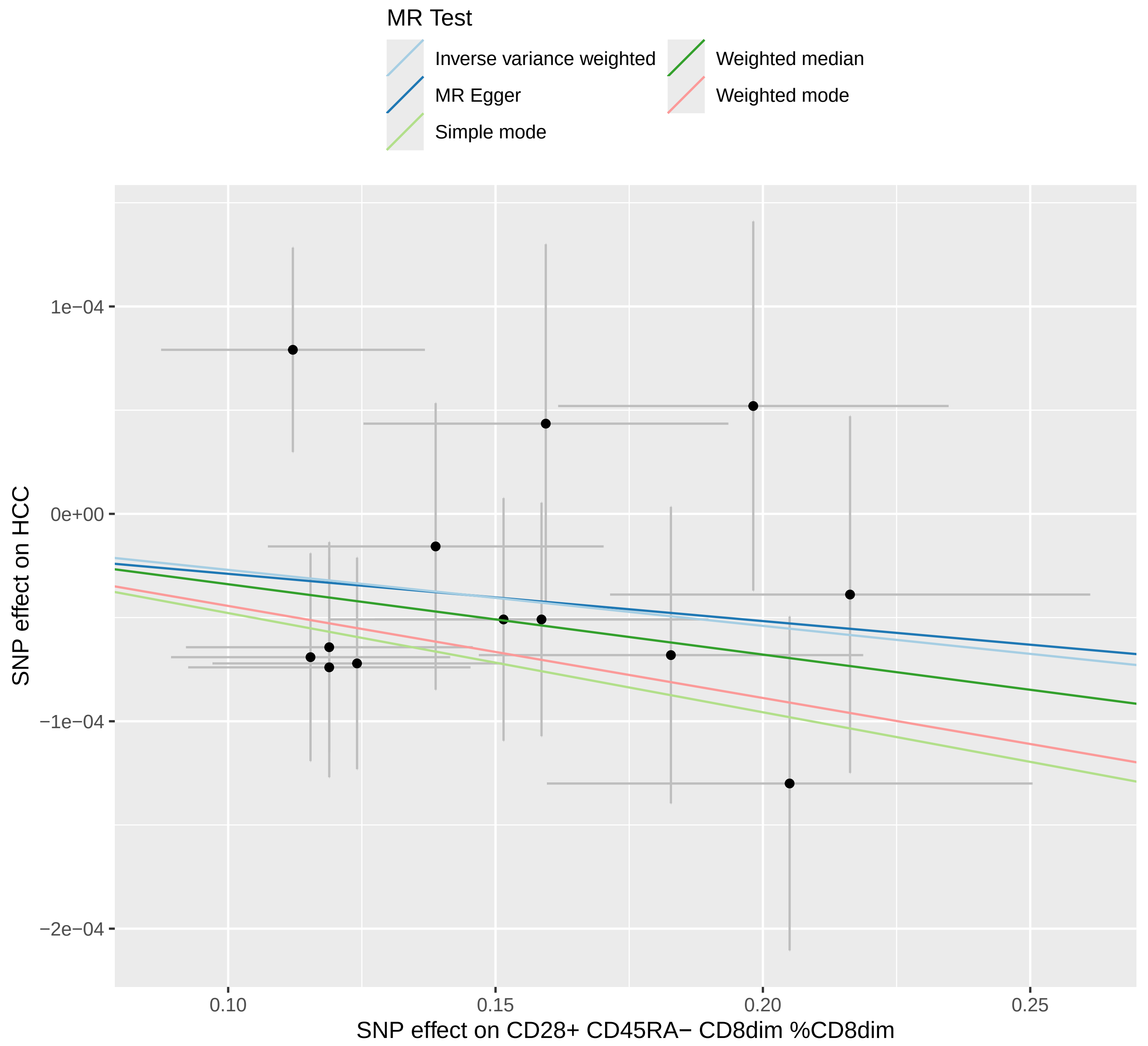

MR Test
Inverse variance weighted
MR Egger
Simple mode
Weighted median
Weighted mode
SNP effect on HCC
1e−04
0e+00
−1e−04
−2e−04
0.10
0.15
0.20
0.25
SNP effect on CD28+ CD45RA− CD8dim %CD8dim

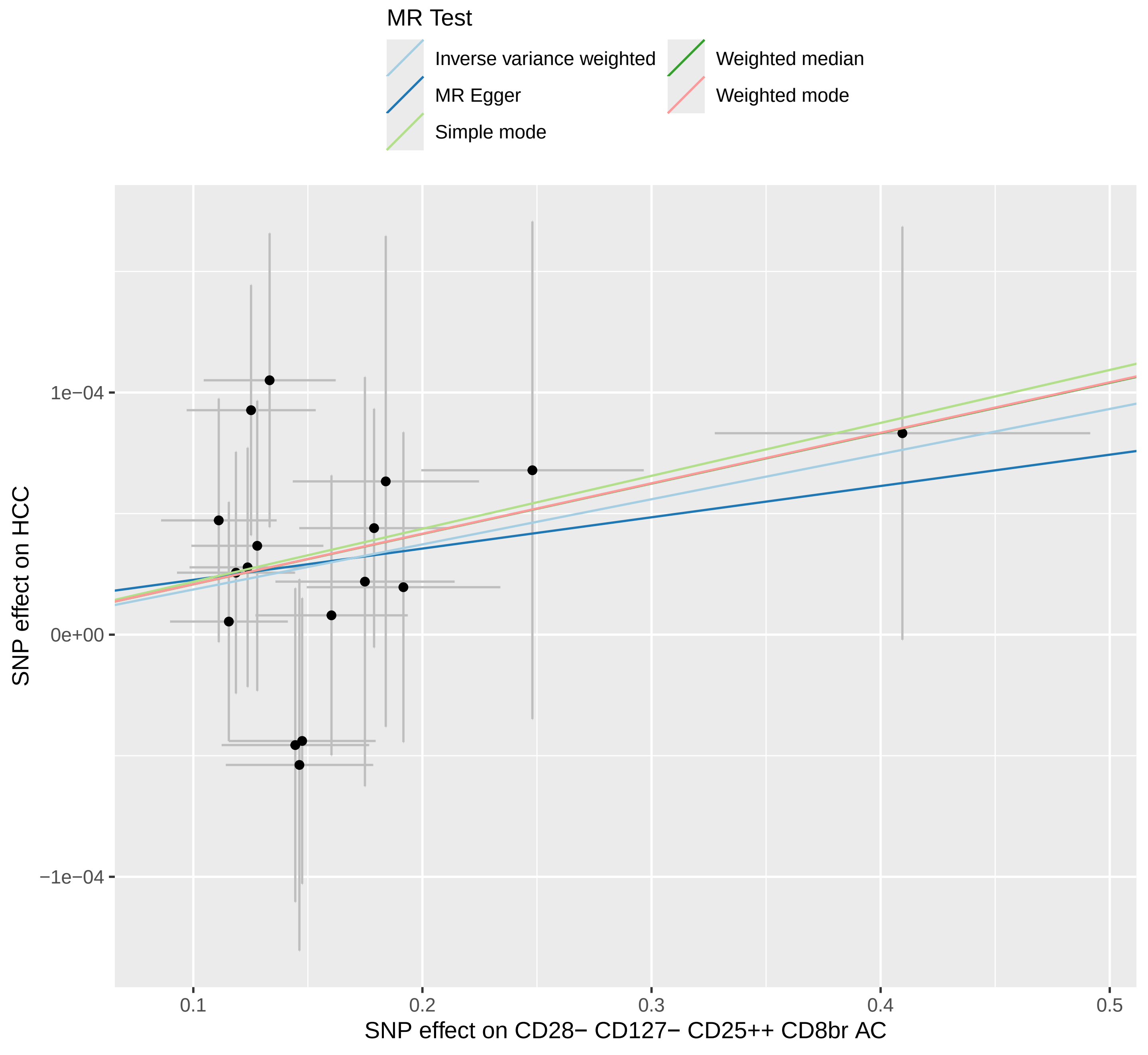
MR Test
Inverse variance weighted
MR Egger
Simple mode
Weighted median
Weighted mode
1e−04
0e+00
−1e−04
0.1
0.2
0.3
0.4
0.5
SNP effect on HCC
SNP effect on CD28− CD127− CD25++ CD8br AC

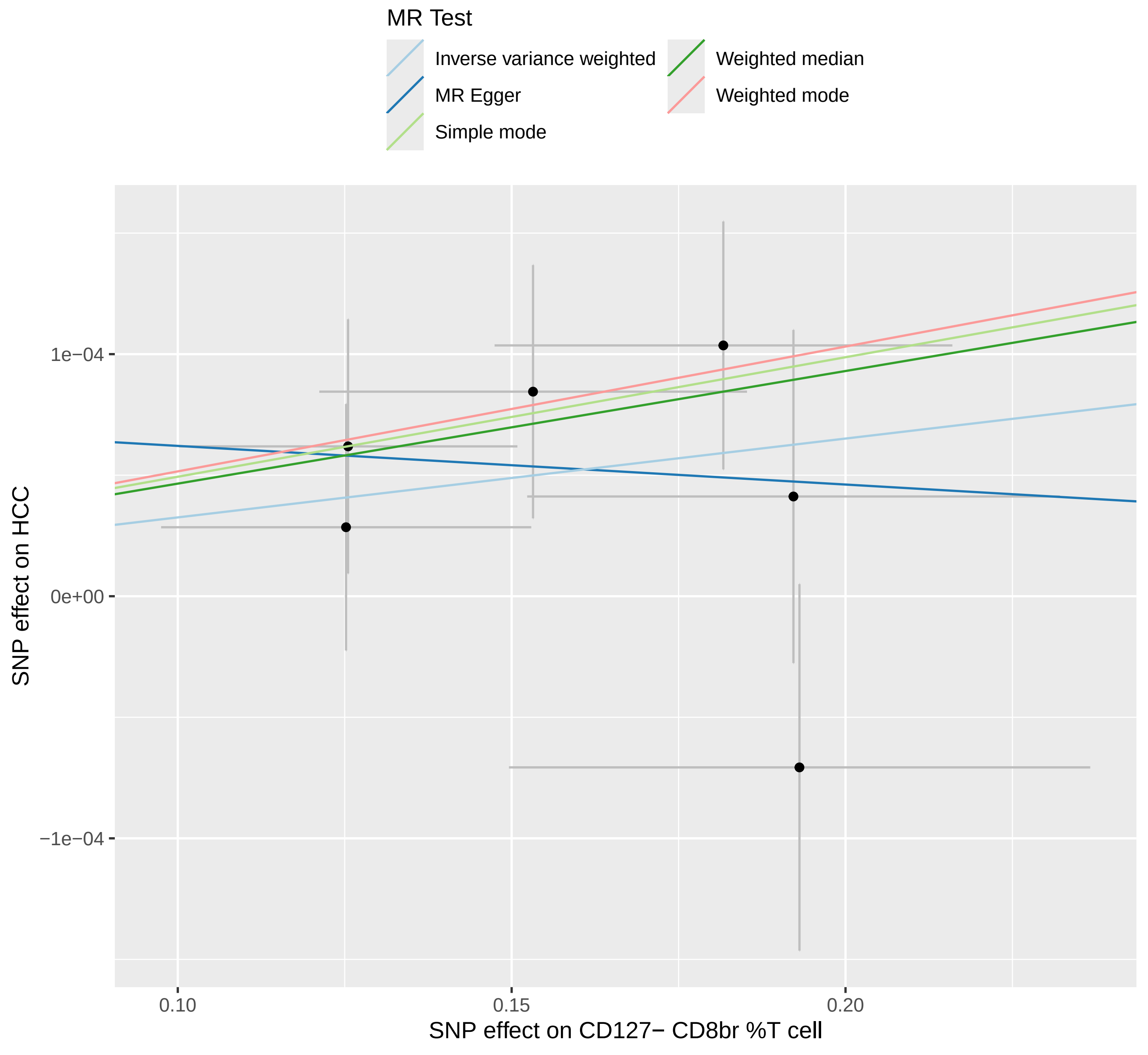
MR Test
Inverse variance weighted
MR Egger
Simple mode
Weighted median
Weighted mode
1e−04
0e+00
−1e−04
SNP effect on HCC
0.10
0.15
0.20
SNP effect on CD127− CD8br %T cell

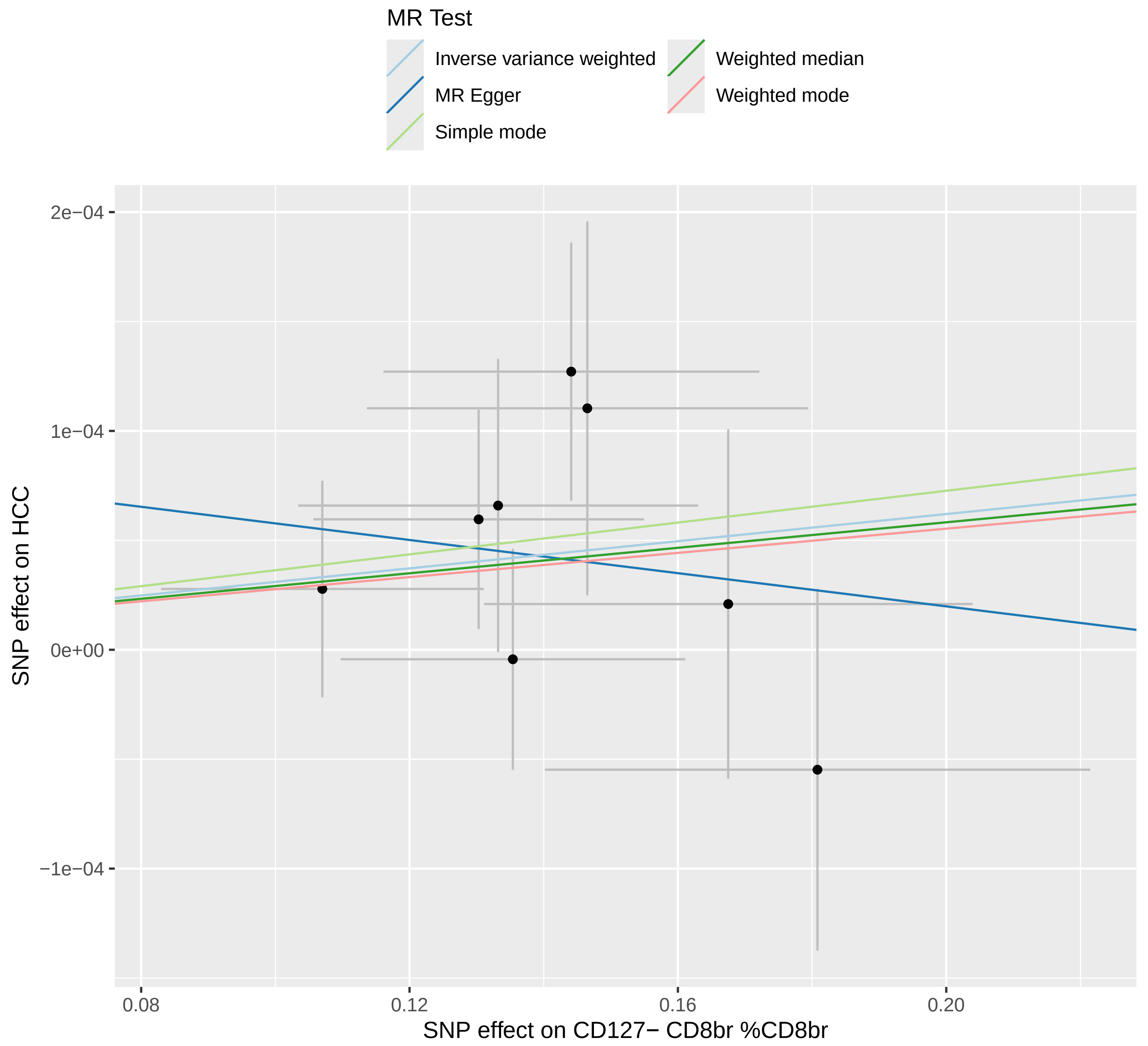

MR Test
Inverse variance weighted
MR Egger
Simple mode
Weighted median
Weighted mode
2e−04
1e−04
0e+00
−1e−04
SNP effect on HCC
0.08
0.12
0.16
0.20
SNP effect on CD127− CD8br %CD8br

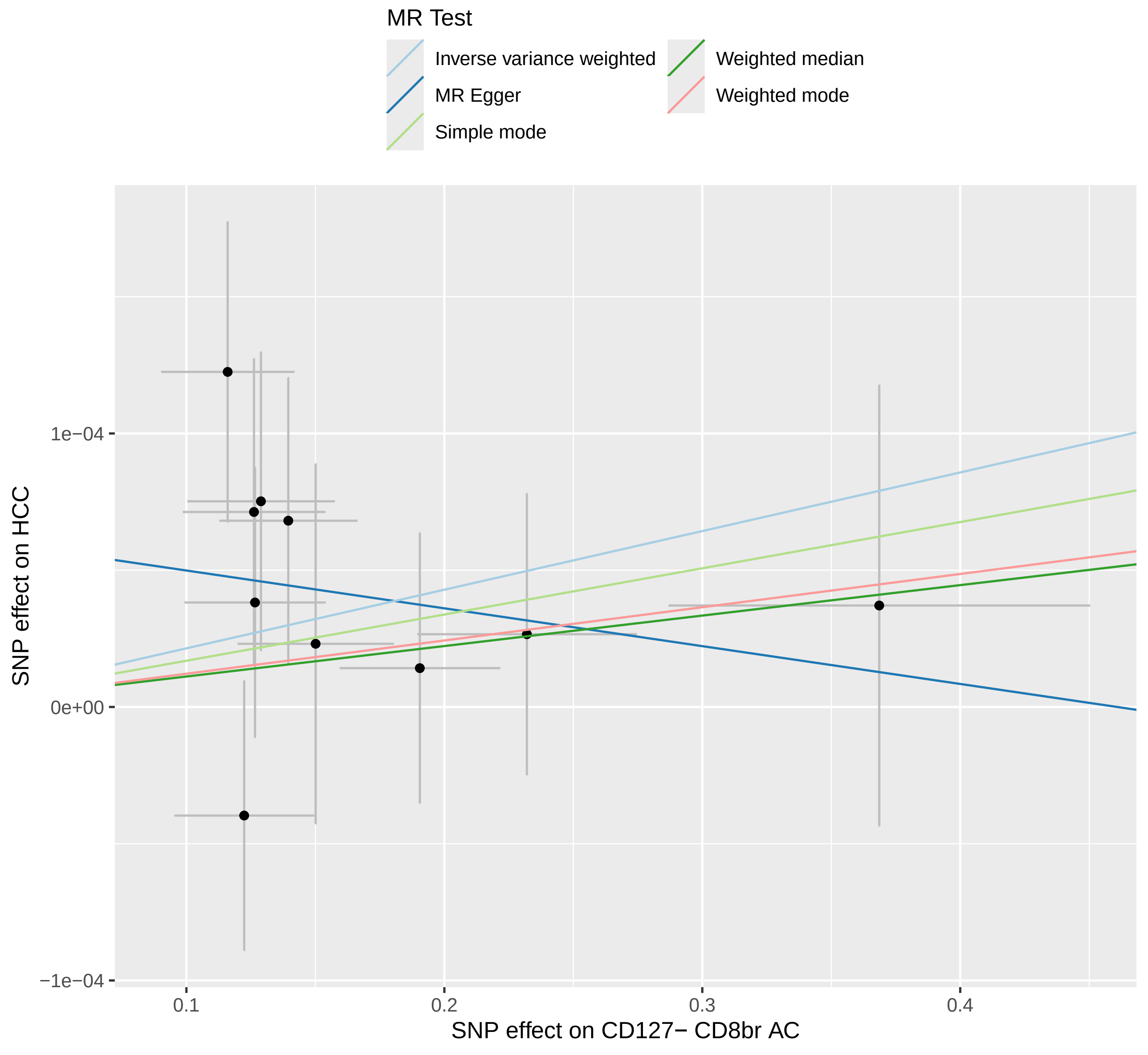

MR Test
Inverse variance weighted
MR Egger
Simple mode
Weighted median
Weighted mode
1e−04
0e+00
−1e−04
SNP effect on HCC
0.1
0.2
0.3
0.4
SNP effect on CD127− CD8br AC

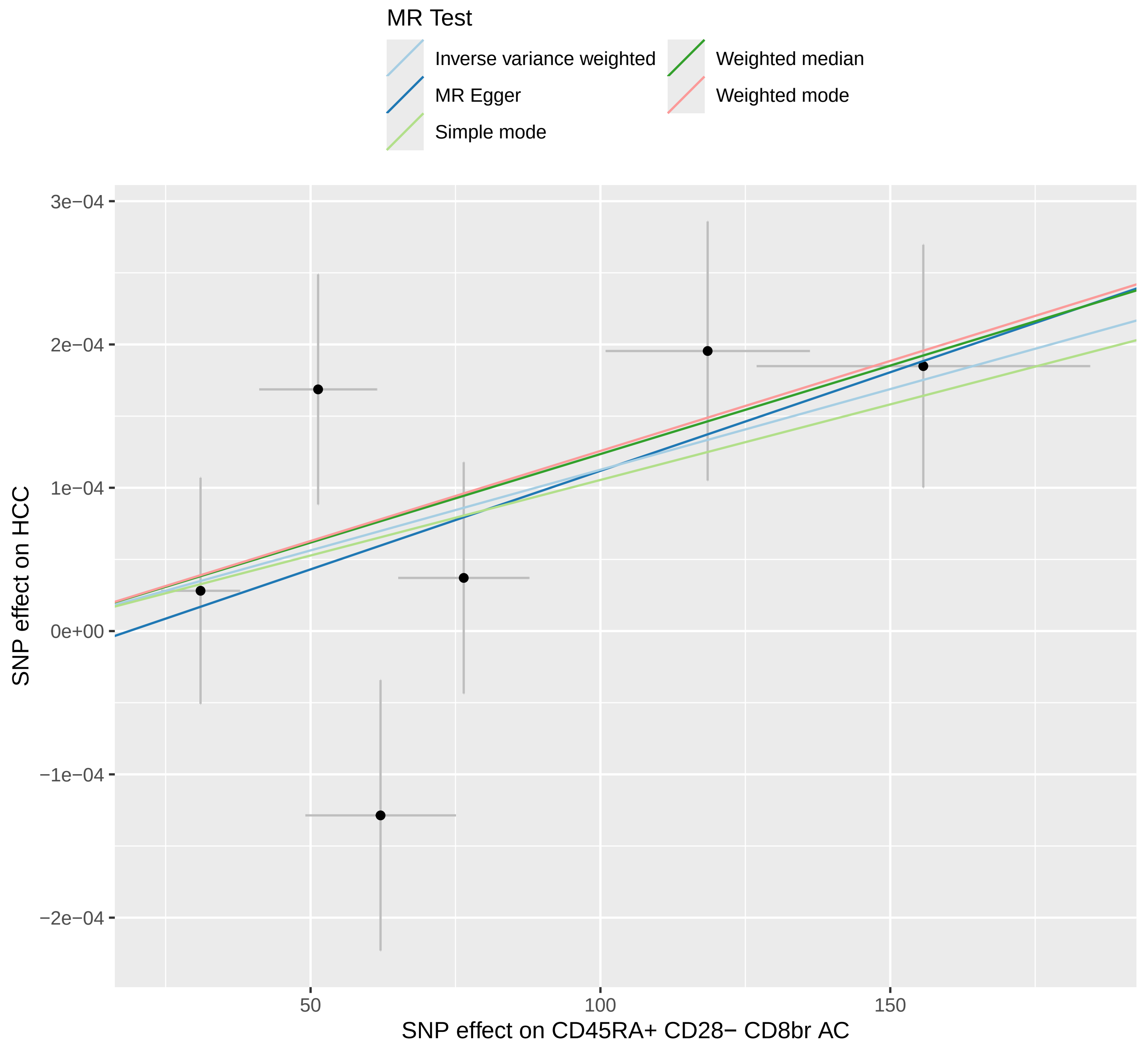
MR Test
Inverse variance weighted
MR Egger
Simple mode
Weighted median
Weighted mode
3e−04
2e−04
1e−04
0e+00
−1e−04
−2e−04
SNP effect on HCC
50
100
150
SNP effect on CD45RA+ CD28− CD8br AC

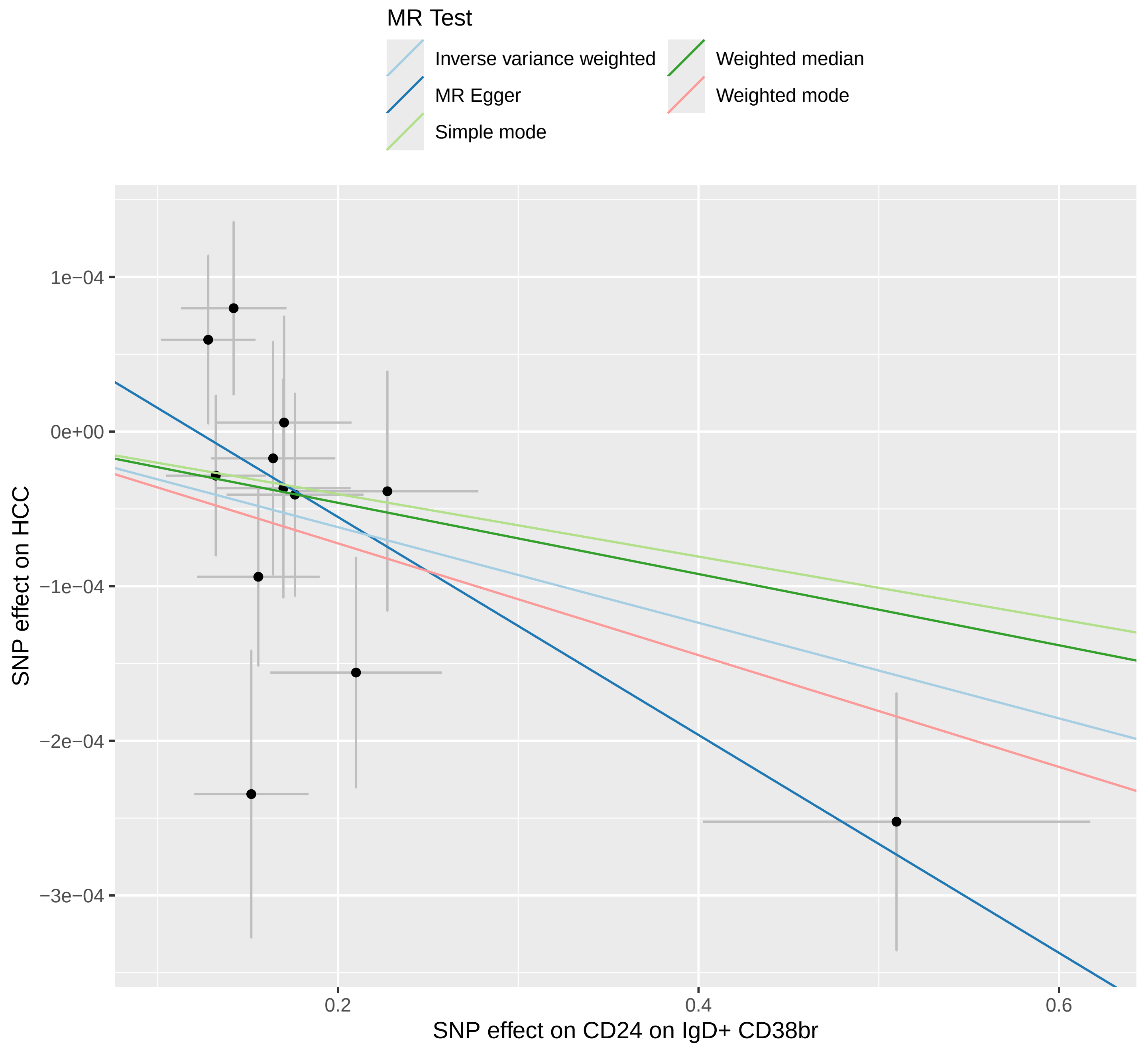
MR Test
Inverse variance weighted
MR Egger
Simple mode
Weighted median
Weighted mode
1e−04
0e+00
−1e−04
−2e−04
−3e−04
0.2
0.4
0.6
SNP effect on HCC
SNP effect on CD24 on IgD+ CD38br

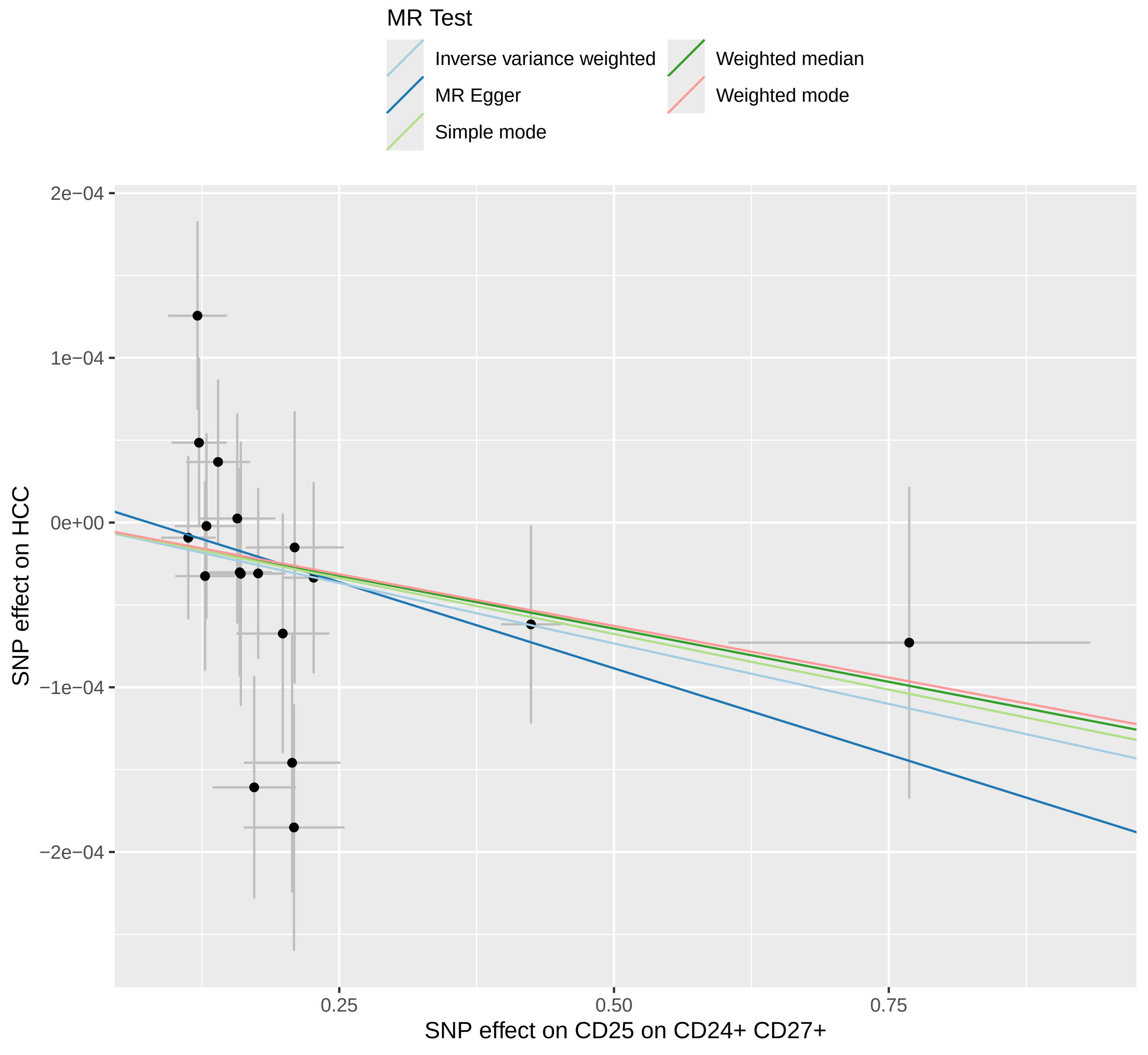
MR Test
Inverse variance weighted
MR Egger
Simple mode
Weighted median
Weighted mode
2e−04
1e−04
0e+00
−1e−04
−2e−04
SNP effect on HCC
0.25
0.50
0.75
SNP effect on CD25 on CD24+ CD27+

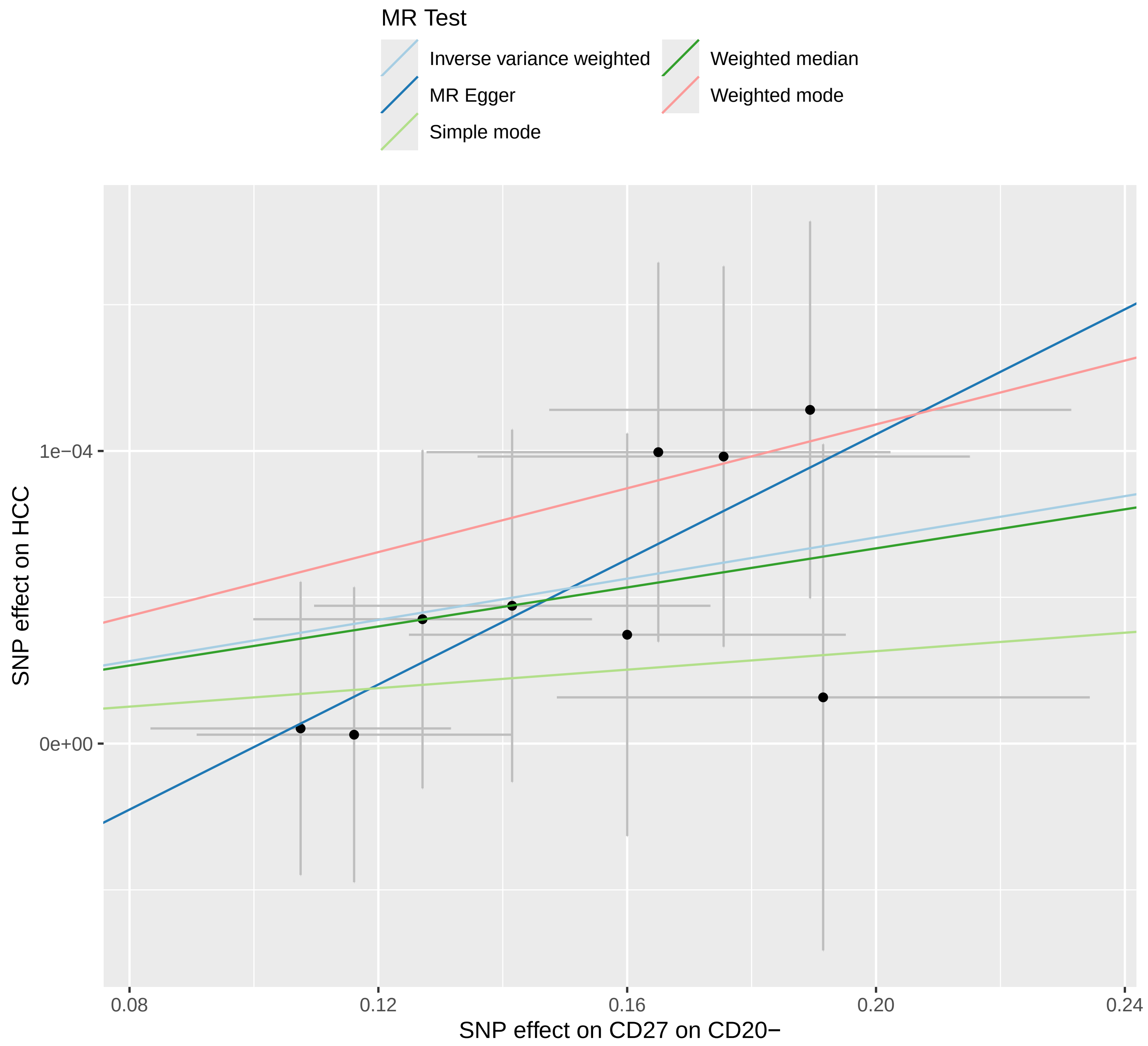

MR Test
Inverse variance weighted
MR Egger
Simple mode
Weighted median
Weighted mode
1e−04
0e+00
0.08
0.12
0.16
0.20
0.24
SNP effect on HCC
SNP effect on CD27 on CD20−

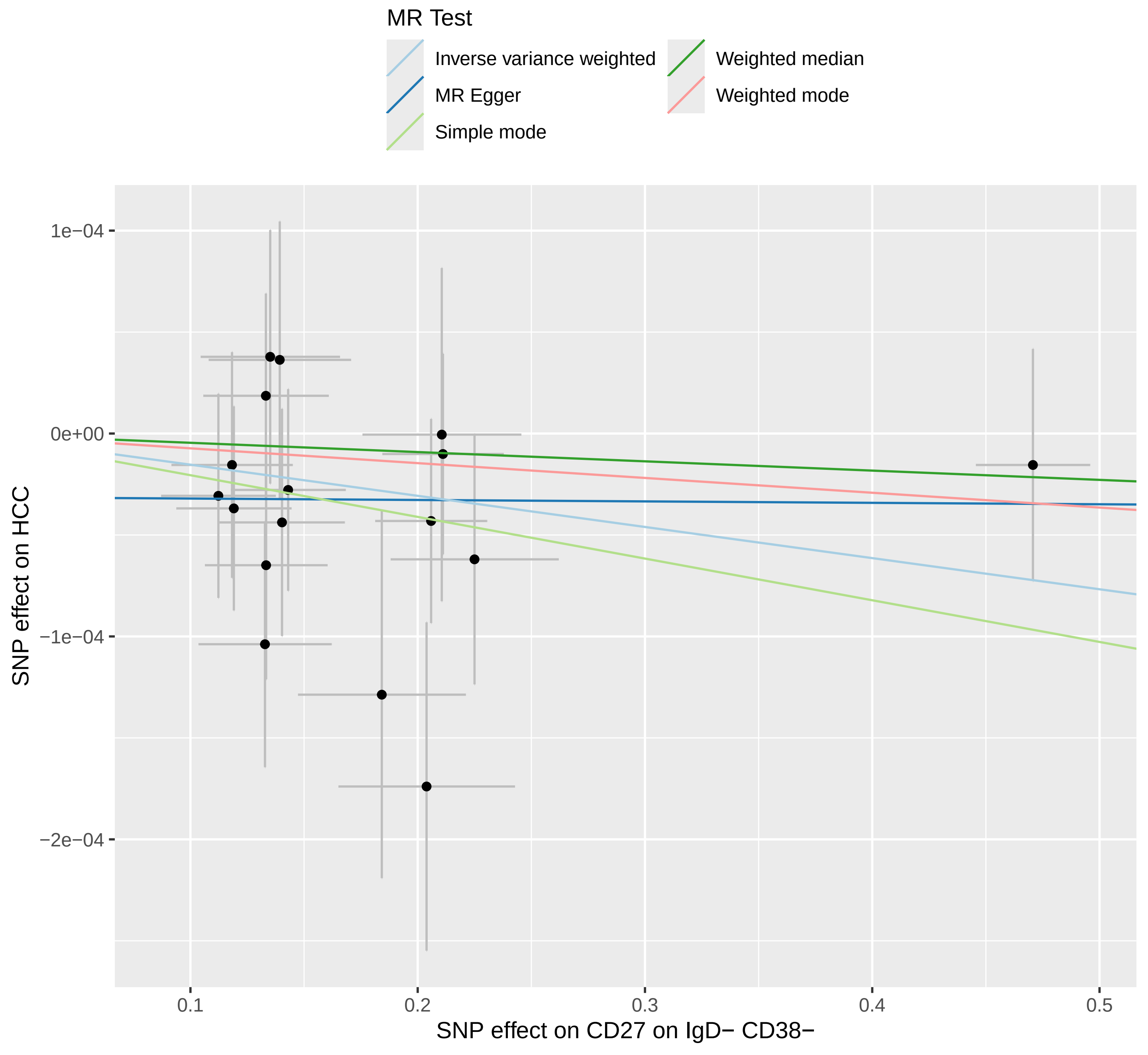

MR Test
Inverse variance weighted
MR Egger
Simple mode
Weighted median
Weighted mode
1e−04
0e+00
−1e−04
−2e−04
SNP effect on HCC
0.1
0.2
0.3
0.4
0.5
SNP effect on CD27 on IgD− CD38−

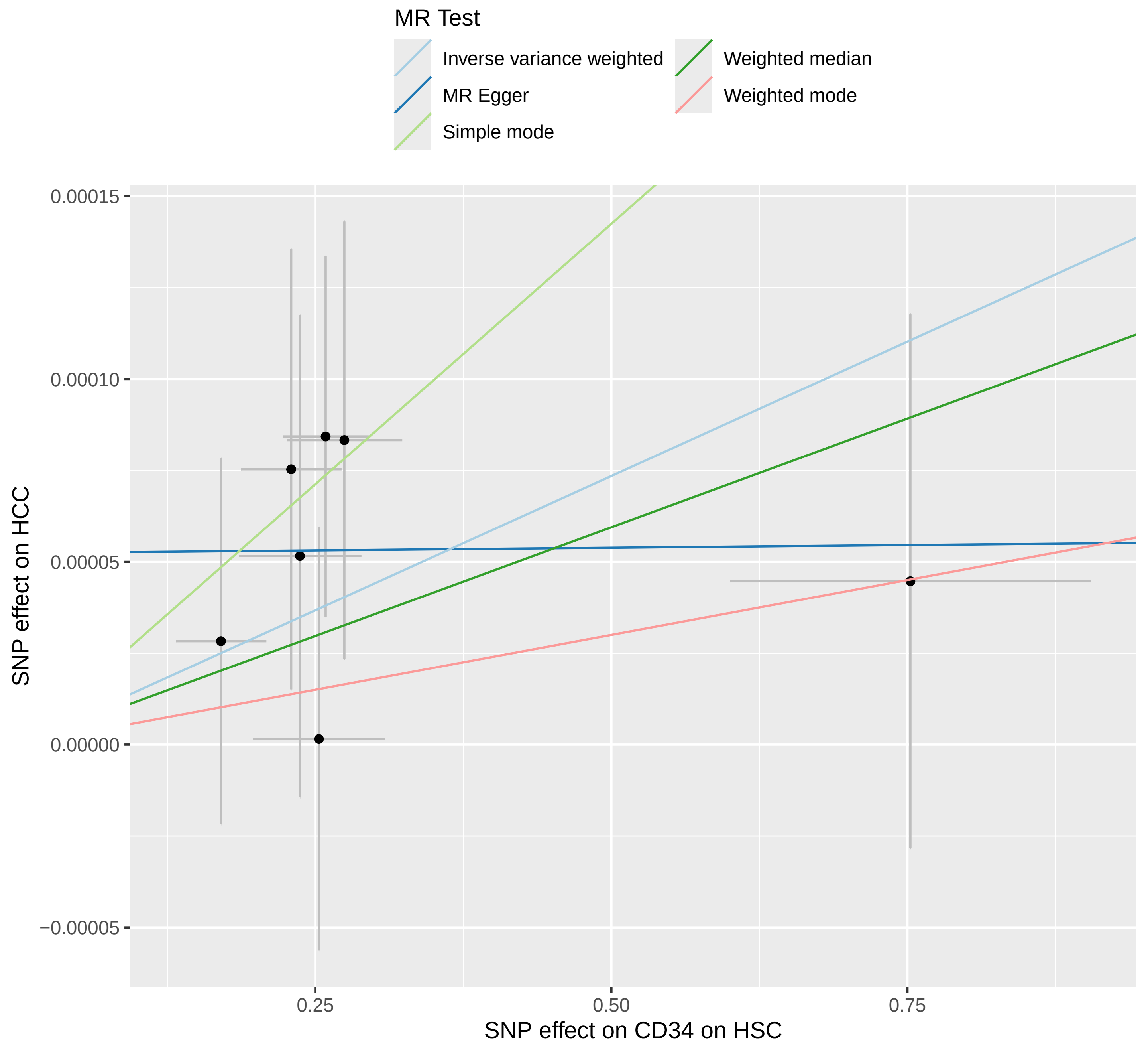

MR Test
Inverse variance weighted
MR Egger
Simple mode
Weighted median
Weighted mode
0.00015
0.00010
0.00005
0.00000
−0.00005
SNP effect on HCC
0.25
0.50
0.75
SNP effect on CD34 on HSC

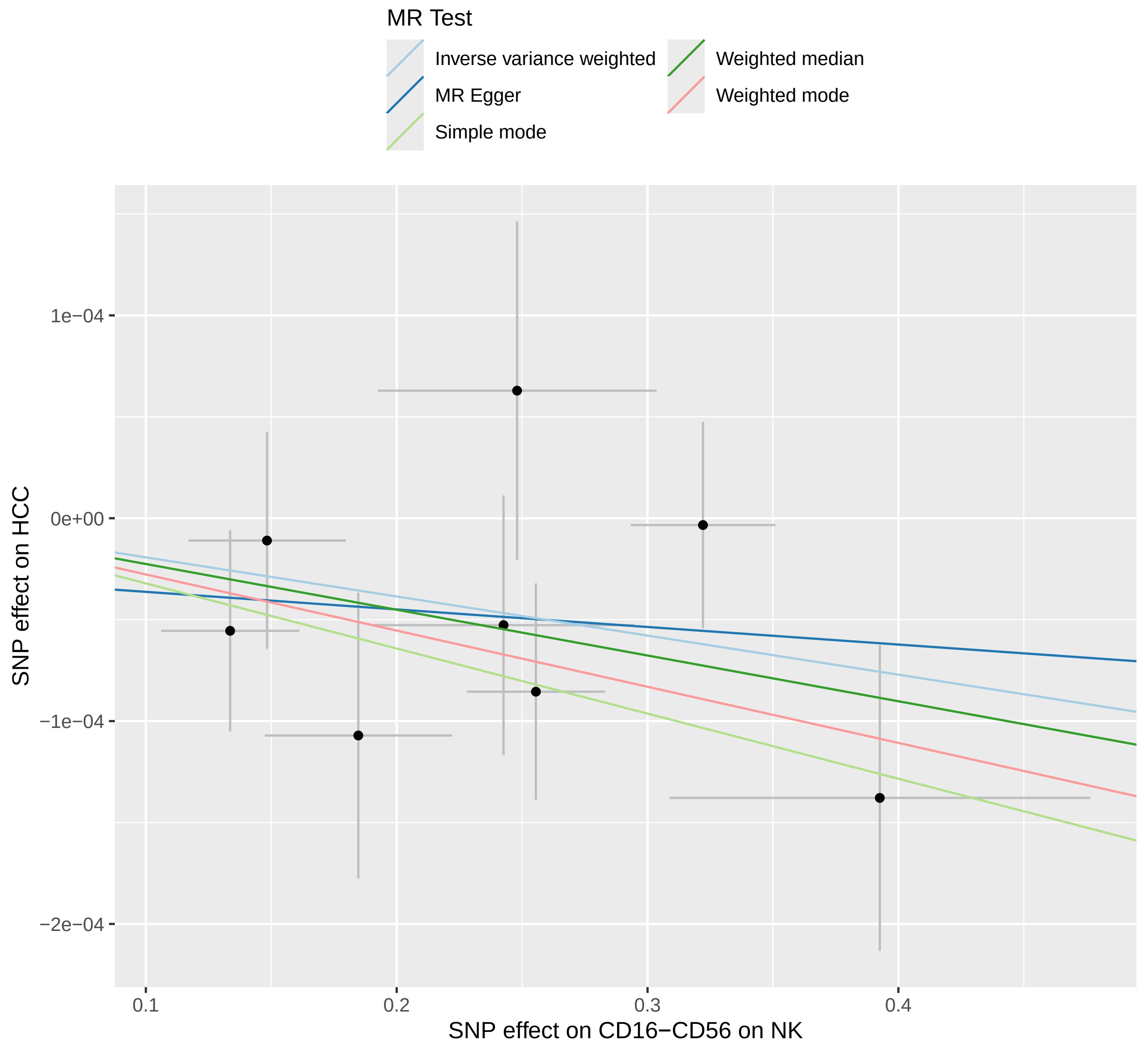
MR Test
Inverse variance weighted
MR Egger
Simple mode
Weighted median
Weighted mode
1e−04
0e+00
−1e−04
−2e−04
SNP effect on HCC
0.1
0.2
0.3
0.4
SNP effect on CD16−CD56 on NK

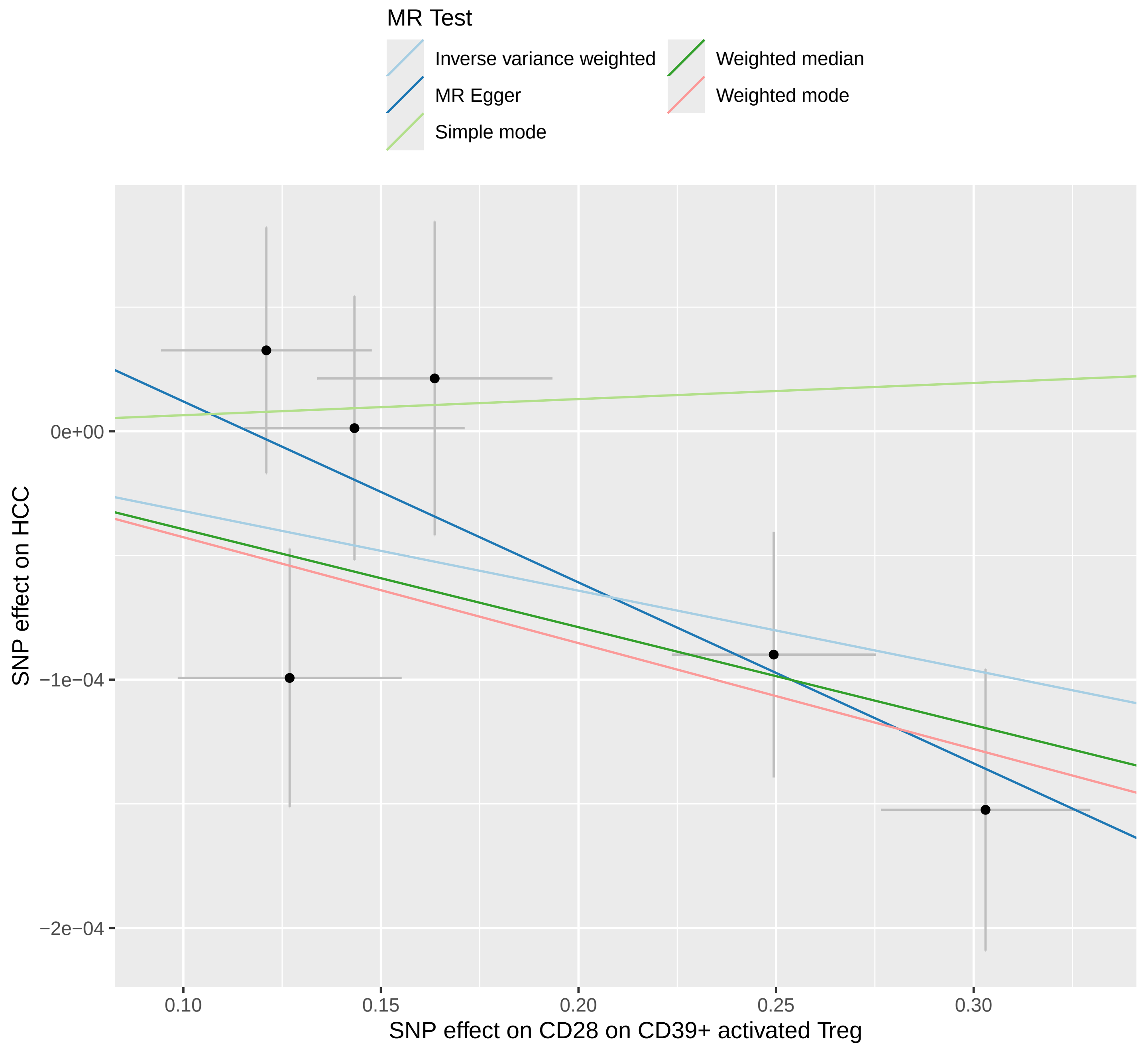
MR Test
Inverse variance weighted
MR Egger
Simple mode
Weighted median
Weighted mode
SNP effect on HCC
0e+00
−1e−04
−2e−04
0.10
0.15
0.20
0.25
0.30
SNP effect on CD28 on CD39+ activated Treg

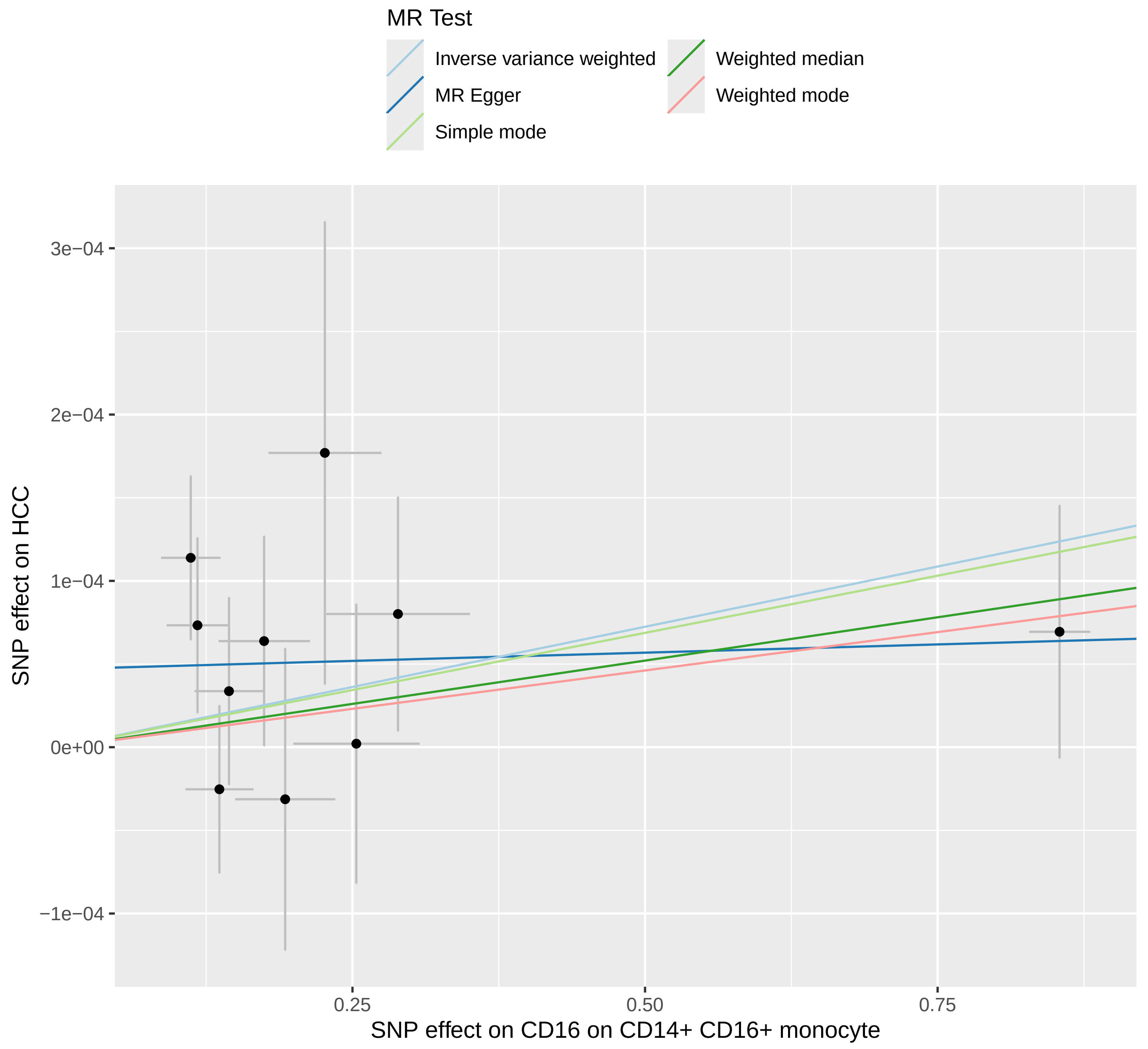
MR Test
Inverse variance weighted
MR Egger
Simple mode
Weighted median
Weighted mode
3e−04
2e−04
1e−04
0e+00
−1e−04
SNP effect on HCC
0.25
0.50
0.75
SNP effect on CD16 on CD14+ CD16+ monocyte

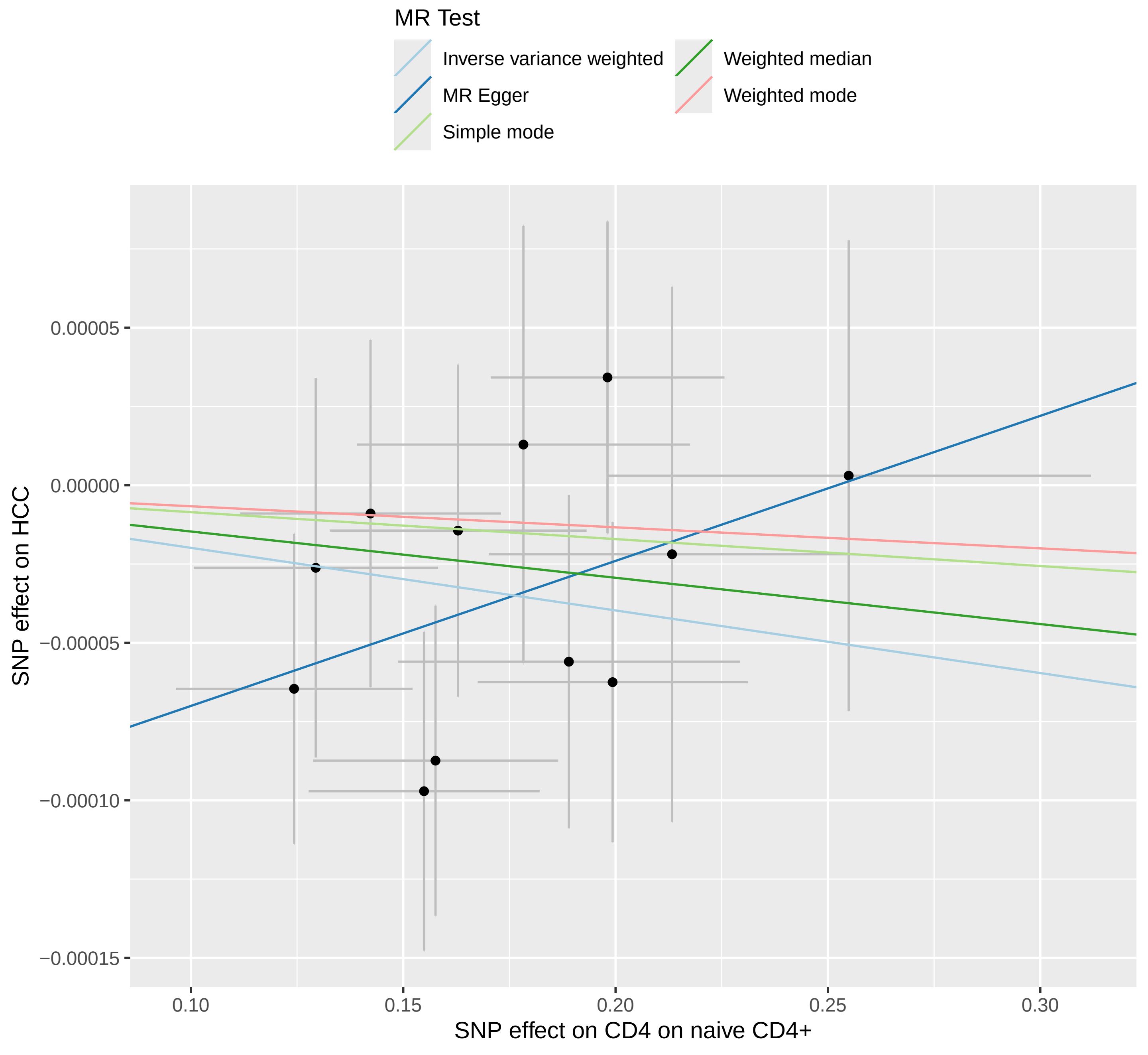

MR Test
Inverse variance weighted
MR Egger
Simple mode
Weighted median
Weighted mode
0.00005
0.00000
−0.00005
−0.00010
−0.00015
SNP effect on HCC
0.10
0.15
0.20
0.25
0.30
SNP effect on CD4 on naive CD4+

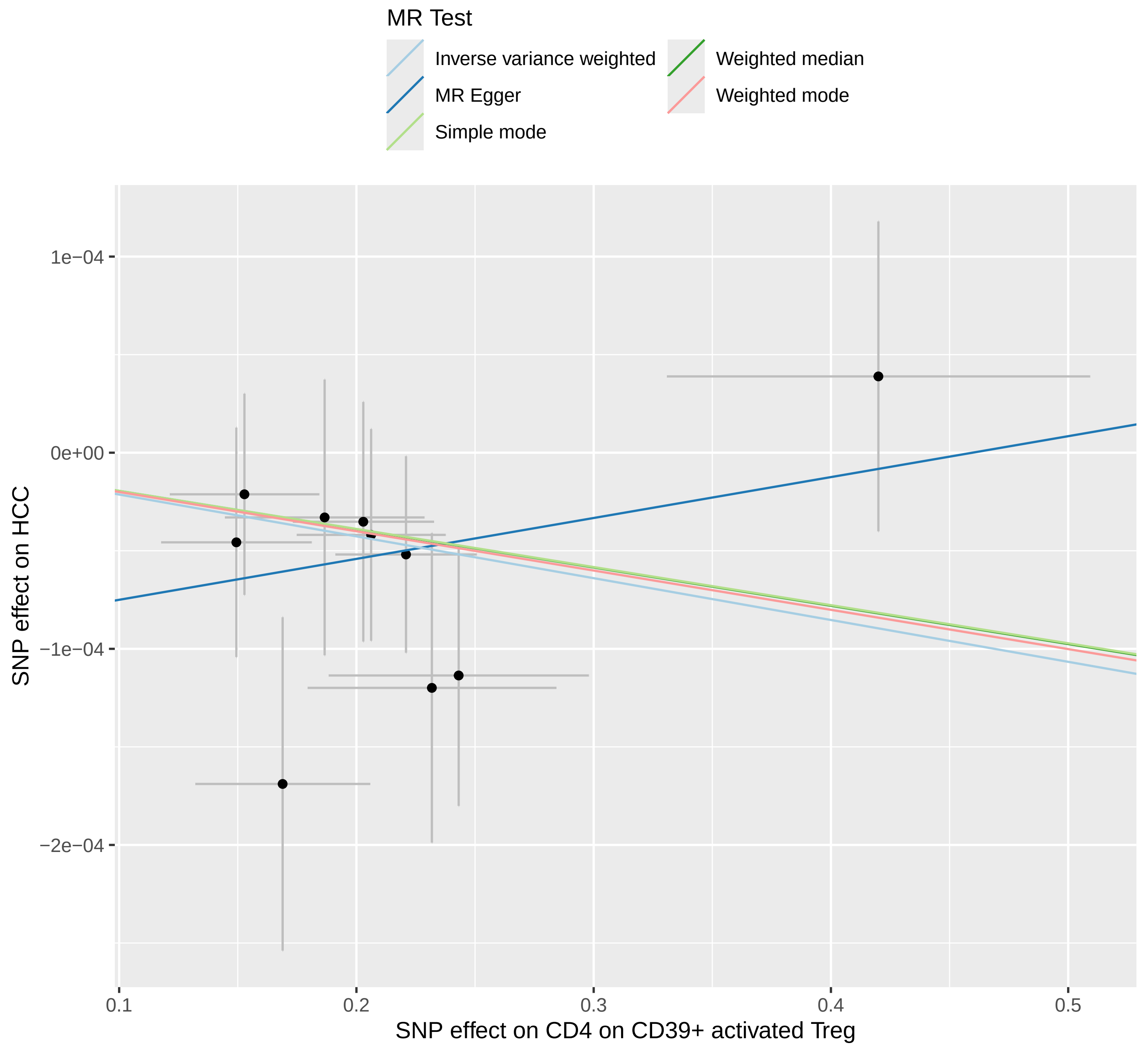

MR Test
Inverse variance weighted
MR Egger
Simple mode
Weighted median
Weighted mode
1e−04
0e+00
−1e−04
−2e−04
SNP effect on HCC
0.1
0.2
0.3
0.4
0.5
SNP effect on CD4 on CD39+ activated Treg

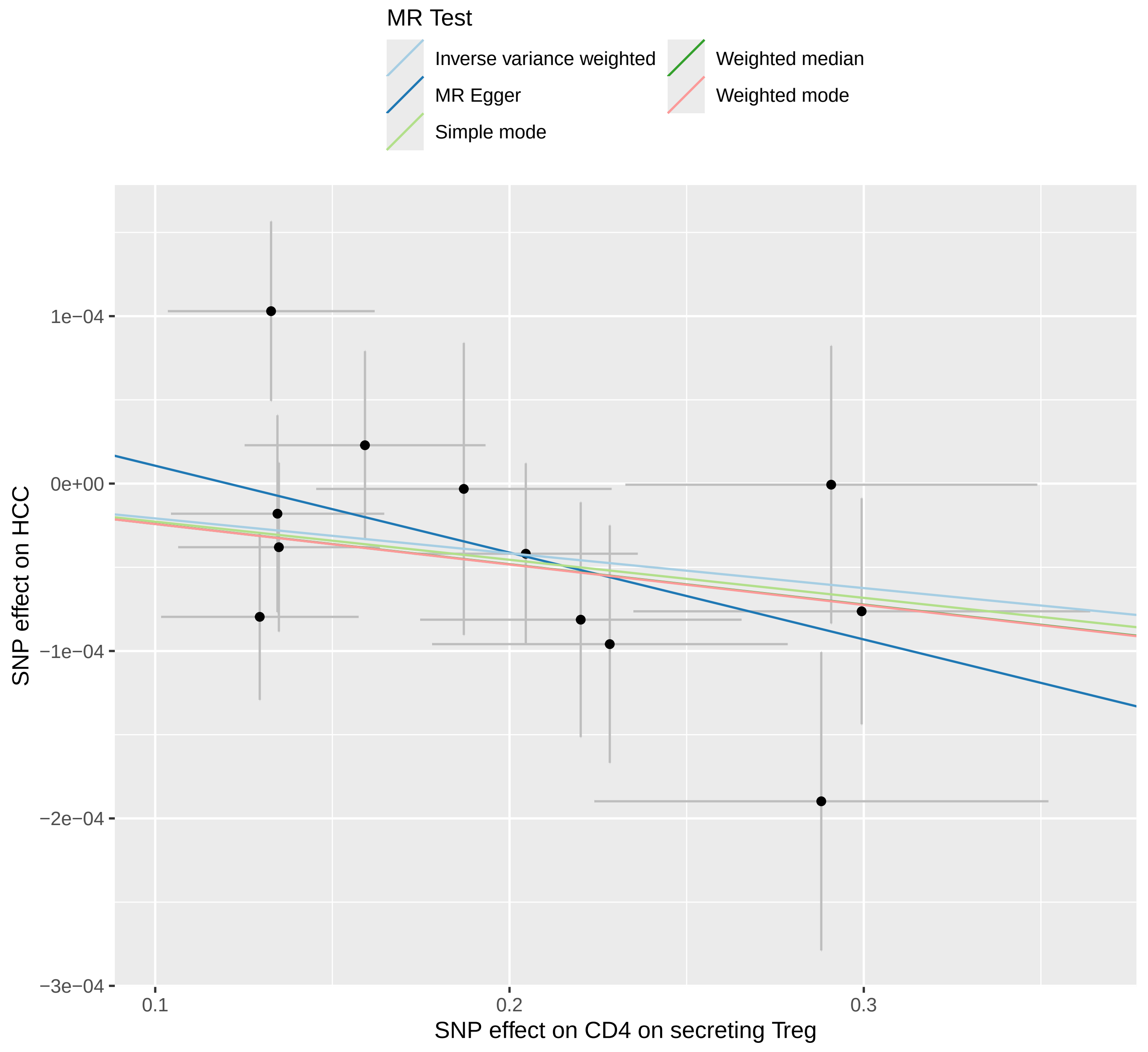
MR Test
Inverse variance weighted
MR Egger
Simple mode
Weighted median
Weighted mode
1e−04
0e+00
−1e−04
−2e−04
−3e−04
SNP effect on HCC
0.1
0.2
0.3
SNP effect on CD4 on secreting Treg

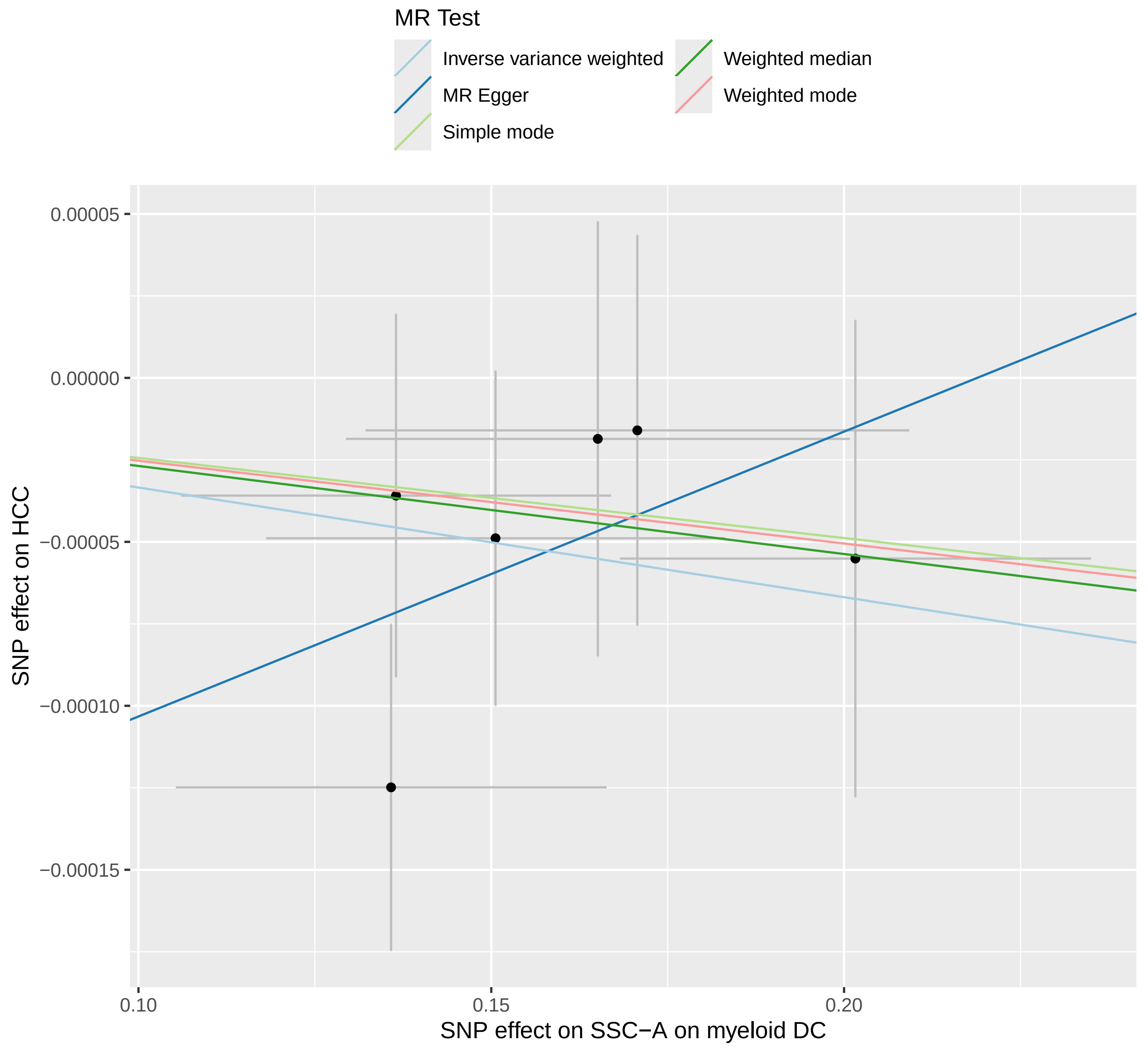

MR Test
Inverse variance weighted
MR Egger
Simple mode
Weighted median
Weighted mode
0.00005
0.00000
−0.00005
−0.00010
−0.00015
SNP effect on HCC
0.10
0.15
0.20
SNP effect on SSC−A on myeloid DC

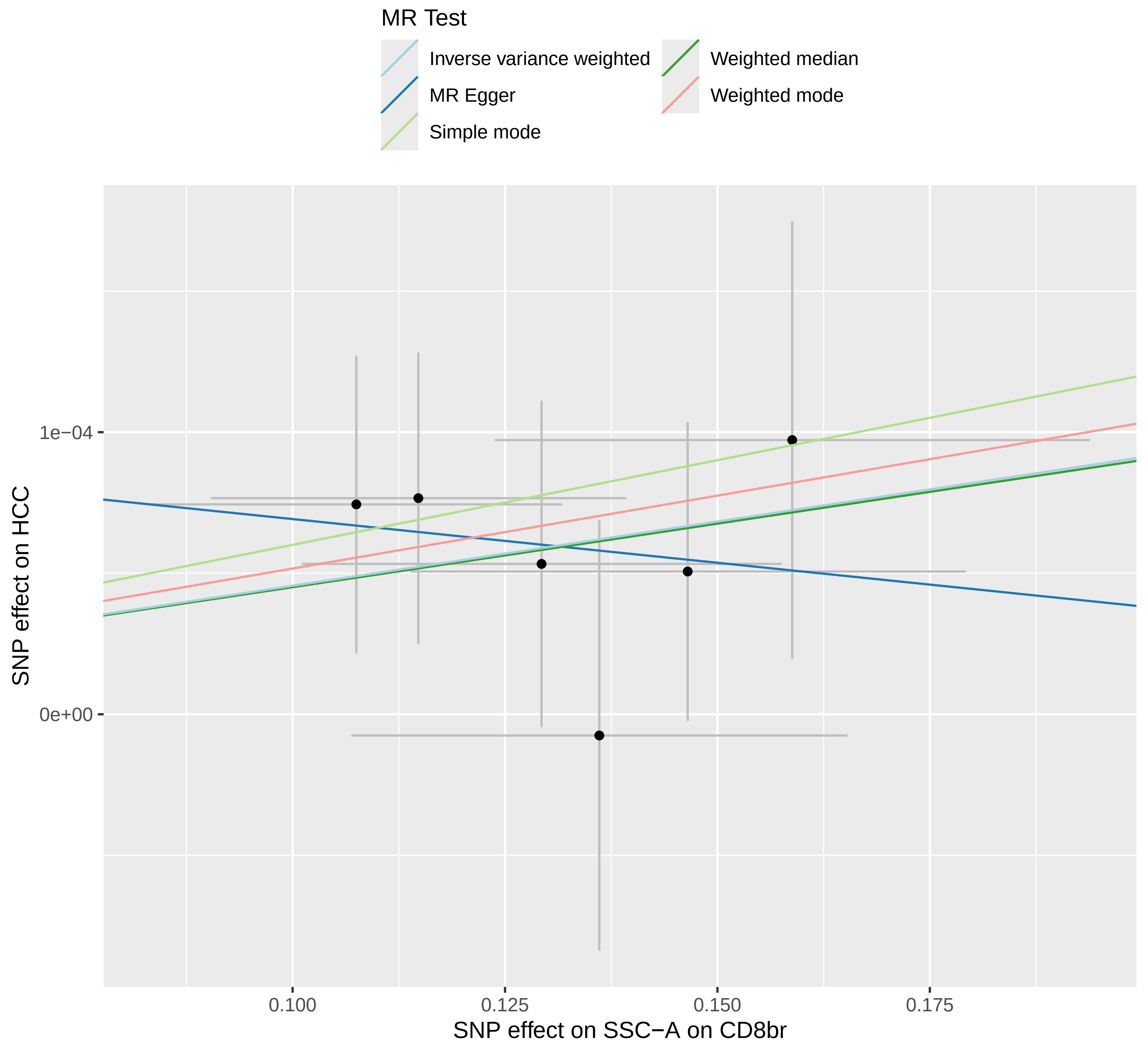
MR Test
Inverse variance weighted
MR Egger
Simple mode
Weighted median
Weighted mode
1e−04
0e+00
SNP effect on HCC
0.100
0.125
0.150
0.175
SNP effect on SSC−A on CD8br

Supplementary table4 ROC curve summarizing the Random forest results of immune cells with a causal relationship to HCC.

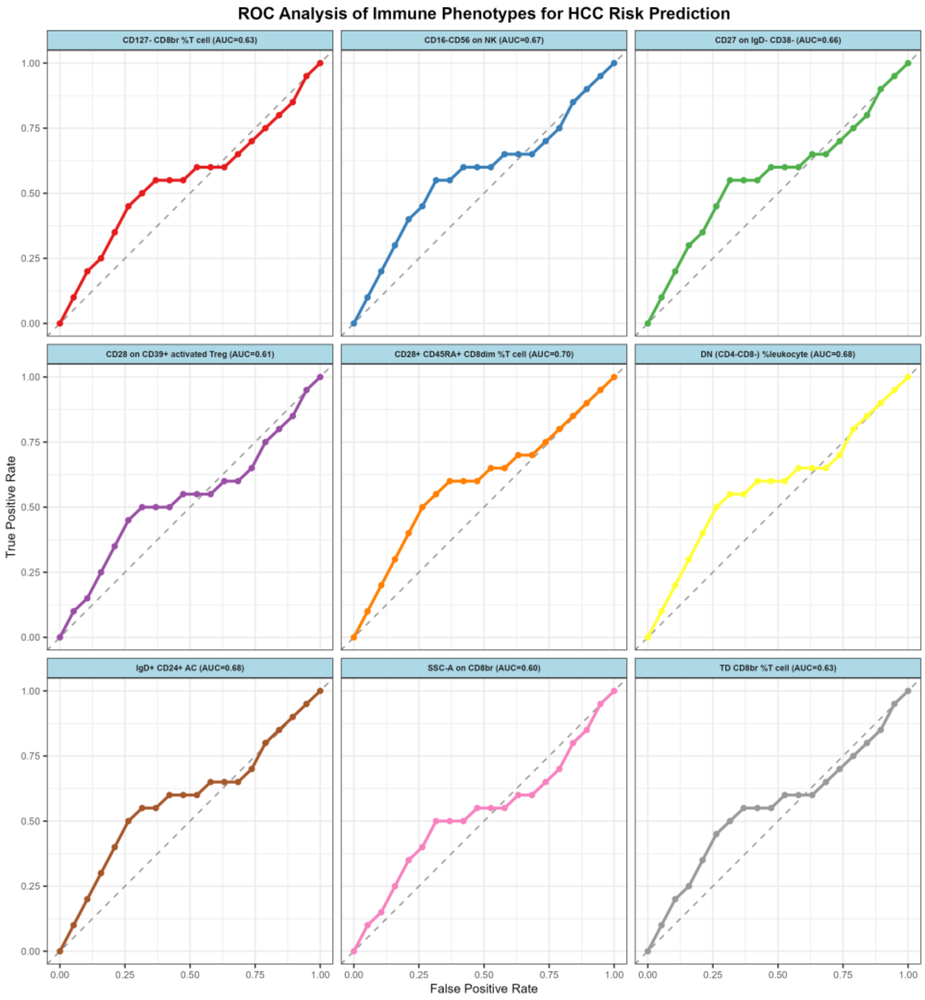

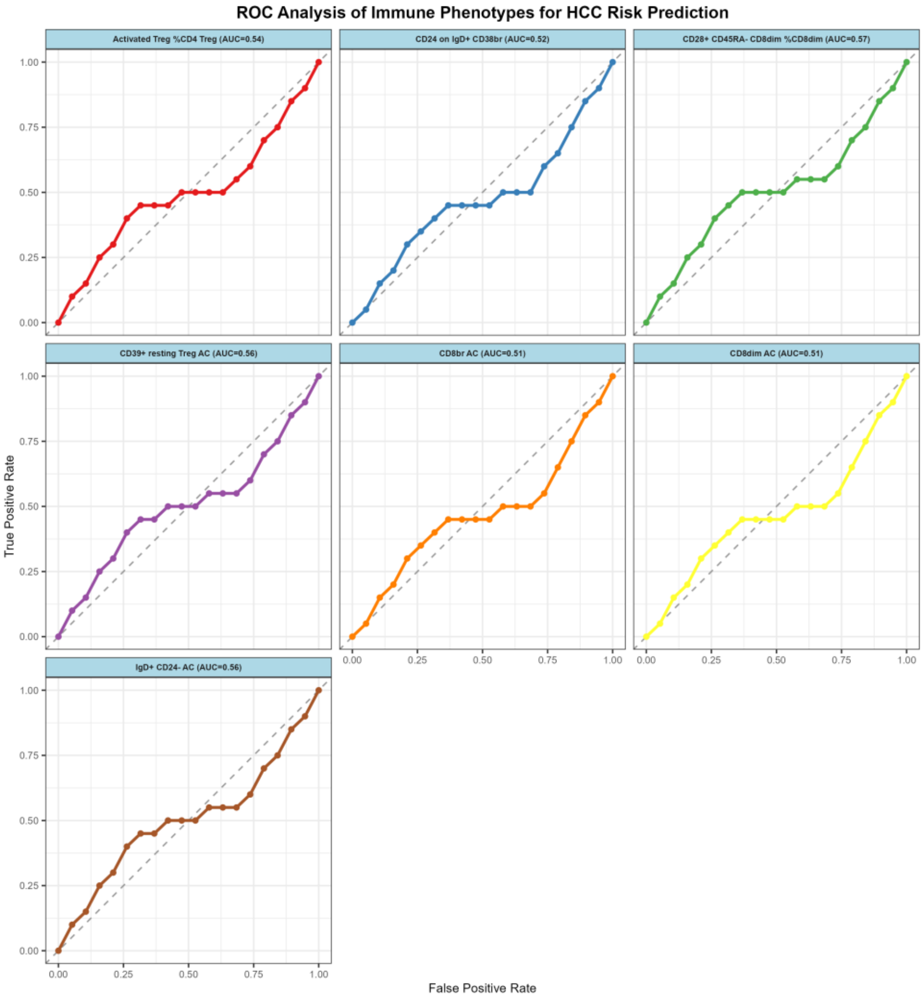
ROC Analysis of Immune Phenotypes for HCC Risk Prediction
Activated Treg %CD4 Treg (AUC=0.54)
CD24 on IgD+ CD38br (AUC=0.52)
CD28+ CD45RA- CD8dim %CD8dim (AUC=0.57)
CD39+ resting Treg AC (AUC=0.56)
CD8br AC (AUC=0.51)
CD8dim AC (AUC=0.51)
IgD+ CD24- AC (AUC=0.56)
True Positive Rate
False Positive Rate
0.00
0.25
0.50
0.75
1.00
